# Synthesis, Luminescence, and Electrochemistry of Tris-Chelate Platinum(IV) Complexes with Cyclometalated N-Heterocyclic Carbene Ligands and Aromatic Diimines

**DOI:** 10.1021/acs.inorgchem.4c04446

**Published:** 2024-12-19

**Authors:** José Serrano-Guarinos, Adrián Jiménez-García, Delia Bautista, Pablo González-Herrero, Ángela Vivancos

**Affiliations:** † Departamento de Química Inorgánica, Facultad de Química, Universidad de Murcia, Campus de Espinardo, 19, 30100 Murcia, Spain; ‡ Área Científica y Técnica de Investigación, Universidad de Murcia, Campus de Espinardo, 21, 30100 Murcia, Spain

## Abstract

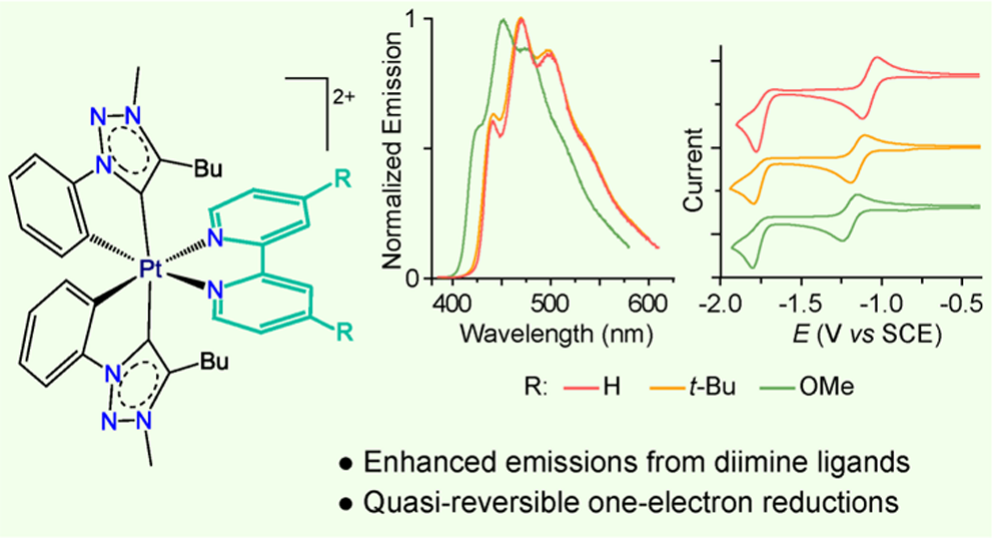

Dicationic, *C*_2_-symmetrical, tris-chelate Pt(IV) complexes of general formula [Pt(trz)_2_(N∧N)](OTf)_2_, bearing two cyclometalated 4-butyl-3-methyl-1-phenyl-1*H*-1,2,3-triazol-5-ylidene (trz) ligands and one aromatic diimine [N∧N = 2,2′-bipyridine (bpy, **2**), 4,4′-di-*tert*-butyl-2,2′-bipyridine (dbbpy, **3**), 4,4′-dimethoxi-2,2′-bipyridine (dMeO-bpy, **4**), 1,10-phenanthroline (phen, **5**), 4,7-diphenyl-1,10-phenanthroline (bphen, **6**), dipyrido[3,2-*a*:2′,3′-*c*]phenazine (dppz, **7**), or 2,3-diphenylpyrazino[2,3-*f*][1,10]phenanthroline (dpprzphen, **8**)] are obtained through chloride abstraction from [PtCl_2_(trz)_2_] (**1**) using AgOTf in the presence of the corresponding diimine. Complexes **2–4** show long-lived phosphorescence from ^3^LC excited states involving the diimine ligand, with quantum yields that reach 0.18 in solution and 0.58 in the solid matrix at room temperature for **3**. Derivatives with more extended aromatic systems show dual phosphorescent/fluorescent emissions (**5**, **6**) or mainly fluorescence (**7**, **8**) in solution. Comparisons with similar complexes bearing cyclometalated 2-arylpyridines instead of aryl-N-heterocyclic carbenes indicate that the {Pt(trz)_2_} subunit is crucial to enable efficient emissions from diimine-centered excited states. It is also shown that the introduction of protective bulky substituents on the diimine, such as the *tert*-butyl groups in **3**, is a key strategy to reach higher emission efficiencies. The new compounds represent rare examples of luminescent Pt(IV) complexes that show quasi-reversible one-electron reductions, indicating an unusually high redox stability.

## Abstract 2

Dicationic, *C*_2_-symmetrical Pt(IV) tris-chelates bearing two cyclometalated aryl-NHC ligands (C∧C*) and one aromatic diimine (N∧N) are reported. The {Pt(C∧C*)_2_} substructure is crucial to enable efficient phosphorescent emissions from ^3^LC(N∧N) excited states for the diimines with the least extended aromatic systems. Quasi-reversible one-electron reductions indicate an unusually high redox stability.

## Introduction

Chelating aromatic diimines, such as 2,2′-bipyridines, 1,10-phenanthrolines, and related heteroaromatic compounds, have played a key role in the historical development of luminescent complexes^1,2^ and are still widely employed today.^3,4^ The coordination of these compounds to second- and third-row late transition-metal ions gives rise to relatively rigid systems, particularly so in the case of tris-chelate structures, that possess accessible and long-lived emissive excited states resulting from electronic promotions to π* orbitals of the diimine, such as metal-to-ligand charge-transfer (MLCT or *d*-π*) or ligand-centered (LC or π–π*) transitions. In addition, they are often capable of reversible redox processes both in the ground and the excited state. This combination of photophysical and electrochemical characteristics, together with the possibility to modulate them through structural modifications, makes diimine complexes highly valuable for diverse applications, including luminescence-based sensing and imaging,^5−11^ photoredox catalysis,^12−14^ photodynamic chemotherapy,^15^ and the fabrication of electroluminescent devices.^16,17^

In recent decades, N-heterocyclic carbene ligands (NHCs) have become crucial constituents of highly efficient luminescent transition-metal complexes because of their strong σ-donating ability, which enhances the stability of the metal complex and, additionally, induces large ligand-field splittings, thereby elevating the energies of dissociative, metal-centered (MC) excited states and diminishing the nonradiative deactivation that often occurs through the thermal population of such states.^18−26^ In particular, cyclometalated aryl-NHC ligands (aryl-NHCs, C∧C*) have been profusely employed to develop Ir(III)- and Pt(II)-based dopants for electroluminescent materials.^27,28^ Mesoionic NHCs stand out in this area because of their remarkably strong σ-donating ability, as compared to normal, Arduengo-type NHCs.^29,30^ The properties of NHC ligands have even been exploited with success to prolong the lifetimes of MLCT excited states of first-row transition-metal complexes,^31−37^ which are strongly deactivated via low-lying MC excited states.

In comparison with other d^6^ transition-metal complexes, much less focus has been placed on developing luminescent Pt(IV) complexes. Because of the high oxidation state and electrophilic character of the Pt(IV) ion, its complexes tend to present low-lying ligand-to-metal charge-transfer (LMCT) excited states involving promotions to dσ* orbitals that, when populated via photoexcitation, can cause nonradiative deactivation, ligand dissociation or reduction to Pt(II).^38^ During the past decade, we and others have reported several series of luminescent Pt(IV) complexes bearing cyclometalated 2-arylpyridines (C∧N) and related ligands, which can reach high luminescence efficiencies from ^3^LC states with a very small MLCT character.^39−48^ The key to achieving photostability and emission efficiency from Pt(IV) complexes is the presence of at least two metalated carbon atoms, which provide a strong σ donation that elevates the energies of dσ* orbitals and LMCT states, reducing their adverse effects. Thus, facial tris-cyclometalated complexes, *fac*-[Pt(C∧N)_3_]^+^,^39,46^ and bis-cyclometalated complexes with a supporting metalated aryl or alkyl ligand, [Pt(C∧N)_2_(Ar/R)(X)],^41,48^ are among the strongest Pt(IV) emitters, whereas bis-cyclometalated Pt(IV) complexes without supporting strong-field ligands typically exhibit weak emissions and some of them may show photochemical reactivity.^49,50^ With the aim to leverage the strongly σ-donating capabilities of NHC ligands for the design of Pt(IV) emitters, we recently developed synthetic routes to complexes of the types [PtCl_2_(trz)(C∧N)]^51,52^ and [Pt(trz)_2_(C∧N)]^+^,^53^ where trz is the cyclometalated, mesoionic aryl-NHC 4-butyl-3-methyl-1-phenyl-1*H*-1,2,3-triazol-5-ylidene acting as a nonchromophoric, supporting C∧C* ligand. These complexes showed enhanced emission efficiencies with respect to analogous complexes with only C∧N ligands and the same arrangement of metalated aryl moieties. In particular, tris-chelate complexes [Pt(trz)_2_(C∧N)]^+^, having a meridional arrangement of metalated aryl groups, showed significant photostability and intense phosphorescent emissions from ^3^LC(C∧N) states, in sharp contrast with homoleptic *mer*-[Pt(C∧N)_3_]^+^ complexes, which are photoreactive. Therefore, it was shown that the {Pt(trz)_2_} subunit has great potential as a platform for the development of phosphorescent Pt(IV) tris-chelates by incorporating different types of chromophoric bidentate ligands.

Early studies on the photophysical properties and photochemistry of Pt(IV) complexes bearing aromatic diimines showed that they are often light-sensitive. For example, [PtMe_4_(bpy)] (bpy = 2,2′-bipyridine) undergoes homolysis of a Pt–C bond upon irradiation with visible light.^54^ Complex [PtMe_4_I(bpy)] emits in fluid ethanol solution at room temperature from a LC(N∧N) state, but it gradually undergoes reduction to [PtMeI(bpy)] upon photoexcitation.^55^ Complexes [PtCl_2_(N∧N)_2_]^2+^ [N∧N = bpy or 1,10-phenanthroline (phen)] and [PtCl_2_(en)(bpy)]^2+^ (en = 1,2-ethanediamine) also undergo photodecomposition to Pt(II) species and their emissions where characterized in solution at 77 K and attributed to LC(N∧N) states.^56^ More recently, complexes [PtMe_3_(N∧N)(SR)], N∧N = bpy, 4,4′-di(methoxycarbonyl)-2,2′-bipyridine; R = MeCO_2_C_6_H_4_ were shown to emit in the red or NIR from σ(S–Pt) → π*(N∧N) states and demonstrated as bioimaging agents;^57^ however, a study on an extended series of analogous complexes showed that some of them undergo the aerobic oxidation of the thiolate ligand under photochemical conditions to give sulphinate derivatives [PtMe_3_(N∧N)(SO_2_R)], which show very weak luminescence that was attributed to σ(S–Pt/C–Pt) → π*(N∧N) excited states.^58^

Luminescent *C*_2_-symmetrical dicationic tris-chelate Pt(IV) complexes bearing two cyclometalated heteroaromatic C∧N ligands and one aromatic diimine, [Pt(C∧N)_2_(N∧N)]^2+^, have been reported by different authors.^59−61^ Complexes of this type featuring 2-arylpyridines as the C∧N ligands and bpy derivatives as the N∧N ligand showed generally weak phosphorescent emissions in fluid solution at room temperature, arising from ^3^LC states involving the C∧N ligands; their electrochemical investigation revealed an irreversible reduction, which was interpreted to lead to bis-cyclometalated Pt(II) complexes.^59^ Recently, two more series were investigated, one of them bearing cyclometalated 2-phenylbenzothiazol (pbt) ligands and phen-based diimines,^60^ and the other with cyclometalated 1-phenylpyrazole (ppz) ligands and bpy- and phen-derived diimines.^61^ The pbt complexes presented very weak emissions in solution and solid matrices at room temperature from ^3^LC(pbt) states or, in some cases, dual emissions from ^3^LC(pbt) and ^3^LC(N∧N) states; they also presented irreversible electrochemical reductions. The ppz complexes showed luminescence at 77 K from ^3^LC(N∧N) states but were almost nonemissive at room temperature due to strong deactivation via ligand-to-ligand charge-transfer (LLCT) or LMCT excited states.

Based on our previously developed methodology for the synthesis of the bis-cyclometalated Pt(IV) complex [PtCl_2_(trz)_2_] and its successful use as precursor for the preparation of phosphorescent tris-chelate complexes of the type [Pt(trz)_2_(C∧N)]^+^,^53^ for the present work, we aimed at exploring the {Pt(trz)_2_} platform for the development of Pt(IV) tris-chelates incorporating diimines as the chromophoric ligands, [Pt(trz)_2_(N∧N)]^2+^, in the expectation that the strong electron-donating capabilities of the trz ligands would lead to enhanced photostabilities and emission efficiencies with respect to previous Pt(IV) tris-chelates that combine C∧N and diimine ligands. Our study includes diimines with extended aromatic systems, which have been widely employed for the development of bioactive metal complexes for use as DNA intercalators, biological sensors, and theranostic agents.^62−66^ We present a systematic evaluation of the structural, photophysical and electrochemical properties of the new complexes.

## Results and Discussion

### Synthesis and Characterization

**Scheme 1:**
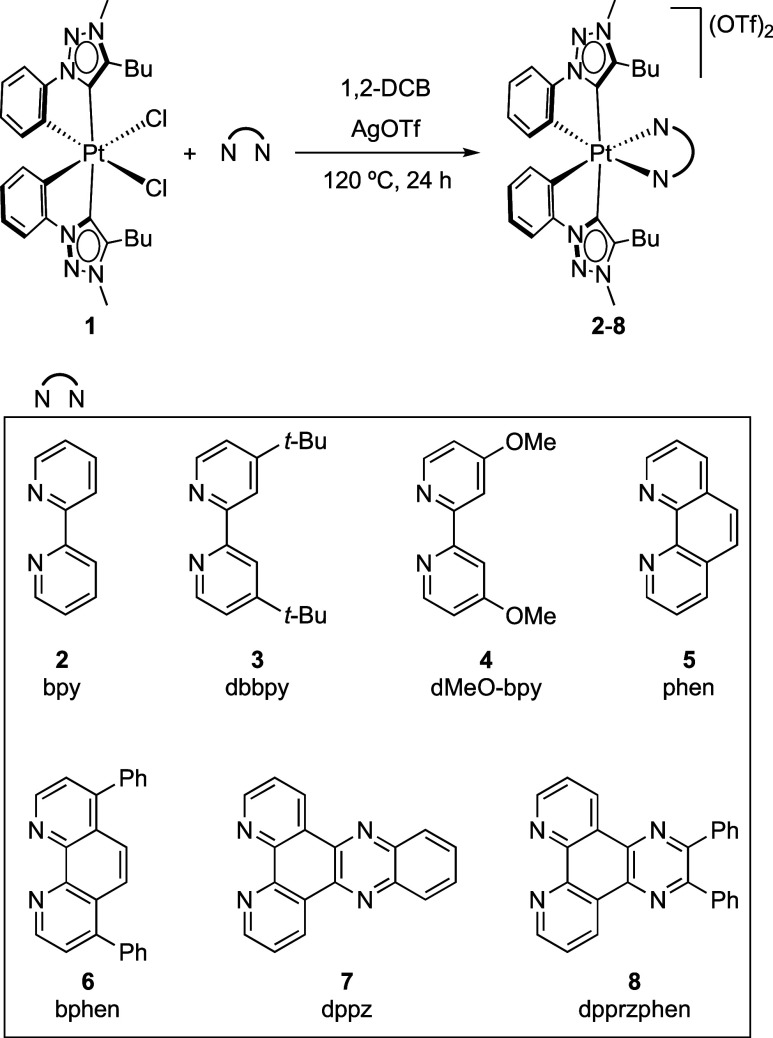
Synthesis of Complexes **2–8** 1,2-DCB = 1,2-dichlorobenzene.

The synthesis of the dicationic tris-chelate Pt(IV) complexes [Pt(trz)_2_(N∧N)]^2+^ (**2**–**8**) and the employed aromatic diimines are shown in Scheme 1. The substitution of the chloride ligands of the bis-cyclometalated complex [PtCl_2_(trz)_2_] (**1**)^53^ by the diimines was achieved using a slight excess of silver triflate (2.4 equiv) in the presence of the aromatic diimine and heating at 120 °C in 1,2-dichlorobenzene for 18 h. All complexes were obtained as white solids in good to moderate yields (67–92%) after filtration and recrystallization protocols.

The electrospray ionization mass spectra in positive mode show two peaks for all derivatives; the most intense one corresponds to the dication [Pt(trz)_2_(N∧N)]^2+^, with *m*/*z* values of half the molecular mass, while the other corresponds to the cation [Pt(trz)_2_(N∧N)(OTf)]^+^. The expected *C*_2_ symmetry of the complexes is demonstrated by their ^1^H and ^13^C{^1^H} NMR spectra, which show only one set of resonances from the cyclometalated trz ligands in all cases. The protons ortho to the metalated carbon of the phenyl rings give rise to a characteristic doublet of doublets with platinum satellites (*J*_PtH_ ∼ 47 Hz) in the ^1^H NMR spectra. This signal is upfield-shifted with respect to the rest of aromatic resonances (6.67–6.82 ppm) because it is affected by the diamagnetic current of an orthogonal aromatic ring. In the ^13^C{^1^H} NMR spectra we can find a resonance around 143.5 ppm flanked by platinum satellites (*J*_PtC_ ∼ 800 Hz) that can be attributed to the carbenic carbon, whereas the metalated phenyl carbons appear in all cases between 119.6 and 120.6 ppm with a *J*_PtC_ coupling constant in the range 750–760 Hz, with the exception of complex **2**, for which the corresponding *J*_PtC_ constant could not be measured.

**Figure 1:**
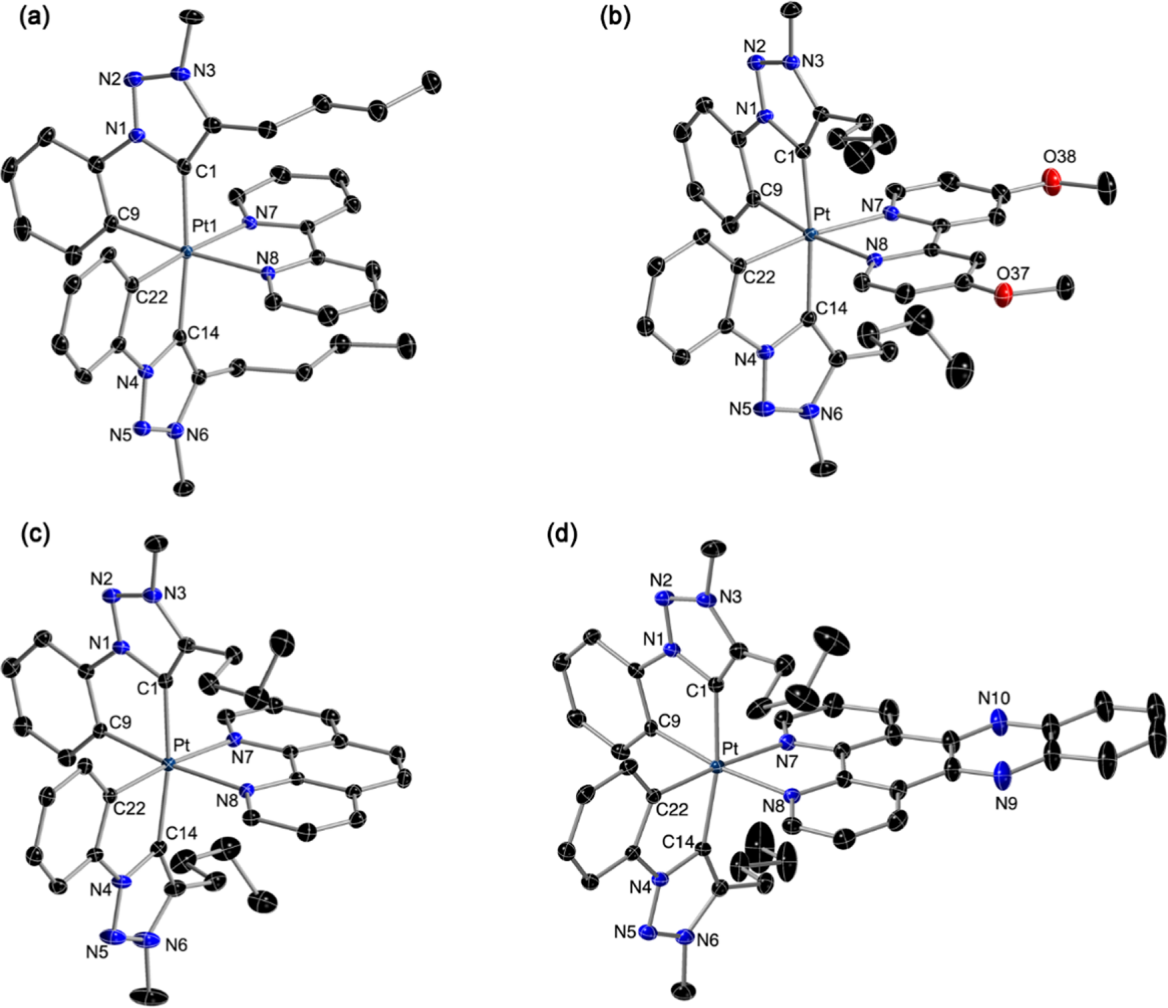
Molecular structures of the dications of compounds **2** (a), **4** (b), **5** (c), and **7** (d) in the crystal (thermal ellipsoids at 50% probability). Hydrogen atoms are omitted.

The crystal structures of compounds **2**, **4**·1.5CH_2_Cl_2_, **5**·CH_2_Cl_2_, and **7**·Me_2_CO·Et_2_O were determined by X-ray diffraction, and the molecular structures of the dications are shown in Figure 1. Selected bond lengths and angles are compiled in Table 1. As expected, all complexes present the nitrogen atoms of the N∧N ligand *trans* to the metalated carbons of the trz ligands, and the carbenic moieties are mutually *trans*. In all cases, the Pt-carbene bond lengths (Pt–C1, Pt–C14) are similar to those found for the tris-cyclometalated complexes [Pt(trz)_2_(C∧N)]^+^,^53^ which present the same disposition of trz ligands; these distances are slightly longer than those involving the metalated phenyl carbons (Pt–C9, Pt–C22). The lower trans influence of the diimines compared to the cyclometalated C∧N ligands makes these Pt–C9/C22 bonds shorter relative to the tris-cyclometalated complexes [Pt(trz)_2_(C∧N)]^+^ (values between 2.0414 and 2.0837 Å). The molecules of **7** stack in pairs (Figure S1) due to intermolecular π interactions between dppz rings (distance between mean dppz planes: 3.347 Å). The other structures did not show similar π interactions.

**Table 1:** Selected Distances (Å) and Angles (deg) for Complexes **2**, **4**, **5**, and **7**

	**2**	**4**	**5**	**7**
Pt–C1	2.047(3)	2.045(3)	2.046(2)	2.051(3)
Pt–C9	2.040(3)	2.029(2)	2.024(2)	2.027(3)
Pt–C14	2.049(3)	2.049(3)	2.052(2)	2.051(3)
Pt–C22	2.031(3)	2.032(3)	2.034(2)	2.031(3)
Pt–N7	2.126(2)	2.118(2)	2.1177(17)	2.124(2)
Pt–N8	2.119(2)	2.108(2)	2.1224(18)	2.118(2)
C1–Pt–C9	80.62(11)	80.79(10)	80.72(8)	80.78(11)
C14–Pt–C22	80.50(11)	80.75(10)	80.69(8)	80.63(11)
N7–Pt–N8	77.72(8)	77.24(8)	79.01(7)	78.40(9)
C1–Pt–C14	173.99(10)	173.94(10)	171.20(8)	169.94(10)

### Photophysical Properties

**Figure 2:**
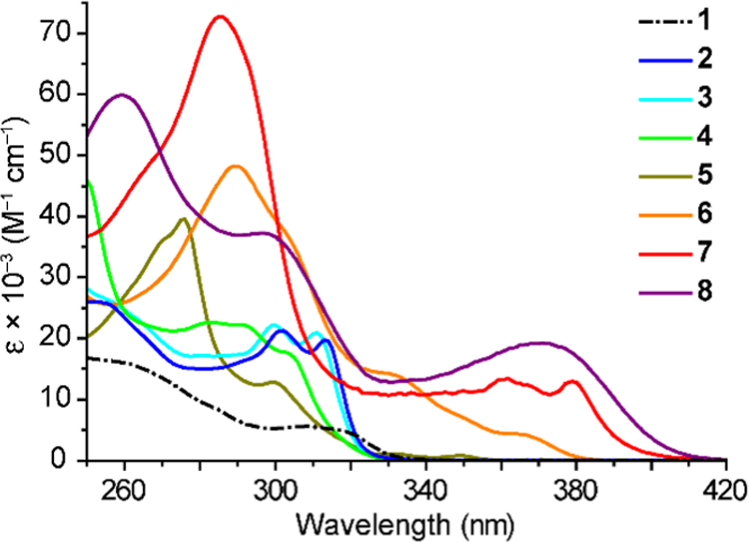
Electronic absorption spectra of complexes **1**–**8** in a CH_2_Cl_2_ solution (ca. 5 × 10^–5^ M) at 298 K.

The electronic absorption spectra of complexes **2**–**8** were registered in CH_2_Cl_2_ solution at 298 K and are shown in Figure 2. The absorption data are summarized in Table 2. The data for the precursor **1** are included for comparison. The major features observed in the spectra of **2**–**8** are clearly attributable to the diimine ligands, since they are more intense and different in shape as compared to those found in the spectrum of **1**. The lowest-energy (LE) band displays vibrational structure in most cases and can be ascribed to the lowest π–π* singlet transition centered on the diimine. In the series of complexes bearing a bpy-based diimine (**2**–**4**), the introduction of electron-donating substituents (*t*-Bu, MeO) causes a hypsochromic shift of this band. Among the phen-based derivatives, significant bathochromic shifts are observed as the aromatic system is extended (**6**, **7**, **8**). The shape and energies of the LE bands are similar to those found for analogous *C*_2_-symmetrical complexes of the type [Pt(C∧N)_2_(N∧N)]^2+^ bearing cyclometalated 1-phenylpyrazol^61^ and the same N∧N ligands. Complexes **5**–**8** give rise to more intense absorptions in the 250–360 nm range that can be mainly ascribed to strongly allowed π–π* transitions within the diimine.

**Table 2:** Electronic Absorption Data for the Studied Complexes in CH_2_Cl_2_ Solution (ca. 5 × 10^–5^ M) at 298 K

complex	λ_max_ (nm) (ε × 10^–3^ (M^–1^ cm^–1^))
**1**	260 (16), 307 (56)
**2**	255 (26), 301 (21), 313 (20)
**3**	257 (26), 299 (22), 311 (21)
**4**	250 (46), 283 (23), 291 (22), 303 (18)
**5**	270 (43), 276 (48), 300 (15), 334 (1), 349 (1)
**6**	289 (48), 303 (37), 332 (14), 366 (4)
**7**	285 (73), 361 (13), 379 (13)
**8**	259 (60), 297 (37), 371 (19)

**Figure 3:**
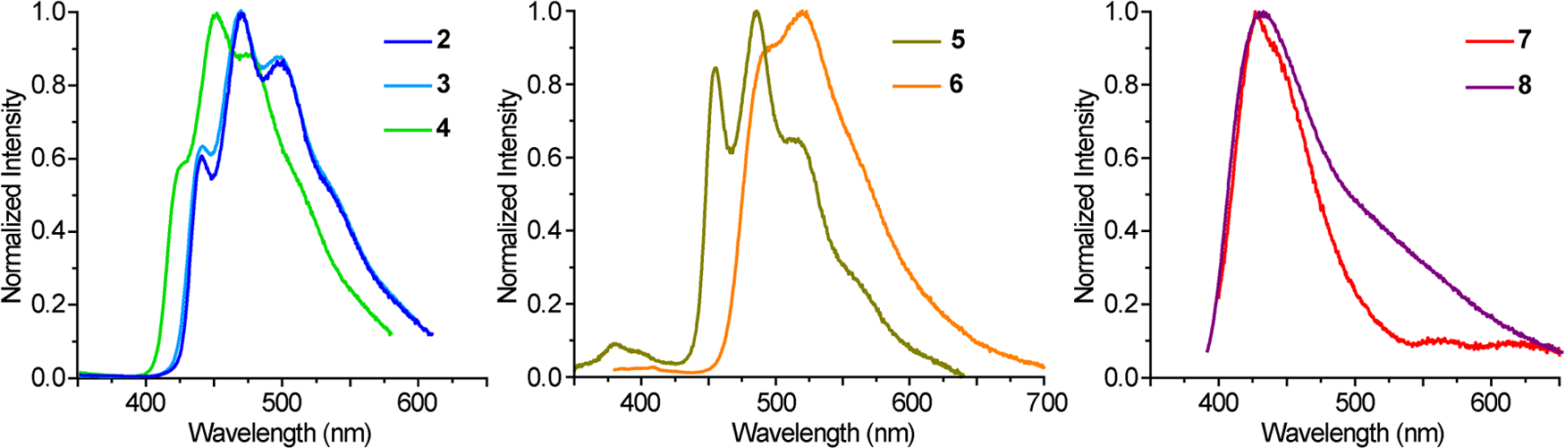
Emission spectra of complexes **2**–**8** in a CH_2_Cl_2_ solution (ca. 5 × 10^–5^ M) at 298 K.

Before examining the emission properties of **2**–**8**, the photostability of two representative complexes (**3** and **5**) in CH_2_Cl_2_ solution was evaluated by irradiating them at the lowest-energy absorption wavelength for 30 min, which did not cause any change in the absorption spectra (Figure S9). The luminescence of complexes **2**–**8** was studied in degassed CH_2_Cl_2_ solutions and poly(methyl methacrylate) (PMMA) matrices (2 wt %) at 298 K. The emission spectra in solution are shown in Figure 3, and the data are summarized in Table 3. The complete set of excitation and emission spectra is included in the Supporting Information. Complexes **2**–**4** present structured emission bands in both media, with the highest-energy peak at 440 (**2** and **3**) or 426 nm (**4**) and lifetimes in the tens of microseconds range in solution and hundreds of microseconds in PMMA, which is in consonance with phosphorescent emissions from a ^3^LC state involving the diimine ligand. The emission energies of **2** and **3** are very similar, indicating a negligible effect of the *t-*Bu substituents, whereas a significant hypsochromic shift is observed for **4**, bearing methoxy substituents, as was also noticed in the absorption spectra. The quantum yields of complexes **2** and **4** are similar, with values around 0.02 in solution and 0.40 in PMMA. However, the emission efficiency of **3** is considerably higher, reaching 0.18 in CH_2_Cl_2_ solution and 0.58 in PMMA. Probably, the *t*-Bu substituents exert a protective effect in solution, reducing the direct collisions between the aromatic rings of the diimine and the solvent that could facilitate nonradiative deactivation. In fact, this is reflected in a decrease in the nonradiative rate constant (*k*_nr_) in solution by an order of magnitude with respect to **2** and **4**. In comparison with complexes of the type [Pt(C∧N)_2_(N∧N)]^2+^,^59^ which bear cyclometalated 2-arylpyridines (C∧N) instead of the aryl-NHC ligands, and bpy or 4,4′-dimethoxy-2,2′-bipyridine (dMeO-bpy) as the N∧N ligands, derivatives **2** and **4** present significantly higher emission efficiencies. Thus, quantum yields increase from 0.0029 to 0.026 (bpy derivatives) or 0.011 to 0.022 (dMeO-bpy derivatives) upon replacement of the C∧N ligands with trz. A more direct comparison can be established between **2** and [Pt(ppz)_2_(bpy)]^2+^, both emitting from a ^3^LC(bpy) state, but the latter has a much lower quantum yield (<0.001).^61^

**Table 3:** Emission Data for the Studied Complexes

complex	medium	λ_em_ (nm)^a^	Φ^b^	τ (μs)^c^	*k*_r_ × 10^–3^ (s^–1^)^d^	*k*_nr_ × 10^–3^ (s^–1^)^e^
**2**	CH_2_Cl_2_	440, *470*, 499	0.026	38	0.7	26
	PMMA	440, *470*, 498	0.40	376	1.1	1.6
**3**	CH_2_Cl_2_	440, *469*, 497	0.18	191	0.9	4.3
	PMMA	440, *467*, 495	0.58	418	1.4	1.0
**4**	CH_2_Cl_2_	426, *451*, 476	0.022	17	1.3	58
	PMMA	426, *452*, 475	0.37	308	1.2	2.1
**5**	CH_2_Cl_2_	377*, 455, *485*, 520	0.004	25	0.2	40
	PMMA	417*, 454, *483*, 517	0.14	979	0.1	0.9
**6**	CH_2_Cl_2_	493, *520*	0.035	116	0.3	8.3
	PMMA	421*, 442*, *488*, 514	0.31	1980 (83%), 675 (17%)	0.2	0.4
**7**	CH_2_Cl_2_	428	0.002	–f	–	–
	PMMA	420*, 436*, *555*, 600	0.006	–f	–	–
**8**	CH_2_Cl_2_	429	0.007	–f	–	–
	PMMA	413*, 527	0.023	–f	–	–

a The most intense peak is italicized; fluorescence peaks are marked with an asterisk for dual fluorescent/phosphorescent emitters.

b Quantum yield.

c Lifetime.

d Radiative rate constant, *k*_r_ = Φ/τ.

e Nonradiative rate constant, *k*_nr_ = (1−Φ)/τ.

f Could not be determined.

The main emissions observed from **5** and **6** are attributable to phosphorescence from ^3^LC(N∧N) states in view of their long lifetimes, which reach the ms range in PMMA. These emissions are shifted to lower energies with respect to complexes **2**–**4** due to the more extended π conjugation of the phen and bphen ligands relative to the bpy-based ligands. In the case of **5**, a well-defined vibronic structure is observed, with the highest-energy peak at 455 nm, whereas the phenyl substituents in complex **6** cause a shift to 493 nm and a weaker vibronic definition. In addition, a weaker band at higher energies is observed for both complexes, with lifetimes shorter than the measurement limit of our equipment (<0.2 ns), which can be attributed to fluorescence. Dual fluorescence/phosphorescence emissions have been previously observed from Pt(IV) complexes bearing cyclometalated C∧N ligands with extended aromatic systems, such as 1-phenylisoquinoline or 2-(9,9-dimethylfluoren-2-yl)pyridine, and can be explained by the lower proportion of metal orbital contribution to the emissive excited state, which reduces the spin–orbit coupling effects and the intersystem crossing rates to the triplet manifold, thereby making fluorescence competitive.^40,46,67^ Compared to **2**–**4**, the emission of **5** is considerably weaker both in solution and PMMA matrix. The phenyl substituents on the bphen ligand in **6** have a beneficial effect, with quantum yields 1 order of magnitude higher in solution and three times higher in PMMA with respect to **5**.

Complexes **7** and **8** show an unstructured emission band with a maximum at ca. 428 nm in CH_2_Cl_2_ and very low quantum yields. The small Stokes shifts indicate fluorescence from a ^1^LC(N∧N) state. An additional feature is observed for **8** at λ > 480 nm, which we attribute to a weak phosphorescent emission due to its lower energy and the similarity of its excitation spectrum to that monitored at the main emission wavelength (Figure S10). Both complexes show two emission bands in the PMMA matrix. The high energy emissions are similar in energy to those observed in solution and therefore can be ascribed to fluorescence from a ^1^LC(N∧N) excited state. The low energy emissions are observed at around 555 (**7**) or 527 (**8**) nm (Figure S11) and can be attributed to phosphorescence from the ^3^LC(N∧N) state on the basis of the large Stokes Shifts and the fact the excitation spectra monitored at these wavelengths (Figure S11) reproduce the respective absorption profiles. Quantum yields are higher in this rigid medium, although they remain very low. Unfortunately, emission lifetimes could not be determined for any of the observed bands. Therefore, the main characteristics of the emissions of these derivatives are the strong nonradiative deactivation and a higher difficulty in the formation of the triplet excited state with respect to the complexes bearing diimines with less extended aromatic systems. The fact that the phosphorescence of **7** is observed in the rigid medium but not in fluid solution indicates that emission from the triplet excited state is much more easily deactivated through collisions with the solvent.

### Computational Study

**Figure 4:**
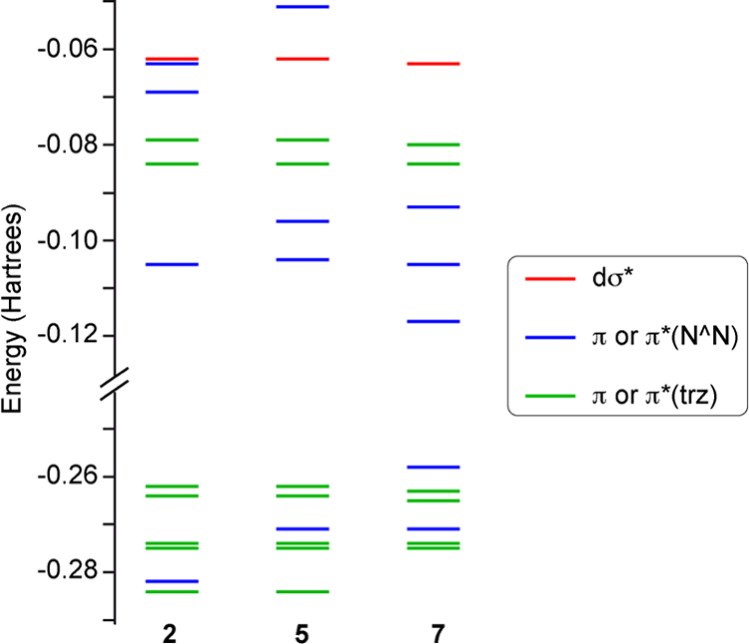
Molecular orbital energy diagram for complexes **2**, **5**, and **7** from DFT calculations.

For a more precise understanding of the observed properties, DFT and TD-DFT calculations have been carried out for derivatives **2**, **5**, and **7**. Complete details are presented in the Supporting Information, including frontier molecular orbital isosurfaces and listings of selected vertical singlet and triplet excitations. Figure 4 shows a frontier molecular orbital energy diagram with a color-coded representation of their main character. In complex **2**, the highest occupied molecular orbital (HOMO) and the next three occupied orbitals at lower energies (HOMO–1 to HOMO–3) are distributed over the metalated aryl rings of both trz ligands, with significant contribution from metal dπ orbitals in the HOMO (8%) and HOMO–2 (6%). The HOMO–4 is essentially the highest π orbital of the bpy ligand, whereas the lowest π* orbital of this ligand constitutes the lowest unoccupied molecular orbital (LUMO). These orbitals have a small but nonzero contribution of metal orbitals (less than 1%; see Figure S13). The trz-centered molecular orbitals in **5** and **7** have essentially the same composition and energies as those of **2** (Figure 4), whereas the main differences are found in the diimine-based orbitals. Thus, the highest occupied diimine π orbital increases its energy in the sequence **2** < **5** < **7** and becomes the HOMO in **7**. The LUMO in both **5** and **7** is the lowest diimine π* orbital, with a similar energy to that of **2** in the case of **5**, but a significantly lower energy in the case of **7**. The lowest molecular orbital with dσ* character is LUMO+5 (**2**, **7**) or LUMO+4 (**5**).

The TD-DFT calculations show that the four lowest singlet excitations in complex **2** involve promotions from the π orbitals of the trz ligands (HOMO to HOMO–3) to the LUMO, and can therefore be described as ligand-to-ligand charge-transfer (LLCT) transitions. However, their predicted oscillator strengths are extremely low. The lowest singlet excitations with significant oscillator strengths correspond to ^1^LC transitions within the trz (S_5_) and bpy (S_9_) ligands, the latter having a much higher intensity. Therefore, the lowest-energy band observed in the absorption spectrum of **2** is mainly attributable to a ^1^LC(bpy) transition, although it must obscure a less intense band arising from a ^1^LC(trz) transition. The lowest singlet excitations predicted for **5** also correspond to LLCT transitions with extremely low oscillator strengths (S_1_, S_2_). The lowest ^1^LC(phen) excitations (S_3_, S_8_) are predicted at lower energies and have much smaller oscillator strengths compared to the ^1^LC(bpy) excitations in **2**. This scenario is consistent with the observation of low-intensity bands in the range 300–350 nm in the experimental absorption spectrum of **5** and indicates a high degree of forbideness for the lowest π–π* transitions within the phen ligand. Several ^1^LC(dppz) excitations are predicted among the lowest singlet excitations for complex **7**, having low to moderate oscillator strengths, which are responsible for the lowest-energy band observed in the experimental absorption spectrum.

**Figure 5:**
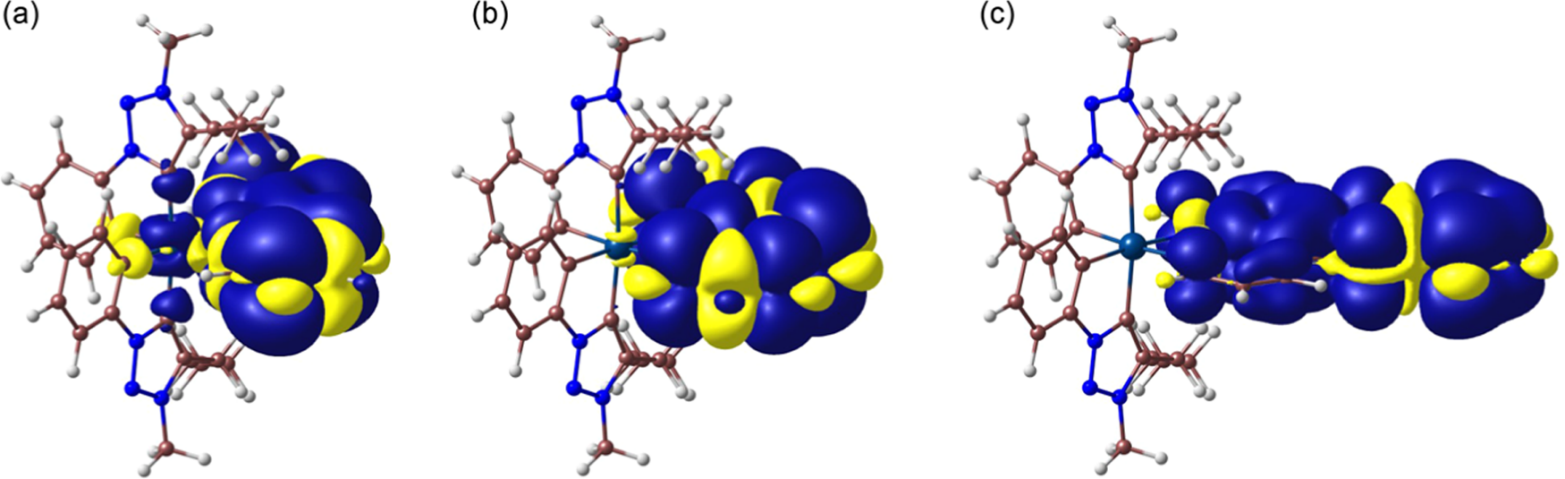
Spin density distributions (0.0002 e bohr^–3^) of the optimized lowest triplet excited state of complexes **2** (a), **5** (b), and **7** (c).

The first triplet excitation (T_1_) of **2**, **5**, and **7** corresponds to a ^3^LC transition within the diimine ligand, in agreement with the above-proposed assignment of their phosphorescent emissions. The lowest triplet excitations with significant LMCT character are T_13_ (**2**), T_11_ (**5**), or T_21_ (**7**), involving an electronic promotion to the lowest dσ* orbital, and are 1.01, 1.09, or 1.69 eV, respectively, above T_1_. These are significant energy differences, suggesting that thermal population of deactivating ^3^LMCT states from the emissive state should not have an important contribution to nonradiative decay (cf. 0.68, 0.78, or 1.26 eV for the emissive complexes [PtCl_2_(tpy)_2_] [tpy = cyclometalated 2-(*p*-tolyl)pyridine], [PtCl_2_(trz)(tpy)]^52^ or [Pt(trz)_2_(tpy)]^+^,^53^ respectively). The geometry of the lowest triplet excited state of the three complexes was optimized for further insight. The spin density distributions (Figure 5) are consistent with a π–π* transition within the diimine ligand with a very small contribution from metal orbitals, which decreases in the order **2** > **5** > **7**. It is thus clear that the more extended aromatic diimines lead to a lower participation of metal orbitals in the excited state, making the intersystem crossing between singlet and triplet states more difficult. The adiabatic energy differences with respect to the ground state are 2.76 eV (449 nm; **2**), 2.66 eV (467 nm; **5**), and 2.09 eV (594 nm; **7**), in good agreement with the experimental phosphorescent emissions.

### Electrochemistry

**Figure 6:**
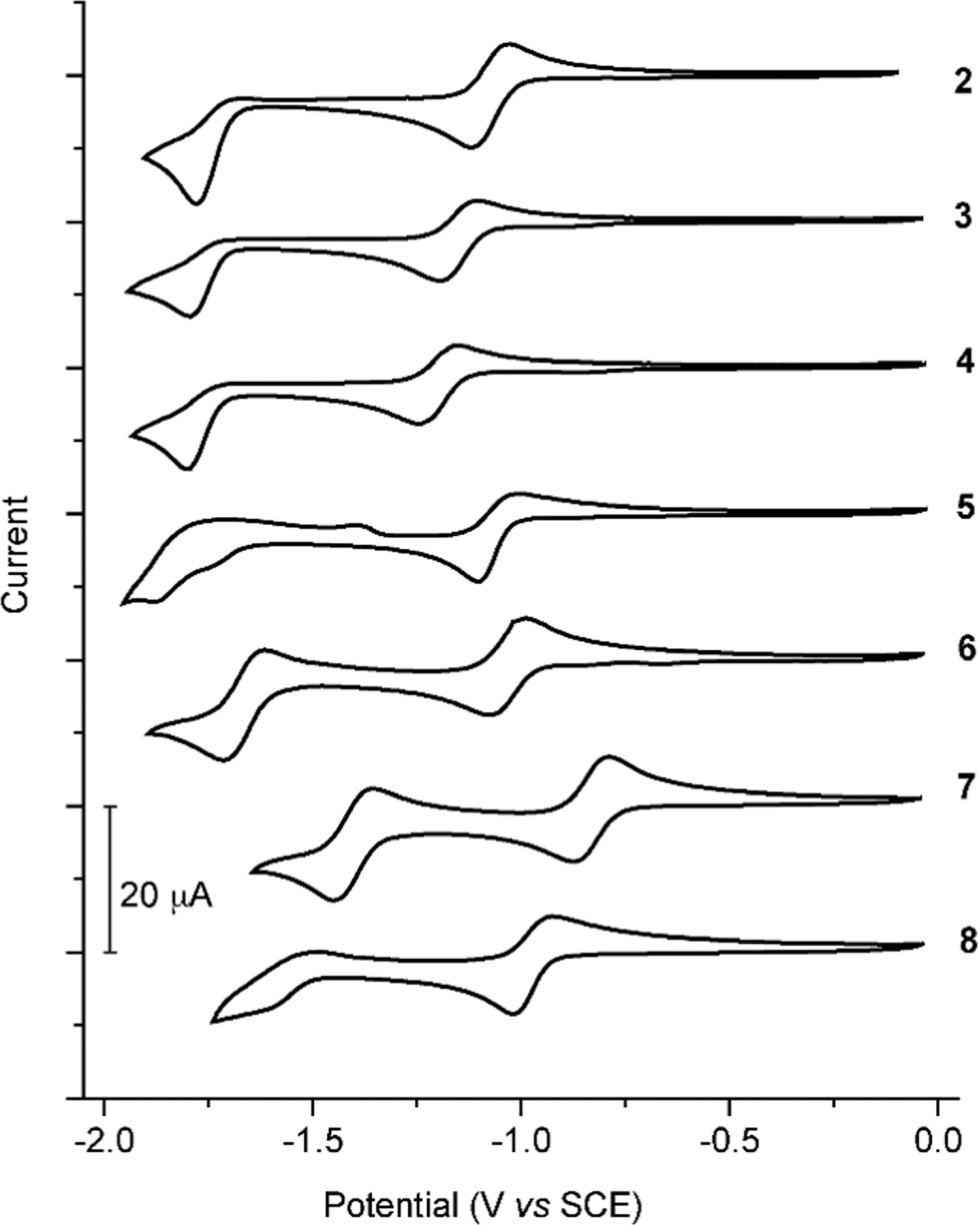
Cyclic voltammograms of complexes **2–8** in MeCN at 100 mV s^–1^.

The cyclic voltammograms of complexes **2**–**8** were registered in MeCN solution and are shown in Figure 6. The potentials of the observed redox processes and estimations of the LUMO energies are listed in Table 4. While no oxidation waves were observed within the electrochemical solvent window, all compounds **2**–**8** show a quasi-reversible wave in the cathodic region with *E*_1/2_ in the range from −0.83 to −1.20 V vs SCE (Δ*E*_p_ = 82–96 mV). A second reduction wave is observed in all cases, except for complex **5**, which is irreversible for complexes **2**–**4** and **8** and quasi-reversible for complexes **6** and **7**.

**Table 4:** Electrochemical Data^a^ and LUMO Energy Estimations^b^ for Complexes **2–8**

complex	*E*_1/2_ (1)^c^	Δ*E*_p_ (1)^d^	*E*_p,c_^e^	*E*_1/2_ (2)^c^	Δ*E*_p_ (2)^d^	*E*_LUMO_
**2**	–1.07	82	–1.78			–3.70
**3**	–1.15	90	–1.79			–3.63
**4**	–1.20	93	–1.80			–3.57
**5**	–1.05	96				–3.68
**6**	–1.03	84		–1.66	99	–3.75
**7**	–0.83	82		–1.40	86	–3.94
**8**	–1.01	95	–1.64			–3.74

a In V vs SCE, registered in a 0.1 M solution of (Bu_4_N)PF_6_ in dry MeCN at 100 mV s^–1^.

b In eV.

c For the first (1) and second (2) quasi-reversible waves.

d In mV.

e Irreversible cathodic peak potential.

The estimated LUMO energies for derivatives with bpy-based diimines increase as electron-donating substituents are introduced on the bpy ligand, resulting in the ordering **2** < **3** < **4**. Among derivatives with phen-based diimines, the extension of the π conjugation in the diimine causes a decrease in the LUMO energy, with the lowest value found for complex **7**. These data are thus consistent with LUMOs that correspond mainly to the lowest π* orbital of the diimine, as shown by the computational results. This is in sharp contrast with the series of complexes [Pt(trz)_2_(C∧N)]^+^, in which the LUMO was found to be composed of the combined lowest π* orbitals of the trz ligands.^53^

The present derivatives constitute a unique series of luminescent Pt(IV) complexes showing quasi-reversible reduction waves. Previously studied complexes of the types *mer/fac*-[Pt(C∧N)_3_]^+^,^39,46^ [Pt(C∧N)_2_(Me)Cl],^41^ [Pt(C∧N)_2_(N∧N)]^2+^,^59,60^ [Pt(trz)(C∧N)Cl_2_],^52^ and [Pt(trz)_2_(C∧N)]^+^^53^ show totally irreversible reductions (Table S2), with the exception of [Pt(trz)_2_(flpy)]^+^ [flpy = cyclometalated 2-(9,9-dimethylfluoren-2-yl)pyridine], which shows a quasi-reversible reduction.^53^ Complexes [Pt(C∧N)_2_(N∧N)]^2+^ have been hypothesized to produce *cis*-[Pt(C∧N)_2_] with loss of the N∧N ligand upon electrochemical reduction.^59,60^ The quasi-reversible wave observed for **2**–**8** indicates that the species formed upon reduction are more stable as compared with most of the previously reported Pt(IV) complexes, probably because it does not lead to reduction of the metal center.

## Conclusions

A series of dicationic, *C*_2_-symmetrical, bis-cyclometalated complexes of the type [Pt(trz)_2_(N∧N)]^2+^ have been obtained by means of chloride abstraction from the precursor [PtCl_2_(trz)_2_] in the presence of bpy- and phen-based aromatic diimines (N∧N). An evaluation of their luminescence in solution and PMMA matrix showed that they can produce phosphorescence, dual fluorescence/phosphorescence, or fluorescence from LC excited states involving the diimine. The observation of phosphorescence is favored for the bpy-based diimines, whereas more extended aromatic systems tend to show fluorescence as a secondary or main radiative decay process, depending on the extension of the aromatic system, as a consequence of the diminished metal orbital contribution to the emissive excited state, which makes intersystem crossing more difficult. A comparison with related complexes of the type [Pt(C∧N)_2_(N∧N)]^2+^ indicates that the replacement of cyclometalated 2-arylpyridines (C∧N) with aryl-NHC ligands leads to higher emission efficiencies from excited states centered on the diimine, particularly in the cases of the ligands with less extended aromatic systems. The {Pt(trz)_2_} substructure is thus demonstrated as suitable for the development of luminescent Pt(IV) complexes bearing an aromatic diimine as the chromophoric ligand. In addition, the introduction of bulky substituents on the diimine, such as the *tert*-butyl groups in **3**, has proven to be a successful strategy to achieve significant emission quantum yields. The electrochemical study on the new complexes has shown that they exhibit an unusually high redox stability, presenting quasi-reversible one-electron reductions involving the lowest π* orbital of the diimine ligand, which contrasts with most of the previously reported luminescent Pt(IV) complexes, which show irreversible reductions.

## Experimental Section

### General Considerations and Instrumentation

All reactions involving silver reagents were carried out in the dark under the N_2_ atmosphere. Synthesis-grade solvents were obtained from commercial sources. The bis-cyclometalated complex [PtCl_2_(trz)_2_] (**1**) was synthesized according to the reported method.^53^ Compound 2,3-diphenylpyrazino[2,3-*f*][1,10]phenanthroline was prepared through a modification of the reported procedure.^68^ All other reagents were obtained from commercial sources. Elemental analyses were determined using a LECO CHNS-932 microanalyzer. Electrospray ionization high-resolution mass spectra (ESI-HRMS) were registered on an Agilent 6220 Accurate-Mass time-of-flight (TOF) LC/MS. NMR spectra were registered on a 600 MHz Bruker Avance spectrometer at room temperature. Chemical shifts are referred to residual signals of nondeuterated solvents and are given in ppm downfield from tetramethylsilane.

#### General Procedure for the Synthesis of [Pt(trz)_2_(N∧N)](OTf)_2_ (**2**–**8**)

A Carius tube was charged with [PtCl_2_(trz)_2_] (**1**) (∼0.1 mmol), AgOTf (2.4 mol equiv), the N∧N ligand (1 mol equiv) and 1,2-dichlorobenzene (2 mL). The mixture was stirred at 120 °C for 18 h under an N_2_ atmosphere. After cooling to room temperature, CH_2_Cl_2_ (5 mL) was added and the mixture was filtered through Celite. The solvent was removed under reduced pressure, and Et_2_O was added dropwise until a precipitate appeared. The solid was collected by filtration, washed with Et_2_O (2 × 4 mL), and vacuum-dried to give **2**–**8**.

#### Data for [Pt(trz)_2_(bpy)](OTf)_2_ (**2**)

White solid, obtained from **1** (70 mg, 0.101 mmol), AgOTf (62 mg, 0.242 mmol), and bpy (16 mg, 0.103 mmol). Yield: 91 mg, 84%. ^1^H NMR (600 MHz, CD_2_Cl_2_): δ 8.84 (d, *J*_HH_ = 8.3 Hz, 2H, CH), 8.41 (td, *J*_HH_ = 8.0, 1.6 Hz, 2H, CH), 7.99 (ddd with satellites, *J*_HH_ = 5.6, 1.6, 0.7 Hz, *J*_PtH_ = 17 Hz, 2H, CH), 7.86 (dd with satellites, *J*_HH_ = 8.0, 1.4 Hz, *J*_PtH_ ∼ 8 Hz, 2H, CH), 7.67 (ddd, *J*_HH_ = 7.7, 5.5, 1.3 Hz, 2H, CH), 7.42 (ddd, *J*_HH_ = 8.0, 7.5, 1.2 Hz, 2H, CH), 7.19 (td, *J*_HH_ = 7.7, 1.4 Hz, 2H, CH), 6.70 (dd with satellites, *J*_HH_ = 7.9, 1.2 Hz, *J*_PtH_ = 47 Hz, 2H, CH), 4.20 (s, 6H, NCH_3_), 2.13 (ddd, *J*_HH_ = 15.1, 11.3, 5.6 Hz, 2H, CH_2_), 1.71–1.64 (m, 2H, CH_2_), 1.22–1.11 (m, 2H, CH_2_), 1.07–0.99 (m, 2H, CH_2_), 0.97–0.90 (m, 4H, CH_2_), 0.74 (t, *J*_HH_ = 7.3 Hz, 6H, CH_3_). ^13^C{^1^H} APT NMR (151 MHz, CD_2_Cl_2_): δ 155.0 (C), 149.5 (CH, *J*_PtC_ ∼ 20 Hz), 145.9 (C, *J*_PtC_ = 60 Hz), 143.3 (C, *J*_PtC_ = 800 Hz), 143.1 (CH), 141.9 (C), 133.7 (CH, *J*_PtC_ ∼ 20 Hz), 131.8 (CH, *J*_PtC_ = 48 Hz), 129.7 (CH, *J*_PtC_ ∼ 18 Hz), 128.7 (CH), 126.9 (CH), 120.0 (C), 117.6 (CH, *J*_PtC_ ∼ 26 Hz), 37.7 (NCH_3_), 32.0 (CH_2_), 24.1 (CH_2_), 22.9 (CH_2_), 13.6 (CH_3_). HRMS (ESI+) *m*/*z*: [M^+^] Calcd for [C_34_H_40_N_8_Pt(CF_3_SO_3_)]^+^ 928.2541; Found 928.2564. HRMS (ESI+) *m*/*z*: [M^2+^] Calcd for [C_34_H_40_N_8_Pt]^2+^ 389.6508; Found 389.6531. Anal. Calcd for C_38_H_40_F_6_N_8_O_6_PtS_2_: C, 42.34; H, 3.74; N, 10.39; S, 5.95. Found: C, 42.26; H, 3.82; N, 10.29; S, 5.93.

#### Data for [Pt(trz)_2_(dbbpy)](OTf)_2_ (**3**)

White solid, obtained from **1** (60 mg, 0.087 mmol), AgOTf (53 mg, 0.208 mmol), and dbbpy (26 mg, 0.096 mmol). Yield: 95 mg, 92%. ^1^H NMR (600 MHz, CD_2_Cl_2_): δ 8.52 (d, *J*_HH_ = 1.6 Hz, 2H, CH), 7.88 (d with satellites, *J*_HH_ = 5.9 Hz, *J*_PtH_ ∼ 17 Hz, 2H, CH), 7.85 (dd, *J*_HH_ = 8.0, 1.4 Hz, 2H, CH), 7.60 (dd, *J*_HH_ = 6.0, 2.0 Hz, 2H, CH), 7.40 (td, *J*_HH_ = 7.8, 1.2 Hz, 2H, CH), 7.17 (td, *J*_HH_ = 7.7, 1.4 Hz, 2H, CH), 6.67 (dd with satellites, *J*_HH_ = 7.8, 1.2 Hz, *J*_PtH_ = 47 Hz, 2H, CH), 4.21 (s, 6H, NCH_3_), 2.26–2.19 (m, 2H, CH_2_), 1.63–1.55 (m, 2H, CH_2_), 1.45 (s, 18H, CH_3_), 1.19–1.03 (m, 4H, CH_2_), 0.99–0.87 (m, 4H, CH_2_), 0.76 (t, *J*_HH_ = 7.3 Hz, 6H, CH_3_). ^13^C{^1^H} APT NMR (151 MHz, CD_2_Cl_2_): δ 168.1 (C), 154.8 (C), 148.9 (*J*_PtC_ = 22 Hz, CH), 145.9 (*J*_PtC_ = 60 Hz, C), 143.4 (*J*_PtC_ ∼ 800 Hz, C), 142.0 (C), 133.6 (*J*_PtC_ = 21 Hz, CH), 131.6 (*J*_PtC_ = 47 Hz, CH), 128.5 (CH), 126.9 (*J*_PtC_ ∼ 17 Hz, CH), 123.2 (CH), 120.4 (*J*_PtC_ = 750 Hz, C), 117.5 (*J*_PtC_ = 27 Hz, CH), 37.7 (NCH_3_), 36.6 (C), 32.0 (CH_2_), 30.2 (CH_3_), 24.0 (CH_2_), 23.0 (CH_2_), 13.7 (CH_3_). HRMS (ESI+) *m*/*z*: [M^+^] Calcd for [C_44_H_56_N_8_Pt(CF_3_SO_3_)]^+^ 1040.3799; Found 1040.3809. HRMS (ESI+) *m*/*z*: [M^2+^] Calcd for [C_44_H_56_N_8_Pt]^2+^ 445.7132; Found 445.7138. Anal. Calcd for C_46_H_56_F_6_N_8_O_6_PtS_2_: C, 46.62; H, 4.74; N, 9.41; S, 5.39. Found: C, 46.56; H, 4.78; N, 9.21; S, 5.34.

#### Data for [Pt(trz)_2_(dMeO-bpy)](OTf)_2_ (**4**)

White solid, obtained from **1** (70 mg, 0.101 mmol), AgOTf (62 mg, 0.242 mmol), and dMeO-bpy (23 mg, 0.103 mmol). Yield: 77 mg, 67%. ^1^H NMR (600 MHz, CD_2_Cl_2_): δ 8.21 (d, *J*_HH_ = 2.6 Hz, 2H, CH), 7.84 (dd, *J*_HH_ = 8.0, 1.4 Hz, 2H, CH), 7.68 (d with satellites, *J*_HH_ = 8.5 Hz, *J*_PtH_ = 17 Hz, 2H, CH), 7.39 (ddd, *J*_HH_ = 8.0, 7.5, 1.2 Hz, 2H, CH), 7.15 (td, *J*_HH_ = 7.7, 1.4 Hz, 2H, CH), 7.06 (dd, *J*_HH_ = 6.5, 2.6 Hz, 2H, CH), 6.67 (dd with satellites, *J*_HH_ = 7.8, 0.9 Hz, *J*_PtH_ = 47 Hz, 2H, CH), 4.21 (s, 6H, CH_3_), 4.14 (s, 6H, CH_3_), 2.23 (ddd, *J*_HH_ = 15.2, 11.2, 5.5 Hz, 2H, CH_2_), 1.81 (ddd, *J*_HH_ = 15.1, 11.0, 5.1 Hz, 2H, CH_2_), 1.25–1.17 (m, 2H, CH_2_), 1.13–1.05 (m, 2H, CH_2_), 1.05–0.96 (m, 4H, CH_2_), 0.77 (t, *J*_HH_ = 7.3 Hz, 6H, CH_3_). ^13^C{^1^H} APT NMR (151 MHz, CD_2_Cl_2_): δ 170.2 (C), 166.8 (C), 149.8 (*J*_PtC_ = 24 Hz, CH), 145.8 (*J*_PtC_ = 61 Hz, C), 143.8 (*J*_PtC_ = 799 Hz, C), 142.0 (C), 133.7 (*J*_PtC_ = 23 Hz, CH), 131.6 (*J*_PtC_ = 48 Hz, CH), 128.5 (CH), 120.6 (*J*_PtC_ = 750 Hz, C), 117.5 (*J*_PtC_ = 26 Hz, CH), 115.8 (*J*_PtC_ = 18 Hz, CH), 112.5 (CH), 58.1 (OCH_3_), 37.6 (NCH_3_), 32.0 (CH_2_), 24.0 (CH_2_), 23.0 (CH_2_), 13.6 (CH_3_). HRMS (ESI+) *m*/*z*: [M^+^] Calcd for [C_38_H_44_N_8_O_2_Pt(CF_3_SO_3_)]^+^ 988.2758; Found 988.2772. HRMS (ESI+) *m*/*z*: [M^2+^] Calcd for [C_38_H_44_N_8_O_2_Pt]^2+^ 419.6612; Found 419.6700. Anal. Calcd for C_40_H_44_F_6_N_8_O_8_PtS_2_: C, 42.22; H, 3.90; N, 9.85; S, 5.64. Found: C, 42.32; H, 3.95; N, 9.88; S, 5.56.

#### Data for [Pt(trz)_2_(phen)](OTf)_2_ (**5**)

White solid, obtained from **1** (60 mg, 0.087 mmol), AgOTf (54 mg, 0.210 mmol), and phen (16 mg, 0.088 mmol). Yield: 64 mg, 67%. ^1^H NMR (600 MHz, CD_2_Cl_2_): δ 8.90 (dd, *J*_HH_ = 8.3, 1.4 Hz, 2H, CH), 8.35 (dd with satellites, *J*_HH_ = 5.2, 1.4 Hz, *J*_PtH_ = 18 Hz, 2H, CH), 8.31 (s, 2H, CH), 8.02 (dd, *J*_HH_ = 8.3, 5.1 Hz, 2H, CH), 7.90 (dd with satellites, *J*_HH_ = 8.1, 1.1 Hz, *J*_PtH_ ∼ 7 Hz, 2H, CH), 7.46 (ddd, *J*_HH_ = 8.0, 7.6, 1.2 Hz, 2H, CH), 7.22 (td with satellites, *J*_HH_ = 7.7, 1.4 Hz, *J*_PtH_ ∼ 7 Hz, 2H, CH), 6.79 (dd with satellites, *J*_HH_ = 7.8, 0.9 Hz, *J*_PtH_ = 48 Hz, 2H, CH), 4.12 (s, 6H, CH_3_), 1.80 (ddd, *J*_HH_ = 15.0, 11.3, 5.7 Hz, 2H, CH_2_), 1.56 (s, 6H, CH_3_), 1.47 (ddd, *J*_HH_ = 15.0, 11.3, 5.1 Hz, 2H, CH_2_), 1.00–0.92 (m, 2H, CH_2_), 0.74–0.66 (m, 2H, CH_2_), 0.64–0.57 (m, 2H, CH_2_), 0.56 (t, *J*_HH_ = 6.7 Hz, 6H, CH_3_), 0.47–0.38 (m, 2H, CH_2_). ^13^C{^1^H} APT NMR (151 MHz, CD_2_Cl_2_): δ 150.6 (*J*_PtC_ = 24 Hz, CH), 146.0 (C), 145.8 (*J*_PtC_ = 60 Hz, C), 143.5 (*J*_PtC_ ∼ 800 Hz, C), 142.2 (C), 141.7 (CH), 133.9 (*J*_PtC_ = 23 Hz, CH), 132.6 (C), 131.6 (*J*_PtC_ = 50 Hz, CH), 129.3 (CH), 128.8 (CH), 127.9 (CH), 119.6 (*J*_PtC_ = 756 Hz, C), 117.7 (*J*_PtC_ = 24 Hz, CH), 37.6 (NCH_3_), 31.8 (CH_2_), 24.0 (CH_2_), 22.8 (CH_2_), 13.6 (CH_3_). HRMS (ESI+) *m*/*z*: [M^+^] Calcd for [C_38_H_40_N_8_Pt(CF_3_SO_3_)]^+^ 952.2544; Found 952.2548. HRMS (ESI+) *m*/*z*: [M^2+^] Calcd for [C_38_H_40_N_8_Pt]^2+^ 401.6506; Found 401.6522. Anal. Calcd for C_40_H_40_F_6_N_8_O_6_PtS_2_: C, 43.60; H, 3.66; N, 10.17; S, 5.82. Found: C, 43.67; H, 3.62; N, 10.07; S, 5.77.

#### Data for [Pt(trz)_2_(bphen)](OTf)_2_ (**6**)

White solid, obtained from **1** (80 mg, 0.115 mmol), AgOTf (71 mg, 0.276 mmol), and bphen (42 mg, 0.127 mmol). Yield: 114 mg, 79%. ^1^H NMR (600 MHz, CD_2_Cl_2_): δ 8.38 (d with satellites, *J*_HH_ = 5.4 Hz, *J*_PtH_ = 18 Hz, 2H, CH), 8.25 (s, 2H, CH), 7.92 (dd, *J*_HH_ = 8.0, 1.4 Hz, 2H, CH), 7.89 (d, *J*_HH_ = 5.4 Hz, 2H, CH), 7.66–7.61 (m, 10H, CH), 7.46 (td, *J*_HH_ = 7.8, 1.2 Hz, 2H, CH), 7.23 (td, *J*_HH_ = 7.7, 1.5 Hz, 2H, CH), 6.82 (dd with satellites, *J*_HH_ = 7.8, 1.2 Hz, *J*_PtH_ = 47 Hz, 2H, CH) 4.17 (s, 6H, NCH_3_), 1.97 (ddd, *J*_HH_ = 15.0, 11.7, 5.4 Hz, 2H, CH_2_), 1.59 (ddd, *J*_HH_ = 15.0, 11.7, 5.0, 2H, CH_2_), 1.10–1.02 (m, 2H, CH_2_), 0.83–0.67 (m, 4H, CH_2_), 0.60–0.51 (m, 2H, CH_2_), 0.58 (t, *J*_HH_ = 7.3 Hz, 6H, CH_3_). ^13^C{^1^H} APT NMR (151 MHz, CD_2_Cl_2_): δ 154.4 (C), 150.0 (CH), 146.7 (C), 145.8 (*J*_PtC_ = 59 Hz, C), 143.6 (*J*_PtC_ = 799 Hz, C), 142.3 (C), 135.0 (C), 133.9 (*J*_PtC_ = 22 Hz, CH), 131.5 (*J*_PtC_ = 49 Hz, CH), 130.7 (CH), 130.1 (CH), 129.7 (CH), 128.6 (CH), 127.8 (*J*_PtC_ = 17 Hz, CH), 127.2 (CH), 120.1 (*J*_PtC_ = 754 Hz, C), 117.6 (*J*_PtC_ = 27 Hz, CH), 37.6 (NCH_3_), 31.8 (CH_2_), 24.1 (CH_2_), 23.0 (CH_2_), 13.6 (CH_3_). HRMS (ESI+) *m*/*z*: [M^+^] Calcd for [C_50_H_48_N_8_Pt(CF_3_SO_3_)]^+^ 1104.3173; Found 1104.3174. HRMS (ESI+) *m*/*z*: [M^2+^] Calcd for [C_50_H_48_N_8_Pt]^2+^ 477.6819; Found 477.6885. Anal. Calcd for C_52_H_48_F_6_N_8_O_6_PtS_2_: C, 49.80; H, 3.86; N, 8.93; S, 5.11. Found: C, 48.84; H, 3.84; N, 8.84; S, 5.14.

#### Data for [Pt(trz)_2_(dppz)](OTf)_2_ (**7**)

White solid, obtained from **1** (48 mg, 0.069 mmol), AgOTf (42 mg, 0.166 mmol), and dppz (20 mg, 0.071 mmol). Yield: 74 mg, 89%. ^1^H NMR (600 MHz, CD_2_Cl_2_): δ 10.06 (dd, *J*_HH_ = 8.3, 1.5 Hz, 2H, CH), 8.54–8.51 (m, 2H, CH), 8.39 (dd with satellites, *J*_HH_ = 5.2, 1.5 Hz, *J*_PtH_ = 18 Hz, 2H, CH), 8.15–8.10 (m, 4H, CH), 7.93 (dd with satellites, *J*_HH_ = 8.0, 1.4 Hz, *J*_PtH_ ∼ 8 Hz, 2H, CH), 7.48 (td, *J*_HH_ = 7.9, 1.2 Hz, 2H, CH), 7.23 (td, *J*_HH_ = 7.7, 1.4 Hz, 2H, CH), 6.81 (dd with satellites, *J*_HH_ = 7.9, 1.1 Hz, *J*_PtH_ = 48 Hz, 2H, CH), 4.14 (s, 6H, NCH_3_), 2.05 (ddd, *J*_HH_ = 15.1, 11.4, 5.0, 2H, CH_2_), 1.48 (ddd, *J*_HH_ = 15.1, 10.7, 5.1, 2H, CH_2_), 1.16–1.07 (m, 2H, CH_2_), 0.90–0.73 (m, 6H, CH_2_), 0.51 (t, *J*_HH_ = 7.1 Hz, 6H, CH_3_). ^13^C{^1^H} APT NMR (151 MHz, CD_2_Cl_2_): δ 151.2 (CH), 148.6 (C), 146.0 (*J*_PtC_ = 61 Hz, C), 143.7 (C), 143.5 (*J*_PtC_ ∼ 800 Hz, C), 142.3 (C), 139.1 (C), 138.9 (CH), 133.9 (*J*_PtH_ = 21 Hz, CH), 133.3 (CH), 132.8 (C), 131.5 (*J*_PtC_ = 49 Hz, CH), 130.3 (CH), 128.9 (CH), 128.8 (CH), 119.7 (*J*_PtC_ = 760 Hz, C), 117.8 (*J*_PtC_ = 27 Hz, CH), 37.7 (NCH_3_), 31.9 (CH_2_), 24.2 (CH_2_), 22.9 (CH_2_), 13.5 (CH_3_). HRMS (ESI+) *m*/*z*: [M^+^] Calcd for [C_44_H_42_N_10_Pt(CF_3_SO_3_)]^+^ 1054.2765; Found 1054.2769. HRMS (ESI+) *m*/*z*: [M^2+^] Calcd for [C_44_H_42_N_10_Pt]^2+^ 452.6617; Found 452.6717. Anal. Calcd for C_46_H_42_F_6_N_10_O_6_PtS_2_: C, 45.89; H, 3.52; N, 11.63; S, 5.33. Found: C, 45.66; H, 3.45; N, 11.75; S, 5.33.

#### Data for [Pt(trz)_2_(dpprzphen)](OTf)_2_ (**8**)

White solid, obtained from **1** (70 mg, 0.101 mmol), AgOTf (64 mg, 0.252 mmol), and dpprzphen (41 mg, 0.105 mmol). Yield: 109 mg, 83%. ^1^H NMR (600 MHz, CD_2_Cl_2_): δ 9.96 (dd, *J*_HH_ = 8.3, 1.4 Hz, 2H, CH), 8.41 (dd with satellites, *J*_HH_ = 5.2, 1.5 Hz, *J*_PtH_ = 17 Hz, 2H, CH), 8.10 (dd, *J*_HH_ = 8.3, 5.2 Hz, 2H, CH), 7.93 (dd with satellites, *J*_HH_ = 8.0, 1.4 Hz, *J*_PtH_ ∼ 7 Hz, 2H, CH), 7.74–7.70 (m, 4H, CH), 7.52–7.41 (m, 8H, CH), 7.26–7.20 (m, 2H, CH), 6.81 (dd with satellites, *J*_HH_ = 7.8, 1.2 Hz, *J*_PtH_ = 48 Hz, 2H, CH), 4.15 (s, 6H, NCH_3_), 2.02 (ddd, *J*_HH_ = 15.1, 11.4, 4.8, 2H, CH_2_), 1.43 (ddd, *J*_HH_ = 15.1, 10.8, 5.0, 2H, CH_2_), 1.14–1.06 (m, 2H, CH_2_), 0.90–0.71 (m, 6H, CH_2_), 0.55 (t, *J*_HH_ = 7.0 Hz, 6H, CH_3_). ^13^C{^1^H} APT NMR (151 MHz, CD_2_Cl_2_): δ 156.0 (C), 151.0 (CH), 147.5 (C), 146.0 (*J*_PtC_ = 62 Hz, C), 143.5 (*J*_PtC_ = 799 Hz, C), 142.3 (C), 138.6 (CH), 138.2 (C), 137.0 (C), 133.9 (CH), 132.0 (C), 131.5 (*J*_PtC_ = 48 Hz, CH), 130.5 (CH), 130.3 (CH), 128.9 (CH), 128.8 (CH), 128.5 (CH), 119.7 (*J*_PtC_ ∼ 760 Hz, C), 117.7 (*J*_PtC_ = 27 Hz, CH), 37.7 (NCH_3_), 31.9 (CH_2_), 24.1 (CH_2_), 22.9 (CH_2_), 13.6 (CH_3_). HRMS (ESI+) *m*/*z*: [M^+^] Calcd for [C_52_H_48_N_10_Pt(CF_3_SO_3_)]^+^ 1156.3235; Found 1156.3238. HRMS (ESI+) *m*/*z*: [M^2+^] Calcd for [C_52_H_48_N_10_Pt]^2+^ 503.6852; Found 503.6944. Anal. Calcd for C_54_H_48_F_6_N_10_O_6_PtS_2_: C, 49.65; H, 3.70; N, 10.72; S, 4.91. Found: C, 49.78; H, 3.69; N, 10.50; S, 4.78.

## Supporting Information Available

Photophysical characterization, X-ray structure determinations, crystallographic data, NMR spectra, electrochemical measurements, excitation and emission spectra, and computational methods and data (PDF)

## References

[ref1] Campagna S.; Puntoriero F.; Nastatsi F.; Bergamini G.; Balzani V. Photochemistry and Photophysics of Coordination Compounds: Ruthenium. Top. Curr. Chem. 2007, 280, 117–214.

[ref2] Demas J. N.; DeGraff B. Design and Applications of Highly Luminescent Transition Metal Complexes. Anal. Chem. 1991, 63, 829–837. 10.1021/ac00017a719.

[ref3] Palion-Gazda J.; Choroba K.; Penkala M.; Rawicka P.; Machura B. Further Insights into the Impact of Ligand-Localized Excited States on the Photophysics of Phenanthroline-Based Rhenium(I) Tricarbonyl Complexes. Inorg. Chem. 2024, 63, 1356–1366. 10.1021/acs.inorgchem.3c03894. 38155540

[ref4] DiLuzio S.; Baumer M.; Guzman R.; Kagalwala H.; Lopato E.; Talledo S.; Kangas J.; Bernhard S. Exploring the Photophysics and Photocatalytic Activity of Heteroleptic Rh(III) Transition-Metal Complexes Using High-Throughput Experimentation. Inorg. Chem. 2024, 63, 14267–14277. 10.1021/acs.inorgchem.4c02420. 39031763 PMC11304382

[ref5] Fernández-Moreira V.; Thorp-Greenwood F. L.; Coogan M. P. Application of d(6) Transition Metal Complexes in Fluorescence Cell Imaging. Chem. Commun. 2010, 46, 186–202. 10.1039/B917757D. 20024327

[ref6] Thorp-Greenwood F. L.; Balasingham R. G.; Coogan M. P. Organometallic Complexes of Transition Metals in Luminescent Cell Imaging Applications. J. Organomet. Chem. 2012, 714, 12–21. 10.1016/j.jorganchem.2012.01.020.

[ref7] Baggaley E.; Gill M. R.; Green N. H.; Turton D.; Sazanovich I. V.; Botchway S. W.; Smythe C.; Haycock J. W.; Weinstein J. A.; Thomas J. A. Dinuclear Ruthenium(II) Complexes as Two-Photon, Time-Resolved Emission Microscopy Probes for Cellular DNA. Angew. Chem., Int. Ed. 2014, 53, 3367–3371. 10.1002/anie.201309427. PMC4298790 24458590

[ref8] Lo K. K. W. Luminescent Rhenium(I) and Iridium(III) Polypyridine Complexes as Biological Probes, Imaging Reagents, and Photocytotoxic Agents. Acc. Chem. Res. 2015, 48, 2985–2995. 10.1021/acs.accounts.5b00211. 26161527

[ref9] Lee L. C.-C.; Leung K.-K.; Lo K. K.-W. Recent Development of Luminescent Rhenium(I) Tricarbonyl Polypyridine Complexes as Cellular Imaging Reagents, Anticancer Drugs, and Antibacterial Agents. Dalton Trans. 2017, 46, 16357–16380. 10.1039/C7DT03465B. 29110007

[ref10] Yip A. M.-H.; Lo K. K.-W. Luminescent Rhenium(I), Ruthenium(II), and Iridium(III) Polypyridine Complexes Containing a Poly(Ethylene Glycol) Pendant or Bioorthogonal Reaction Group as Biological Probes and Photocytotoxic Agents. Coord. Chem. Rev. 2018, 361, 138–163. 10.1016/j.ccr.2018.01.021.

[ref11] Lee L. C.-C.; Lo K. K.-W. Shining New Light on Biological Systems: Luminescent Transition Metal Complexes for Bioimaging and Biosensing Applications. Chem. Rev. 2024, 124, 8825–9014. 10.1021/acs.chemrev.3c00629. 39052606 PMC11328004

[ref12] Prier C. K.; Rankic D. A.; MacMillan D. W. C. Visible Light Photoredox Catalysis with Transition Metal Complexes: Applications in Organic Synthesis. Chem. Rev. 2013, 113, 5322–5363. 10.1021/cr300503r. 23509883 PMC4028850

[ref13] Shon J. H.; Teets T. S. Photocatalysis with Transition Metal Based Photosensitizers. Comments Inorg. Chem. 2020, 40, 53–85. 10.1080/02603594.2019.1694517.

[ref14] Diluzio S.; Connell T. U.; Mdluli V.; Kowalewski J. F.; Bernhard S. Understanding Ir(III) Photocatalyst Structure-Activity Relationships: A Highly Parallelized Study of Light-Driven Metal Reduction Processes. J. Am. Chem. Soc. 2022, 144, 1431–1444. 10.1021/jacs.1c12059. 35025486

[ref15] Pham T. C.; Nguyen V. N.; Choi Y.; Lee S.; Yoon J. Recent Strategies to Develop Innovative Photosensitizers for Enhanced Photodynamic Therapy. Chem. Rev. 2021, 121, 13454–13619. 10.1021/acs.chemrev.1c00381. 34582186

[ref16] Lowry M. S.; Hudson W. R.; Pascal R. A.; Bernhard S. Accelerated Luminophore Discovery through Combinatorial Synthesis. J. Am. Chem. Soc. 2004, 126, 14129–14135. 10.1021/ja047156+. 15506778

[ref17] Evans R. C.; Douglas P.; Winscom C. J. Coordination Complexes Exhibiting Room-Temperature Phosphorescence: Evaluation of Their Suitability as Triplet Emitters in Organic Light Emitting Diodes. Coord. Chem. Rev. 2006, 250, 2093–2126. 10.1016/j.ccr.2006.02.007.

[ref18] Strassner T. Phosphorescent Platinum(II) Complexes with C∧C* Cyclometalated NHC Ligands. Acc. Chem. Res. 2016, 49, 2680–2689. 10.1021/acs.accounts.6b00240. 27993001

[ref19] Mercs L.; Albrecht M. Beyond Catalysis: N-Heterocyclic Carbene Complexes as Components for Medicinal, Luminescent, and Functional Materials Applications. Chem. Soc. Rev. 2010, 39, 1903–1912. 10.1039/b902238b. 20502793

[ref20] Visbal R.; Gimeno M. C. N-Heterocyclic Carbene Metal Complexes: Photoluminescence and Applications. Chem. Soc. Rev. 2014, 43, 3551–3574. 10.1039/C3CS60466G. 24604135

[ref21] Elie M.; Renaud J. L.; Gaillard S. N-Heterocyclic Carbene Transition Metal Complexes in Light Emitting Devices. Polyhedron 2018, 140, 158–168. 10.1016/j.poly.2017.11.045.

[ref22] Stipurin S.; Strassner T. C∧C* Platinum(II) Complexes with Bis(Pyridyl)Borate Ligands: Synthesis, Crystal Structures, and Properties. Organometallics 2024, 43, 1726–1735. 10.1021/acs.organomet.3c00532.

[ref23] Fuertes S.; Chueca A. J.; Arnal L.; Martín A.; Giovanella U.; Botta C.; Sicilia V. Heteroleptic Cycloplatinated N-Heterocyclic Carbene Complexes: A New Approach to Highly Efficient Blue-Light Emitters. Inorg. Chem. 2017, 56, 4829–4839. 10.1021/acs.inorgchem.6b02826. 28387513

[ref24] Sicilia V.; Arnal L.; Chueca A. J.; Fuertes S.; Babaei A.; Muñoz A. M. I.; Sessolo M.; Bolink H. J. Highly Photoluminescent Blue Ionic Platinum-Based Emitters. Inorg. Chem. 2020, 59, 1145–1152. 10.1021/acs.inorgchem.9b02782. 31880921

[ref25] Soellner J.; Strassner T. Phosphorescent Cyclometalated Platinum(II) aNHC Complexes. Chem. - Eur. J. 2018, 24, 15603–15612. 10.1002/chem.201802725. 30216572

[ref26] Di Girolamo A.; Monti F.; Mazzanti A.; Matteucci E.; Armaroli N.; Sambri L.; Baschieri A. 4-Phenyl-1,2,3-Triazoles as Versatile Ligands for Cationic Cyclometalated Iridium(III) Complexes. Inorg. Chem. 2022, 61, 8509–8520. 10.1021/acs.inorgchem.2c00567. 35609179 PMC9490865

[ref27] Amouri H. Luminescent Complexes of Platinum, Iridium, and Coinage Metals Containing N-Heterocyclic Carbene Ligands: Design, Structural Diversity, and Photophysical Properties. Chem. Rev. 2023, 123, 230–270. 10.1021/acs.chemrev.2c00206. 36315851

[ref28] Lee J.; Chen H.-F.; Batagoda T.; Coburn C.; Djurovich P. I.; Thompson M. E.; Forrest S. R. Deep Blue Phosphorescent Organic Light-Emitting Diodes with Very High Brightness and Efficiency. Nat. Mater. 2016, 15, 92–98. 10.1038/nmat4446. 26480228

[ref29] Vivancos Á.; Segarra C.; Albrecht M. Mesoionic and Related Less Heteroatom-Stabilized N-Heterocyclic Carbene Complexes: Synthesis, Catalysis, and Other Applications. Chem. Rev. 2018, 118, 9493–9586. 10.1021/acs.chemrev.8b00148. 30014699

[ref30] Donnelly K. F.; Petronilho A.; Albrecht M. Application of 1,2,3-Triazolylidenes as Versatile NHC-Type Ligands: Synthesis, Properties, and Application in Catalysis and Beyond. Chem. Commun. 2013, 49, 1145–1159. 10.1039/C2CC37881G. 23235474

[ref31] Liu Y.; Kjær K. S.; Fredin L. A.; Chábera P.; Harlang T.; Canton S. E.; Lidin S.; Zhang J.; Lomoth R.; Bergquist K. E.; Persson P.; Wärnmark K.; Sundström V. A Heteroleptic Ferrous Complex with Mesoionic Bis(1,2,3-Triazol-5-Ylidene) Ligands: Taming the MLCT Excited State of Iron(II). Chem. - Eur. J. 2015, 21, 3628–3639. 10.1002/chem.201405184. 25504660

[ref32] Chábera P.; Kjaer K. S.; Prakash O.; Honarfar A.; Liu Y.; Fredin L. A.; Harlang T. C. B.; Lidin S.; Uhlig J.; Sundström V.; Lomoth R.; Persson P.; Wärnmark K. Fe^II^ Hexa N-Heterocyclic Carbene Complex with a 528 ps Metal-To-Ligand Charge-Transfer Excited-State Lifetime. J. Phys. Chem. Lett. 2018, 9, 459–463. 10.1021/acs.jpclett.7b02962. 29298063

[ref33] Sinha N.; Pfund B.; Wegeberg C.; Prescimone A.; Wenger O. S. Cobalt(III) Carbene Complex with an Electronic Excited-State Structure Similar to Cyclometalated Iridium(III) Compounds. J. Am. Chem. Soc. 2022, 144, 9859–9873. 10.1021/jacs.2c02592. 35623627 PMC9490849

[ref34] Ogawa T.; Wenger O. S. Nickel(II) Analogues of Phosphorescent Platinum(II) Complexes with Picosecond Excited-State Decay. Angew. Chem., Int. Ed. 2023, 62, e202312851 10.1002/anie.202312851. 37732725

[ref35] Prakash O.; Lindh L.; Kaul N.; Rosemann N. W.; Losada I. B.; Johnson C.; Chábera P.; Ilic A.; Schwarz J.; Gupta A. K.; Uhlig J.; Ericsson T.; Häggström L.; Huang P.; Bendix J.; Strand D.; Yartsev A.; Lomoth R.; Persson P.; Wärnmark K. Photophysical Integrity of the Iron(III) Scorpionate Framework in Iron(III)-NHC Complexes with Long-Lived ^2^LMCT Excited States. Inorg. Chem. 2022, 61, 17515–17526. 10.1021/acs.inorgchem.2c02410. 36279568 PMC9644379

[ref36] Wenger O. S. Is Iron the New Ruthenium?. Chem. - Eur. J. 2019, 25, 6043–6052. 10.1002/chem.201806148. 30615242

[ref37] Wegeberg C.; Wenger O. S. Luminescent First-Row Transition Metal Complexes. JACS Au 2021, 1, 1860–1876. 10.1021/jacsau.1c00353. 34841405 PMC8611671

[ref38] Garino C.; Salassa L. The Photochemistry of Transition Metal Complexes Using Density Functional Theory. Philos. Trans. R. Soc. A 2013, 371, 20120134 10.1098/rsta.2012.0134. 23776295

[ref39] Juliá F.; Bautista D.; Fernández-Hernández J. M.; González-Herrero P. Homoleptic Tris-Cyclometalated Platinum(IV) Complexes: A New Class of Long-Lived, Highly Efficient ^3^LC Emitters. Chem. Sci. 2014, 5, 1875–1880. 10.1039/C3SC53187B.

[ref40] Juliá F.; Aullón G.; Bautista D.; González-Herrero P. Exploring Excited-State Tunability in Luminescent Tris-Cyclometalated Platinum(IV) Complexes: Synthesis of Heteroleptic Derivatives and Computational Calculations. Chem. - Eur. J. 2014, 20, 17346–17359. 10.1002/chem.201404583. 25353375

[ref41] Juliá F.; Bautista D.; González-Herrero P. Developing Strongly Luminescent Platinum(IV) Complexes: Facile Synthesis of Bis-Cyclometalated Neutral Emitters. Chem. Commun. 2016, 52, 1657–1660. 10.1039/C5CC09566B. 26659999

[ref42] Giménez N.; Lara R.; Moreno M. T.; Lalinde E. Facile Approaches to Phosphorescent Bis(Cyclometalated) Pentafluorophenyl Pt(IV) Complexes: Photophysics and Computational Studies. Chem. - Eur. J. 2017, 23, 5758–5771. 10.1002/chem.201605809. 28272762

[ref43] Giménez N.; Lalinde E.; Lara R.; Moreno M. T. Design of Luminescent, Heteroleptic, Cyclometalated Pt(II) and Pt(IV) Complexes: Photophysics and Effects of the Cyclometalated Ligands. Chem. - Eur. J. 2019, 25, 5514–5526. 10.1002/chem.201806240. 30741462

[ref44] Dikova Y. M.; Yufit D. S.; Williams J. A. G. Platinum(IV) Complexes with Tridentate, NNC -Coordinating Ligands: Synthesis, Structures, and Luminescence. Inorg. Chem. 2023, 62, 1306–1322. 10.1021/acs.inorgchem.2c04116. 36644812 PMC9890496

[ref45] Kang J.; Moon S.-H.; Paek S.; Kang Y. Blue Phosphorescent Platinum(IV) Complex Bearing Bipyridine Ligand for Potential Application in Organic Light-Emitting Diodes (OLEDs). Can. J. Chem. 2023, 101, 133–139. 10.1139/cjc-2022-0093.

[ref46] López-López J. C.; Bautista D.; González-Herrero P. Stereoselective Formation of Facial Tris-Cyclometalated Pt(IV) Complexes: Dual Phosphorescence from Heteroleptic Derivatives. Chem. - Eur. J. 2020, 26, 11307–11315. 10.1002/chem.202001164. 32227518

[ref47] López-López J.-C.; Bautista D.; González-Herrero P. Phosphorescent Biaryl Platinum(IV) Complexes Obtained through Double Metalation of Dibenzoiodolium Ions. Chem. Commun. 2022, 58, 4532–4535. 10.1039/D2CC01018F. 35302577

[ref48] López-López J.-C.; Bautista D.; González-Herrero P. Luminescent Halido(Aryl) Pt(IV) Complexes Obtained via Oxidative Addition of Iodobenzene or Diaryliodonium Salts to Bis-Cyclometalated Pt(II) Precursors. Dalton Trans. 2021, 50, 13294–13305. 10.1039/D1DT02349G. 34499066

[ref49] Juliá F.; García-Legaz M.-D.; Bautista D.; González-Herrero P. Influence of Ancillary Ligands and Isomerism on the Luminescence of Bis-Cyclometalated Platinum(IV) Complexes. Inorg. Chem. 2016, 55, 7647–7660. 10.1021/acs.inorgchem.6b01100. 27438708

[ref50] López-López J. C.; Bautista D.; González-Herrero P. Photoinduced Reductive C–C and C–Heteroatom Couplings from Bis-Cyclometalated Pt(IV) Alkynyl Complexes. Inorg. Chem. 2023, 62, 14411–14421. 10.1021/acs.inorgchem.3c02162. 37616569 PMC10481375

[ref51] Vivancos Á.; Bautista D.; González-Herrero P. Luminescent Platinum(IV) Complexes Bearing Cyclometalated 1,2,3-Triazolylidene and Bi- or Terdentate 2,6-Diarylpyridine Ligands. Chem. - Eur. J. 2019, 25, 6014–6025. 10.1002/chem.201900489. 30807669

[ref52] Vivancos Á.; Jiménez-García A.; Bautista D.; González-Herrero P. Strongly Luminescent Pt(IV) Complexes with a Mesoionic N-Heterocyclic Carbene Ligand: Tuning Their Photophysical Properties. Inorg. Chem. 2021, 60, 7900–7913. 10.1021/acs.inorgchem.1c00410. 33970000 PMC8893362

[ref53] Vivancos Á.; Bautista D.; González-Herrero P. Phosphorescent Tris-Cyclometalated Pt(IV) Complexes with Mesoionic N-Heterocyclic Carbene and 2-Arylpyridine Ligands. Inorg. Chem. 2022, 61, 12033–12042. 10.1021/acs.inorgchem.2c02039. 35860839 PMC9377419

[ref54] Hux J. E.; Puddephatt R. J. Photochemistry of Tetramethyl(2,2′-Bipyridine)Platinum(IV). J. Organomet. Chem. 1988, 346, C31–C34. 10.1016/0022-328X(88)87020-7.

[ref55] Kunkely H.; Vogler A. Electronic Spectra and Photochemistry of Methyl Platinum(IV) Complexes. Coord. Chem. Rev. 1991, 111, 15–25. 10.1016/0010-8545(91)84006-Q.

[ref56] Balashev K. P.; Simon J.; Ford P. C. Photoluminescence Properties of Chloro Amine Complexes of Platinum(IV). Inorg. Chem. 1991, 30, 859–861. 10.1021/ic00004a050.

[ref57] Steel H. L.; Allinson S. L.; Andre J.; Coogan M. P.; Platts J. A. Platinum Trimethyl Bipyridyl Thiolates - New, Tunable, Red- to near IR Emitting Luminophores for Bioimaging Applications. Chem. Commun. 2015, 51, 11441–11444. 10.1039/C5CC04003E. 26086268

[ref58] Mala B.; Murtagh L. E.; Farrow C. M. A.; Akien G. R.; Halcovich N. R.; Allinson S. L.; Platts J. A.; Coogan M. P. Photochemical Oxidation of Pt(IV)Me_3_(1,2-Diimine) Thiolates to Luminescent Pt(IV) Sulfinates. Inorg. Chem. 2021, 60, 7031–7043. 10.1021/acs.inorgchem.0c03553. 33900771

[ref59] Jenkins D. M.; Bernhard S. Synthesis and Characterization of Luminescent Bis-Cyclometalated Platinum(IV) Complexes. Inorg. Chem. 2010, 49, 11297–11308. 10.1021/ic100761f. 21105692

[ref60] Corral-Zorzano A.; Gómez de Segura D.; Lalinde E.; Moreno M. T. Phosphorescent 2-Phenylbenzothiazole Pt(IV) Bis-Cyclometalated Complexes with Phenanthroline-Based Ligands. Dalton Trans. 2023, 52, 6543–6550. 10.1039/D3DT00801K. 37098859

[ref61] Kamecka A.; Kapturkiewicz A.; Wójcik P.; Suwińska K.; Masternak J. Synthesis and Characterization of Platinum(IV) Complexes Containing 1-Phenyl-1H-Pyrazole and α-Diimine Ligands. Eur. J. Inorg. Chem. 2023, 26, e202300438 10.1002/ejic.202300438.

[ref62] Wang W.; Wang P.; Liao X.; Yang B.; Gao C.; Yang J. A Series of Planar Phosphorescent Cyclometalated Platinum(II) Complexes as New Anticancer Theranostic Agents That Induce Oncosis. J. Med. Chem. 2023, 66, 13103–13115. 10.1021/acs.jmedchem.3c01126. 37724909

[ref63] Li G.; Sun L.; Ji L.; Chao H. Ruthenium(II) Complexes with Dppz: From Molecular Photoswitch to Biological Applications. Dalton Trans. 2016, 45, 13261–13276. 10.1039/C6DT01624C. 27426487

[ref64] Zamora A.; Wachter E.; Vera M.; Heidary D. K.; Rodríguez V.; Ortega E.; Fernández-Espín V.; Janiak C.; Glazer E. C.; Barone G.; Ruiz J. Organoplatinum(II) Complexes Self-Assemble and Recognize AT-Rich Duplex DNA Sequences. Inorg. Chem. 2021, 60, 2178–2187. 10.1021/acs.inorgchem.0c02648. 33502194 PMC8456496

[ref65] Lo K. K. W.; Chung C. K.; Zhu N. Nucleic Acid Intercalators and Avidin Probes Derived from Luminescent Cyclometalated Iridium(III)-Dipyridoquinoxaline and -Dipyridophenazine Complexes. Chem. - Eur. J. 2006, 12, 1500–1512. 10.1002/chem.200500885. 16304644

[ref66] Cook N. P.; Torres V.; Jain D.; Martí A. A. Sensing Amyloid-β Aggregation Using Luminescent Dipyridophenazine Ruthenium(II) Complexes. J. Am. Chem. Soc. 2011, 133, 11121–11123. 10.1021/ja204656r. 21714574

[ref67] Juliá F.; González-Herrero P. Spotlight on the Ligand: Luminescent Cyclometalated Pt(IV) Complexes Containing a Fluorenyl Moiety. Dalton Trans. 2016, 45, 10599–10608. 10.1039/C6DT01722C. 27263627

[ref68] Kong M.; Feng X.; Li J.; Hu Z.-B.; Wang J.; Song X.-J.; Jing Z.-Y.; Zhang Y.-Q.; Song Y. Structurally Modulated Single-Ion Magnets of Mononuclear β-Diketone Dysprosium(III) Complexes. Dalton Trans. 2020, 49, 14931–14940. 10.1039/D0DT02864A. 33078800

