# Supplementary material for: Synthesis, Luminescence, and Electrochemistry of Tris-Chelate Platinum(IV) Complexes with Cyclometalated N-Heterocyclic Carbene Ligands and Aromatic Diimines
Source: Inorg Chem. 2024 Dec 19;63(52):24929–39. doi: 10.1021/acs.inorgchem.4c04446 (PMC11688666; doi:10.1021/acs.inorgchem.4c04446)
Supplement: Supplementary file 1 — ic4c04446_si_001.pdf [file ic4c04446_si_001.pdf]

SUPPORTING INFORMATION

Synthesis, Luminescence and Electrochemistry of Tris-Chelate Platinum(IV) Complexes with Cyclometalated N-Heterocyclic Carbene Ligands and Aromatic Diimines

José Serrano-Guarinos,[†] Adrián Jiménez-García,[†] Delia Bautista,[‡] Pablo González-Herrero,[,†] and Ángela Vivancos[*,†]*

[†]Departamento de Química Inorgánica, Facultad de Química, Universidad de Murcia, Campus de Espinardo, 19, 30100 Murcia, Spain.

[‡]Área Científica y Técnica de Investigación, Universidad de Murcia, Campus de Espinardo, 21, 30100 Murcia, Spain.

*E-mail: angela.vivancos@um.es, pgh@um.es.

Contents:

1. Experimental details 2
   1.1. Photophysical characterization 2
   1.2. X-ray structure determinations....................2
2. NMR spectra of new compounds....................4
3. Additional photophysical data 11
4. Electrochemical measurements....................14
5. Computational data 16
   5.1. Complex 2 16
   5.2. Complex 5 21
   5.3. Complex 7 25
6. References....................35

1. Experimental details

1.1. Photophysical characterization

UV-vis absorption spectra were registered on a Perkin-Elmer Lambda 750S spectrophotometer. Excitation and emission spectra were registered on a Jobin Yvon Fluorolog 3-22 spectrofluorometer. Emission spectra were corrected for detector sensitivity. The measurements in solution were carried out in a right-angle configuration using 10 mm quartz fluorescence cells. The emission data in PMMA matrix were measured in a front-face configuration, using quartz slides as sample holders. Phosphorescence lifetimes were determined using an IBH FluoroHub controller in MCS mode and the Fluorolog's FL-1040 phosphorimeter pulsed xenon lamp as excitation source; the estimated uncertainty is ±10% or better. The determination of fluorescence lifetimes was attempted with the TCSPC technique using a pulsed NanoLED source. Emission quantum yields were determined using a Hamamatsu C11347 Absolute PL Quantum Yield Spectrometer; the estimated uncertainty is ±5% or better. Emission lifetimes and quantum yields were determined under rigorous exclusion of atmospheric oxygen.

1.2. X-ray structure determinations

Single crystals suitable for X-ray diffraction were grown by slow liquid-liquid diffusion from CH_2Cl_2/Et_2O (**2**, **4**·1.5CH_2Cl_2, **5**·CH_2Cl_2) or acetone/Et_2O (**7**·Me_2CO·Et_2O). Diffraction data were collected on a Bruker D8 QUEST diffractometer with monochromated Mo-$K\alpha$ radiation performing φ and ω scans. The structures were solved by direct methods and refined anisotropically on F^2 using the program SHELXL-2018 (G. M. Sheldrick, University of Göttingen).[1,2] Numerical details are presented in Table S1. Methyl hydrogens were included as part of rigid idealized methyl groups allowed to rotate but not tip; other hydrogens were included using a riding model. *Special features of refinement:* The structure of **5**·CH_2Cl_2 contains poorly-resolved regions of residual electron density that could not be adequately modelled and therefore were "removed" using the program SQUEEZE, which is part of the PLATON system; the void volume per cell was 1159 Å^3, with a void electron count per cell of 321; this additional solvent was not taken into account when calculating derived parameters such as the formula weight, because the nature of the solvent was uncertain. In **7**·Me_2CO·Et_2O, the acetone molecule and one of the butyl groups are disordered over two positions (ca. 59:41% or 51:49%, respectively).

Table S1. Crystallographic data for **2**, **4**·1.5CH_2Cl_2, **5**·CH_2Cl_2 and **7**·Me_2CO·Et_2O.

	**2**	**4**·1.5CH_2Cl_2	**5**·CH_2Cl_2	**7**·Me_2CO·Et_2O
formula	$C_{38}H_{40}F_6N_8O_6PtS_2$	$C_{41.5}H_{47}Cl_3F_6N_8O_8PtS_2$	$C_{41}H_{42}Cl_2F_6N_8O_6PtS_2$	$C_{53}H_{58}F_6N_{10}O_8PtS_2$
fw	1077.99	1265.43	1186.93	1336.30
T (K)	100(2)	100(2)	100(2)	100(2)
λ	0.71073	0.71073	0.71073	0.71073
cryst syst	Monoclinic	Monoclinic	Monoclinic	Triclinic
space group	P21/n	C2/c	C2/c	P-1
a (Å)	21.6048(14)	46.433(3)	34.696(3)	9.5372(9)
b (Å)	15.1983(10)	9.9564(6)	9.9361(8)	13.0546(13)
c (Å)	26.6836(18)	24.0103(15)	30.104(2)	23.386(2)
α (°)	90	90	90	104.590(4)
β (°)	109.285(2)	118.242(2)	108.929(2)	99.439(4)
γ (°)	90	90	90	95.232(4)
V (Å^3)	8270.1(10)	9778.7(10)	9816.7(13)	2752.7(5)
Z	8	8	8	2
ρ_{calcd} (Mg m^{-3})	1.732	1.719	1.606	1.612
μ (mm^{-1})	3.578	3.201	3.128	2.708
R1^a	0.0225	0.0263	0.0231	0.0333
wR2^b	0.0560	0.0598	0.0513	0.0713

^{a}R1 = $\Sigma||F_o| - |F_c||/\Sigma|F_o|$ for reflections with $I > 2\sigma(I)$. ^{b}wR2 = $[\Sigma[w(F_o^2 - F_c^2)^2/\Sigma[w(F_o^2)^2]]^{0.5}$ for all reflections; $w^{-1} = \sigma^2(F^2) + (aP)^2 + bP$, where $P = (2F_c^2 + F_o^2)/3$ and a and b are constants set by the program.

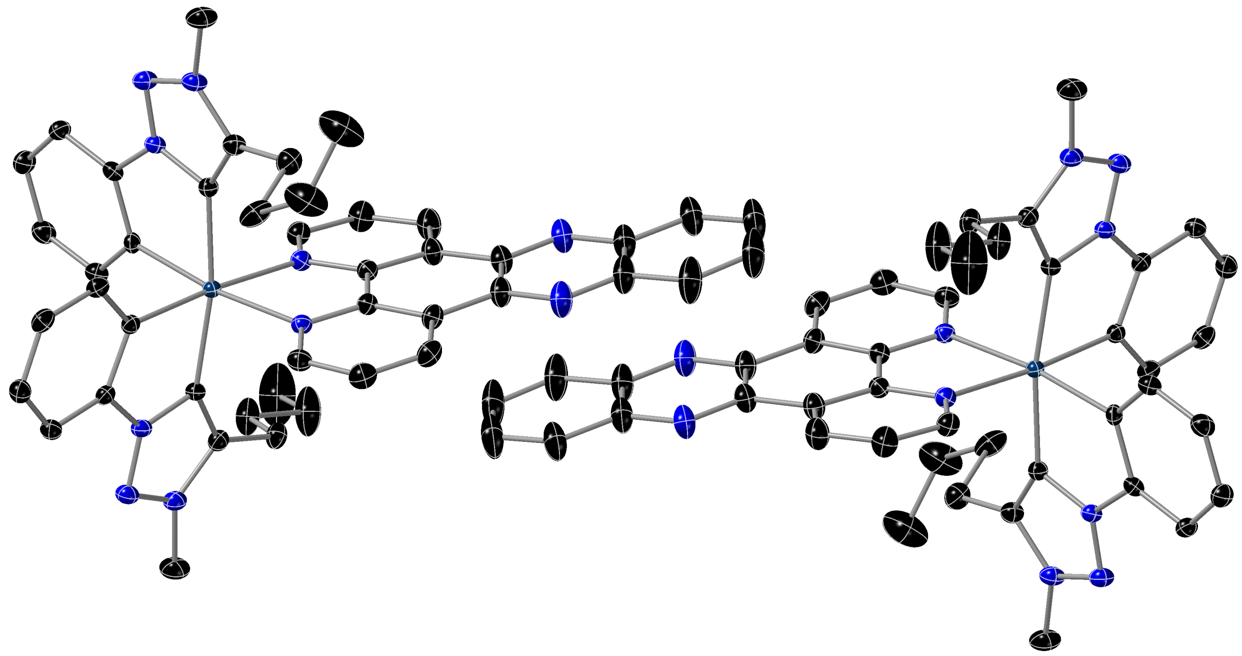

Figure S1. Stacked dimer in the structure of **7**·Me_2CO·Et_2O (thermal ellipsoids at 50% probability). Hydrogen atoms, solvent molecules and anions are omitted.

2. NMR spectra of new compounds

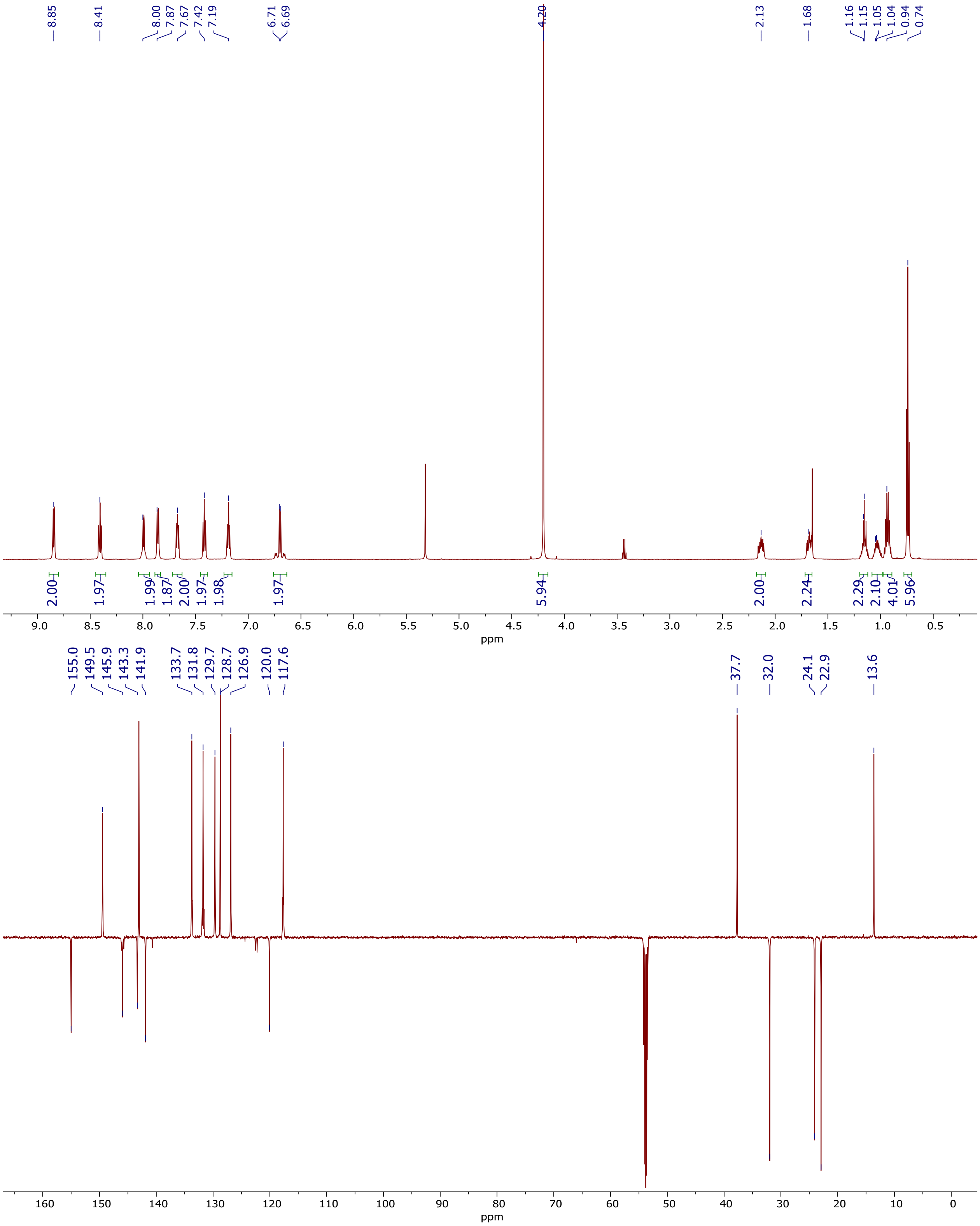

Figure S2. ^{1}H (top) and $^{13}C\{^{1}H\}$ APT (bottom) NMR spectra of complex $[Pt(trz)_2(bpy)](OTf)_2$ (**2**) (CD_2Cl_2, 600 and 151 MHz, respectively).

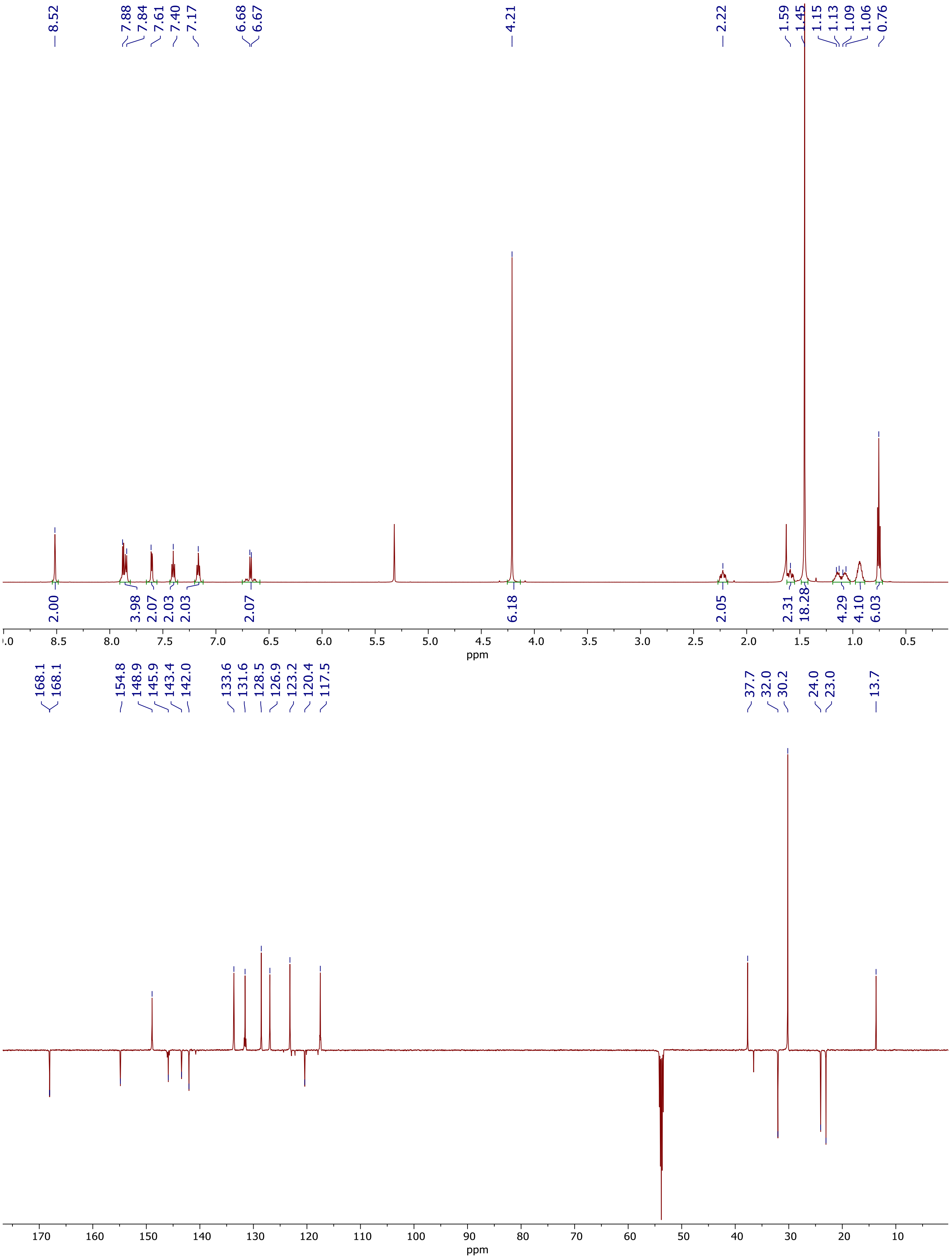

Figure S3. ^{1}H (top) and $^{13}C\{^{1}H\}$ APT (bottom) NMR spectra of complex [Pt(trz)$_2$(dbbpy)](OTf)$_2$ (**3**) (CD_2Cl_2, 600 and 151 MHz, respectively).

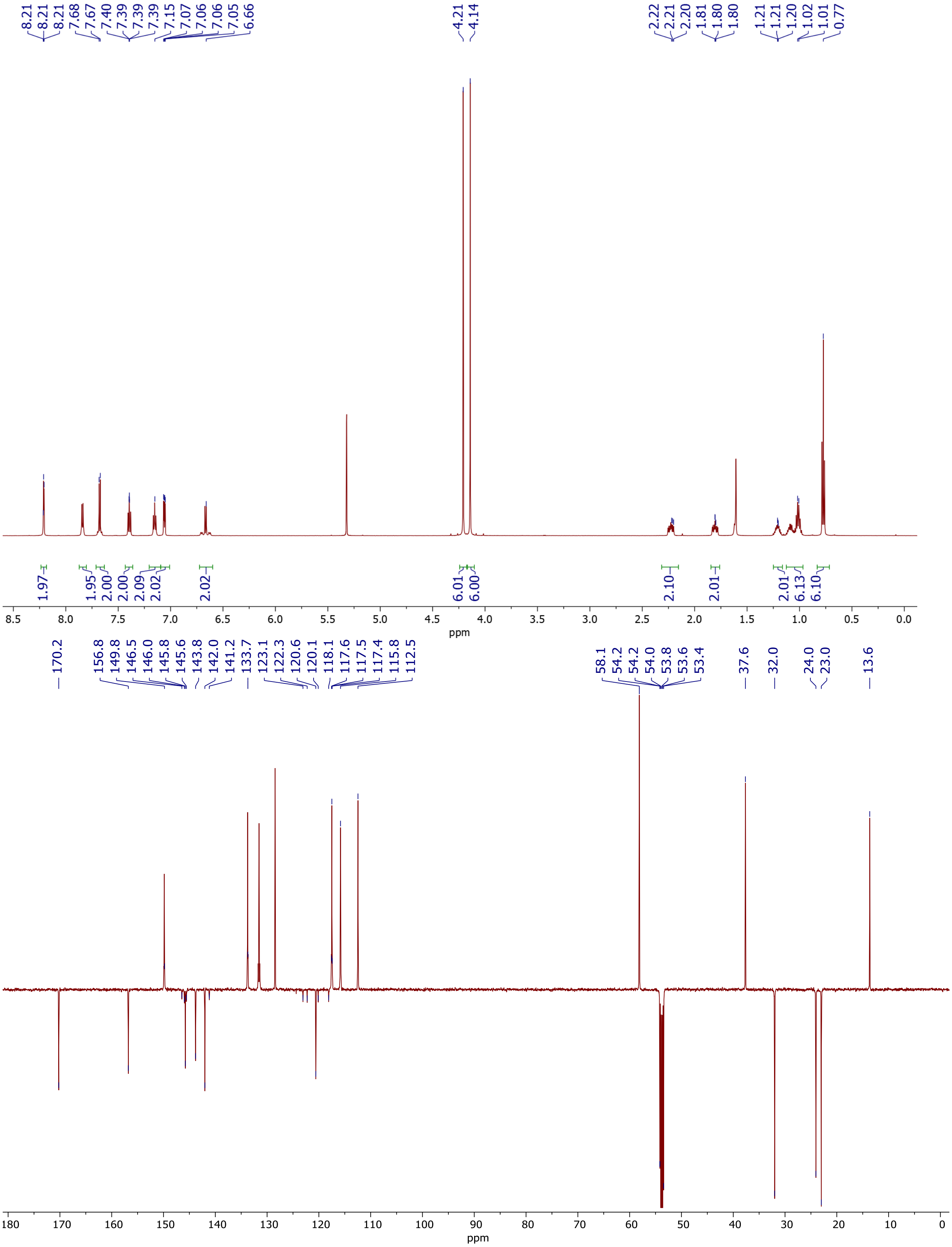

Figure S4. ^{1}H (top) and $^{13}C\{^{1}H\}$ APT (bottom) NMR spectra of complex $[Pt(trz)_2(dMeO\text{-}bpy)](OTf)_2$ (**4**) (CD_2Cl_2, 600 and 151 MHz, respectively).

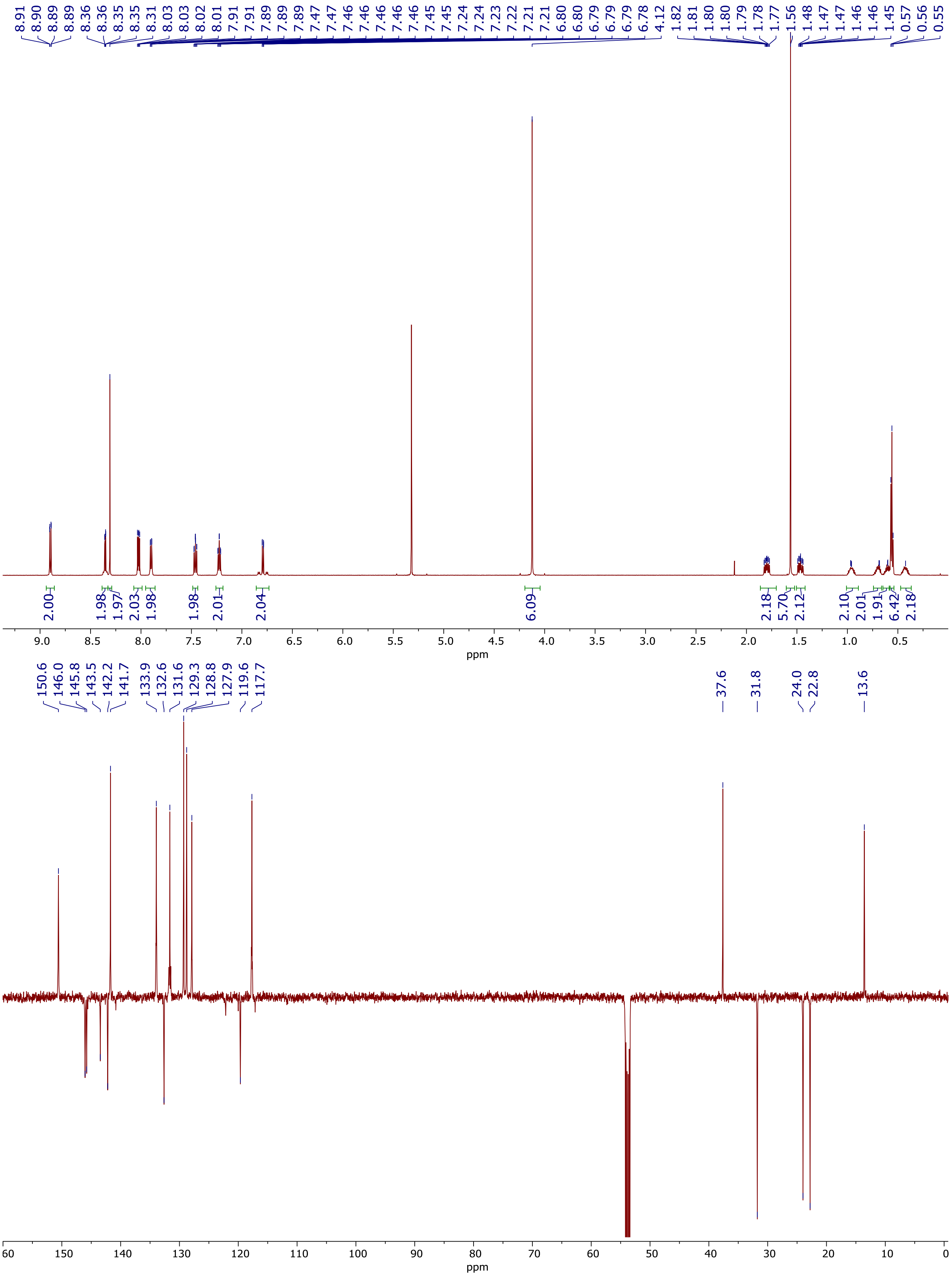

Figure S5. ^{1}H (top) and $^{13}C\{^{1}H\}$ APT (bottom) NMR spectra of complex $[Pt(trz)_2(phen)](OTf)_2$ (**5**) (CD_2Cl_2, 600 and 151 MHz, respectively).

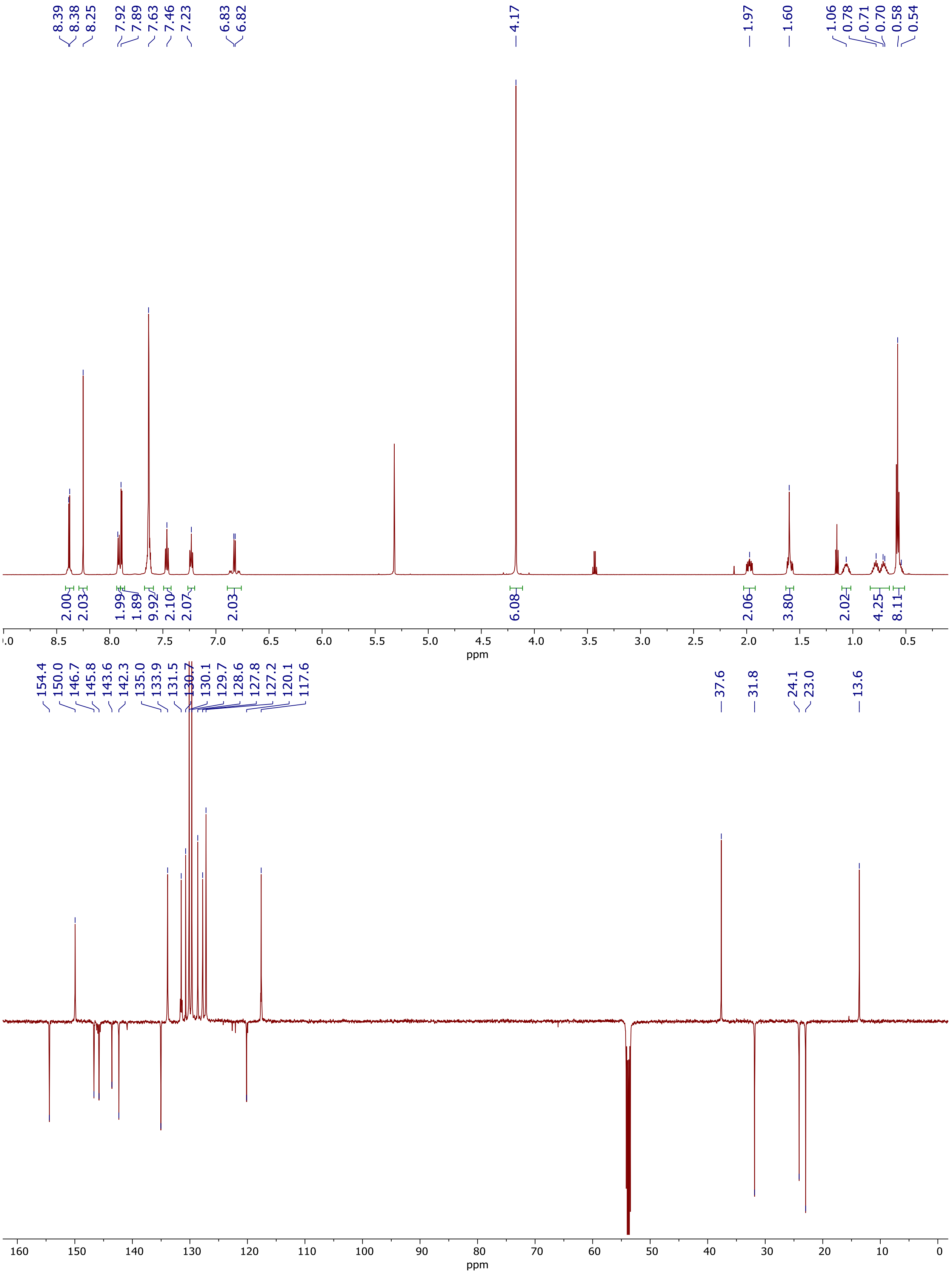

Figure S6. ^{1}H (top) and $^{13}C\{^{1}H\}$ APT (bottom) NMR spectra of complex $[Pt(trz)_2(bphen)](OTf)_2$ (**6**) (CD_2Cl_2, 600 and 151 MHz, respectively).

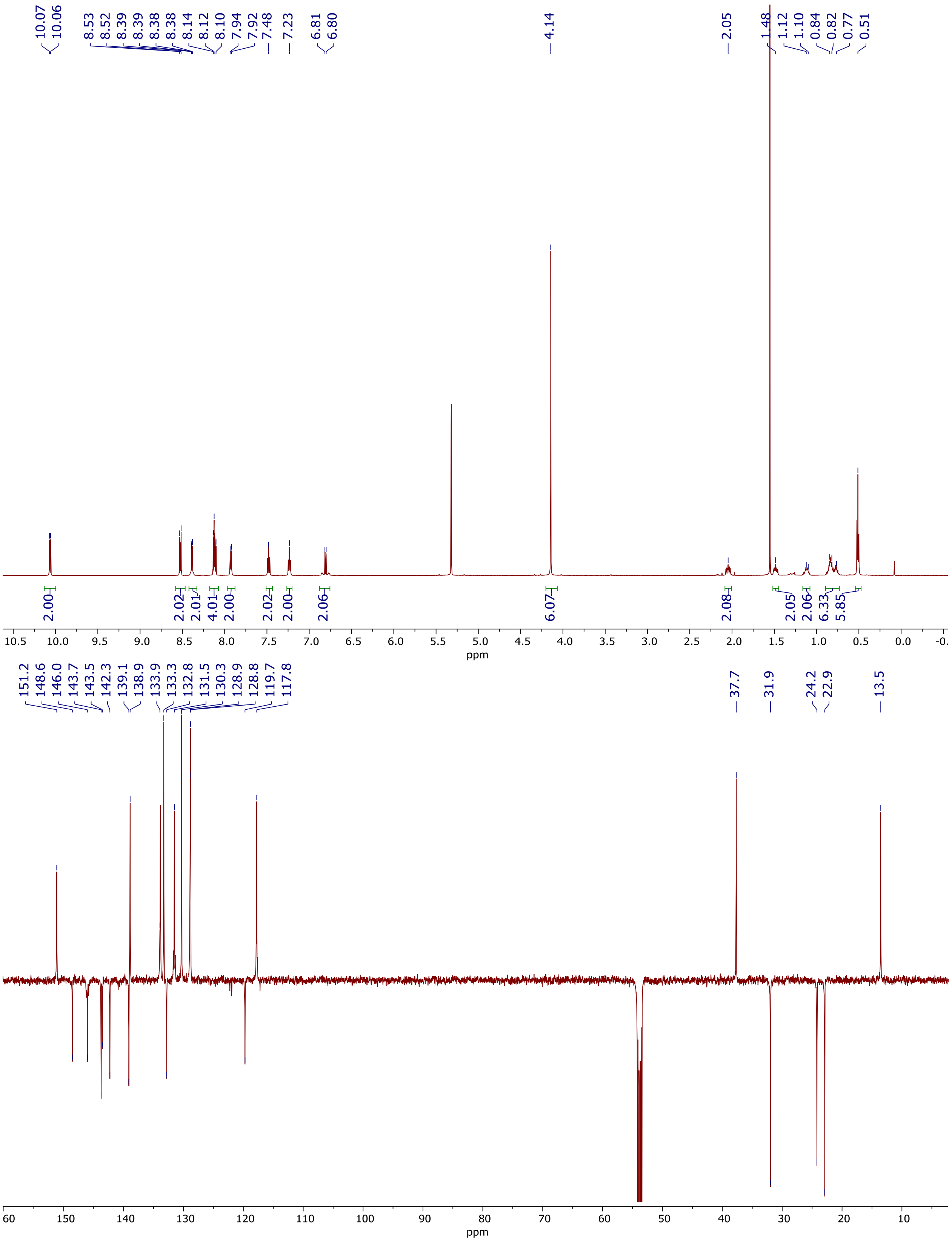

Figure S7. ^{1}H (top) and ^{13}C{^{1}H} APT (bottom) NMR spectra of complex [Pt(trz)$_2$(dppz)](OTf)$_2$ (**7**) (CD_2Cl_2, 600 and 151 MHz, respectively).

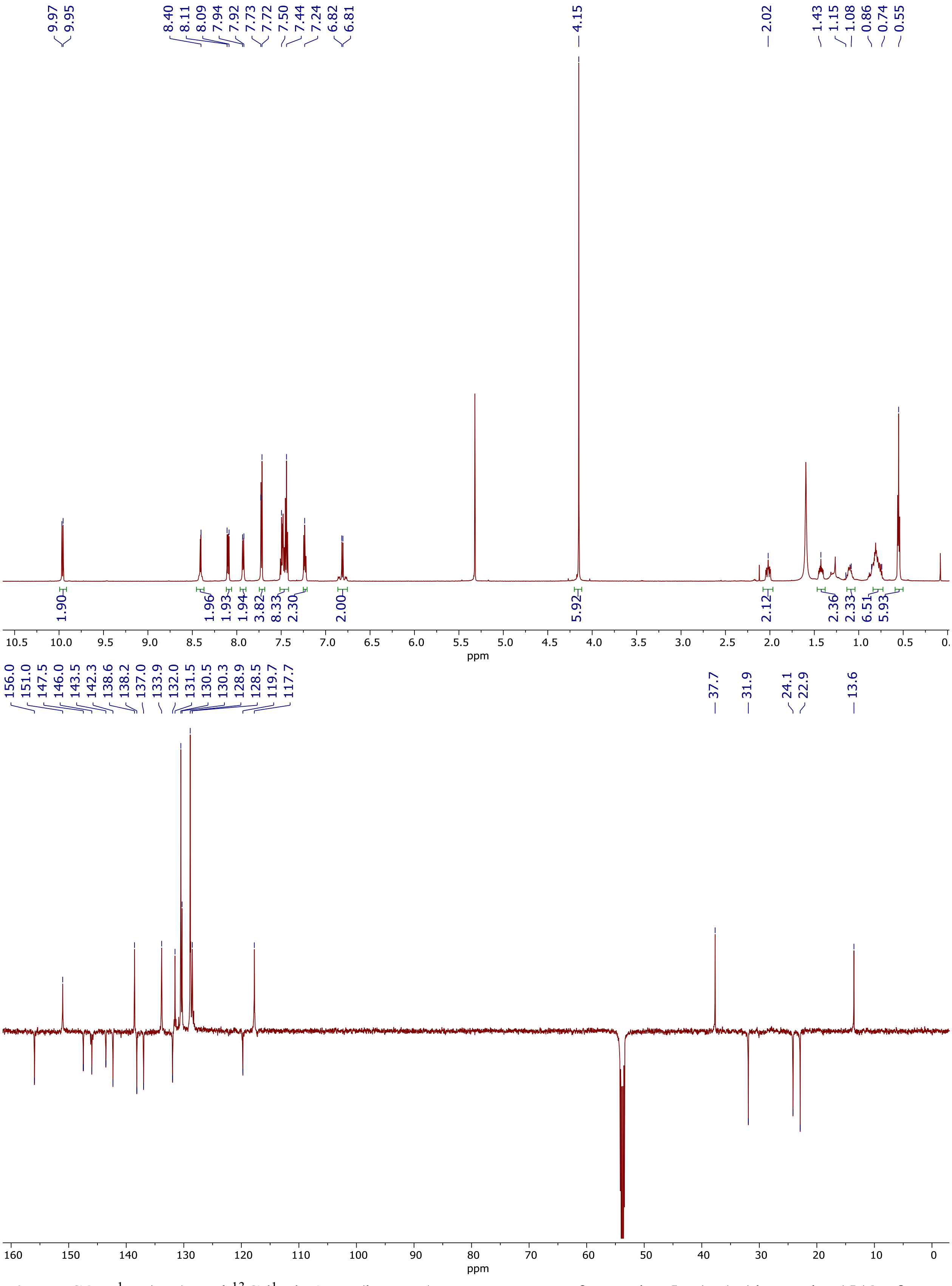

Figure S8. ^{1}H (top) and $^{13}C\{^1H\}$ APT (bottom) NMR spectra of complex [Pt(trz)$_2$(dpprzphen)](OTf)$_2$ (**8**) (CD_2Cl_2, 600 and 151 MHz, respectively).

3. Additional photophysical data

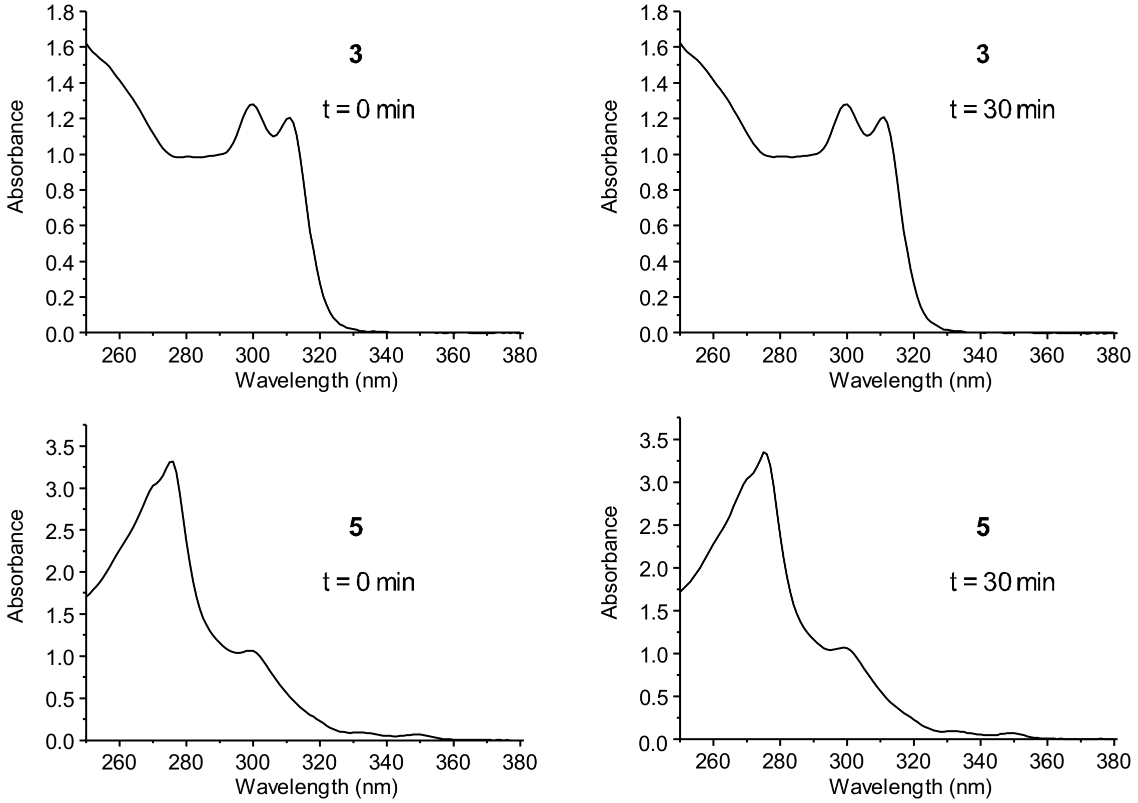

Figure S9. Absorption spectra of complexes **3** and **5** in CH_2Cl_2 solution (ca. 5×10^{-5} M) before and after 30 min of continuous irradiation at 310 or 320 nm, respectively, using the 450 W xenon lamp of the spectrofluorimeter (10 nm bandpass).

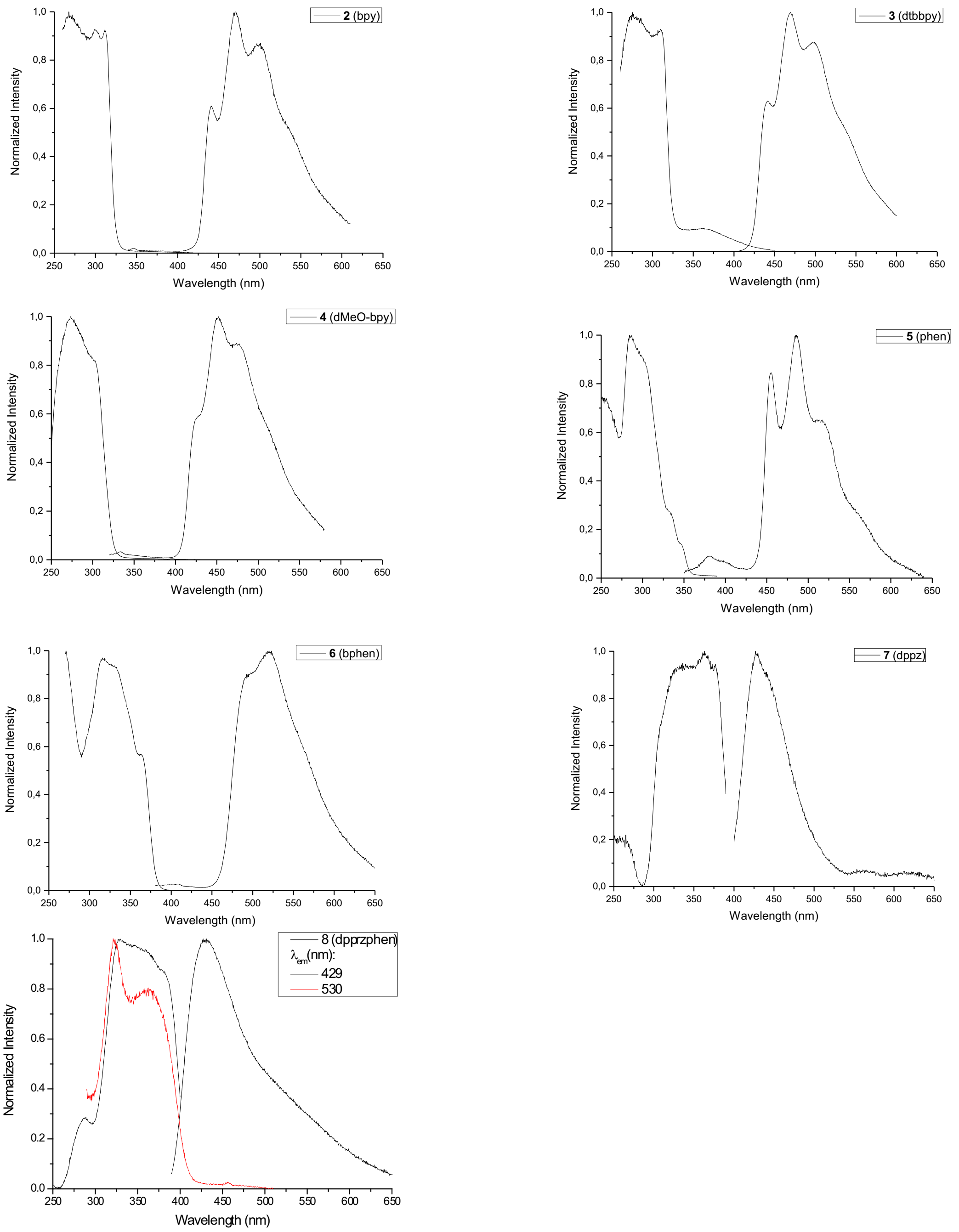

Figure S10. Excitation and emission spectra of complexes **2-8** in CH_2Cl_2 solution (ca. 5×10^{-5} M) at 298 K. The collected λ_{em} for excitation spectra corresponds in all cases to the highest-energy emission peak, with the exception of **8**, for which two emission wavelengths have been monitored.

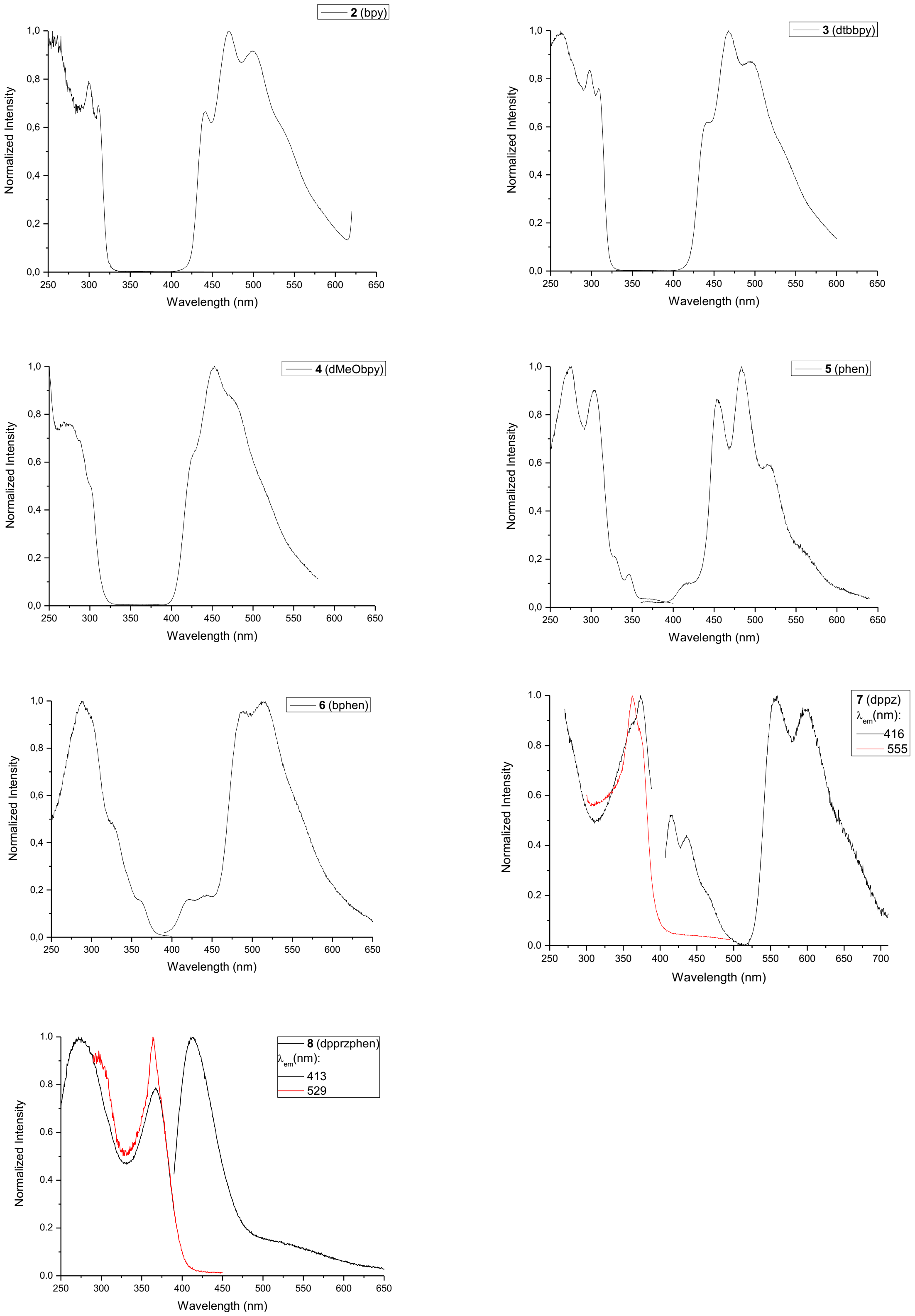

Figure S11. Excitation and emission spectra of complexes **2-8** in PMMA matrices (2 wt%) at 298 K. The collected λ_{em} of the excitation spectra corresponds to the highest-energy peak of the emission, with the exception of **7** and **8**, for which the two emission bands have been monitored.

4. Electrochemical measurements

Cyclic voltammograms were registered at room temperature using an AUTOLAB-100 potentiostat/galvanostat from Echo-Chemie, Utrecht, and a three-electrode electrochemical cell equipped with a glassy carbon working electrode (Metrohm, 2 mm diameter), an Ag/AgCl/3 M KCl electrode reference, and a glassy carbon rod counter electrode. The measurements were carried out using 1 mM solutions of the complexes in extra-dry MeCN (Acros Organics) and 0.1 M $(Bu_4N)PF_6$ as the electrolyte. The solutions were degassed by bubbling argon. The working electrode was polished with alumina slurry (0.05 μm) and rinsed with water and acetone before each experiment. The electrodes were activated electrochemically in the background solution by means of several voltammetric cycles at 1 V s^{-1} between –2.8 V and 2.2 V. The reference electrode was checked against the ferricinium/ferrocene (Fc^+/Fc) redox couple. Potentials are given against the standard calomel electrode (SCE).

The HOMO/LUMO energies were estimated from the onset values of the oxidation and reduction waves, respectively, referenced against the Fc^+/Fc couple (0.40 V vs SCE in MeCN), using a formal potential of 5.1 eV for the Fc^+/Fc couple in the Fermi scale:[3]

$$E_{\mathrm{HOMO}} = -(E_{onset,ox} + 5.1 - 0.4)\ \mathrm{eV};\ E_{\mathrm{LUMO}} = -(E_{onset,red} + 5.1 - 0.4)\ \mathrm{eV}$$

Table S2. Potentials of the first electrochemical reduction of previously reported Pt(IV) complexes with heteroaromatic ligands (see below for ligand name abbreviations).

complex	$E_{p,c}$ [a]	Ref.
mer-[Pt(dfppy)$_3$]OTf	–1.25	4
fac-[Pt(dfppy)$_3$]OTf	–1.60	
mer-[Pt(ppy)$_3$]OTf	–1.50	
fac-[Pt(ppy)$_3$]OTf	–1.80	
mer-[Pt(dfppy)$_2$(ppy)]OTf	–1.32	
fac-[Pt(dfppy)$_2$(ppy)]OTf	–1.64	
mer-[Pt(dfppy)$_2$(thpy)]OTf	–1.29	
mer-[Pt(dfppy)$_2$(piq)]OTf	–1.31	
mer-[Pt(dfppy)$_2$(thpy)]OTf	–1.50	
mer-[Pt(dfppy)$_2$(piq)]OTf	–1.45	
fac-[Pt(tpy)$_3$]BF$_4$	–1.87	7
fac-[Pt(tpy)$_2$(piq)]BF$_4$	–1.45	
fac-[Pt(pq)$_2$(tpy)]BF$_4$	–1.37	
fac-[Pt(pq)$_2$(piq)]BF$_4$	–1.36	
fac-[Pt(thpy)$_2$(tpy)]BF$_4$	–1.72	
fac-[Pt(thpy)$_2$(pic)]BF$_4$	–1.41	
fac-[Pt(piq)$_2$(tpy)]BF$_4$	–1.42	
fac-[Pt(piq)$_3$]BF$_4$	–1.40	
mer-[Pt(flpy)$_3$]OTf	–1.57	9
mer-[Pt(ppy)$_2$(flpy)]OTf	–1.55	
fac-[Pt(flpy)$_3$]OTf	–1.73	
fac-[Pt(ppy)$_2$(flpy)]OTf	–1.78	
[Pt(ppy)$_2$(Me)Cl]	–1.83	11

complex	$E_{p,c}$ [a]	Ref.
(*OC*-6-54)-[Pt(trz)(ppz)Cl$_2$]	–1.67	5
(*OC*-6-54)-[Pt(trz)(dfppy)Cl$_2$]	–1.56	
(*OC*-6-54)-[Pt(trz)(ppy)Cl$_2$]	–1.65	
(*OC*-6-54)-[Pt(trz)(tpy)Cl$_2$]	–1.69	
(*OC*-6-54)-[Pt(trz)(thpy)Cl$_2$]	–1.54	
(*OC*-6-42)-[Pt(trz)(ppz)Cl$_2$]	–1.41	
(*OC*-6-42)-[Pt(trz)(tpy)Cl$_2$]	–1.43	
[Pt(trz)$_2$(dfppy)]OTf	–1.92	6
[Pt(trz)$_2$(ppy)]OTf	–1.99	
[Pt(trz)$_2$(tpy)]OTf	–1.99	
[Pt(trz)$_2$(thpy)]OTf	–1.92	
[Pt(trz)$_2$(flpy)]OTf	–1.88[b]	
[Pt(ppy)$_2$(bpy)](PF$_6$)$_2$	–1.04	8
[Pt(ppy)$_2$(dFbpy)](PF$_6$)$_2$	–0.99	
[Pt(ppy)$_2$(dMeObpy)](PF$_6$)$_2$	–1.12	
[Pt(F-mppy)$_2$(bpy)](PF$_6$)$_2$	–0.98	
[Pt(F-mppy)$_2$(dFbpy)](PF$_6$)$_2$	–0.93	
[Pt(F-mppy)$_2$(dMeObpy)](PF$_6$)$_2$	–1.06	
[Pt(MeO-mppy)$_2$(bpy)](PF$_6$)$_2$	–1.08	
[Pt(MeO-mppy)$_2$(dFbpy)](PF$_6$)$_2$	–1.02	
[Pt(MeO-mppy)$_2$(dMeObpy)](PF$_6$)$_2$	–1.16	
[Pt(pbt)$_2$Cl$_2$]	–1.50[c]	10
[Pt(pbt)$_2$(O$_2$CCF$_3$)$_2$]	–1.49[c]	
[Pt(pbt)$_2$(phen)](CF$_3$CO$_2$)$_2$	–1.46[c]	
[Pt(pbt)$_2$(pyraphen)](CF$_3$CO$_2$)$_2$	–1.50[c]	
[Pt(pbt)$_2$(NH$_2$-phen)](CF$_3$CO$_2$)$_2$	–1.37[c]	

[a] In MeCN solution; cathodic peak potentials of irreversible reductions in V vs. SCE, except where noted. [b] $E_{1/2}$ for quasi-reversible reduction wave. [c] In CH_2Cl_2; reduction onset in V vs Ag/AgCl.

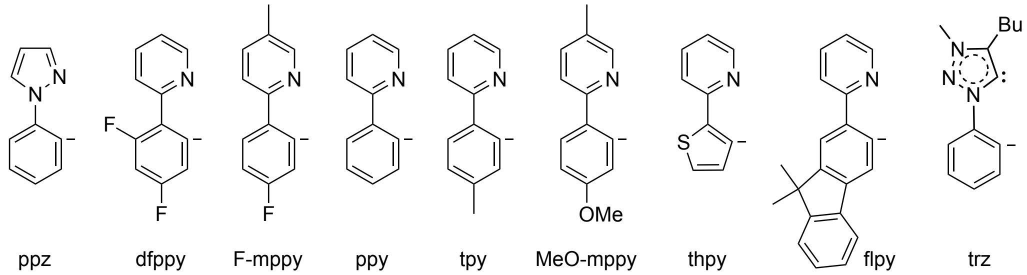

ppz dfppy F-mppy ppy tpy MeO-mppy thpy flpy trz

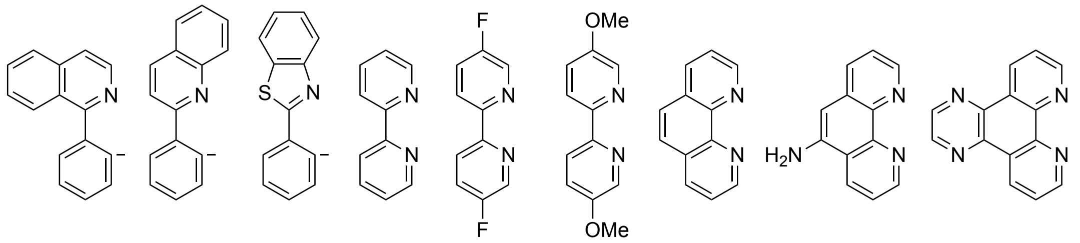

piq pq pbt bpy dFbpy dMeObpy phen NH$_2$-phen pyraphen

5. Computational data

DFT calculations were carried out with Gaussian 16,[12] using the B3LYP functional[13,14] together with the 6-31G**[15,16] basis set for the light atoms and the LANL2DZ[17] basis set and effective core potential for the Pt atom. Optimizations were carried out without symmetry restrictions. Vertical excitation energies were obtained from TDDFT calculations at the ground-state optimized geometries. The solvent effect (CH_2Cl_2) was accounted for in all cases by using the SMD variation of the Polarizable Continuum Model, as implemented in Gaussian.[18] The optimized geometries were confirmed as minima on the potential energy surface by frequency calculations (zero imaginary frequencies).

5.1. Complex 2

Table S3. Fragment contributions (%; from atomic orbital contributions) to the frontier orbitals of **2** in CH_2Cl_2 solution.

energy (a.u.)	number	L1	L2	L3	Pt
-0.062	170 (LUMO+5)	27	27	15	31
-0.063	169 (LUMO+4)	2	0	97	0
-0.069	168 (LUMO+3)	2	1	95	3
-0.079	167 (LUMO+2)	48	49	1	2
-0.084	166 (LUMO+1)	45	43	5	7
–0.105	165 (LUMO)	0	0	98	1
–0.262	164 (HOMO)	40	51	0	8
–0.264	163 (HOMO–1)	55	44	0	0
–0.274	162 (HOMO–2)	33	59	1	6
–0.275	161 (HOMO–3)	62	36	0	0
–0.282	160 (HOMO–4)	2	2	96	0
–0.284	159 (HOMO–5)	44	42	5	9

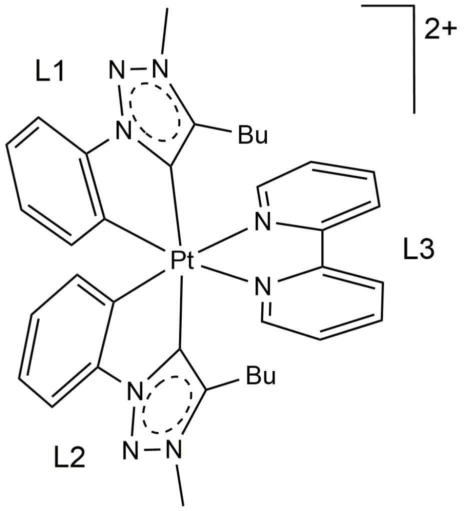

Figure S12. Ligand numbering in complex **2**.

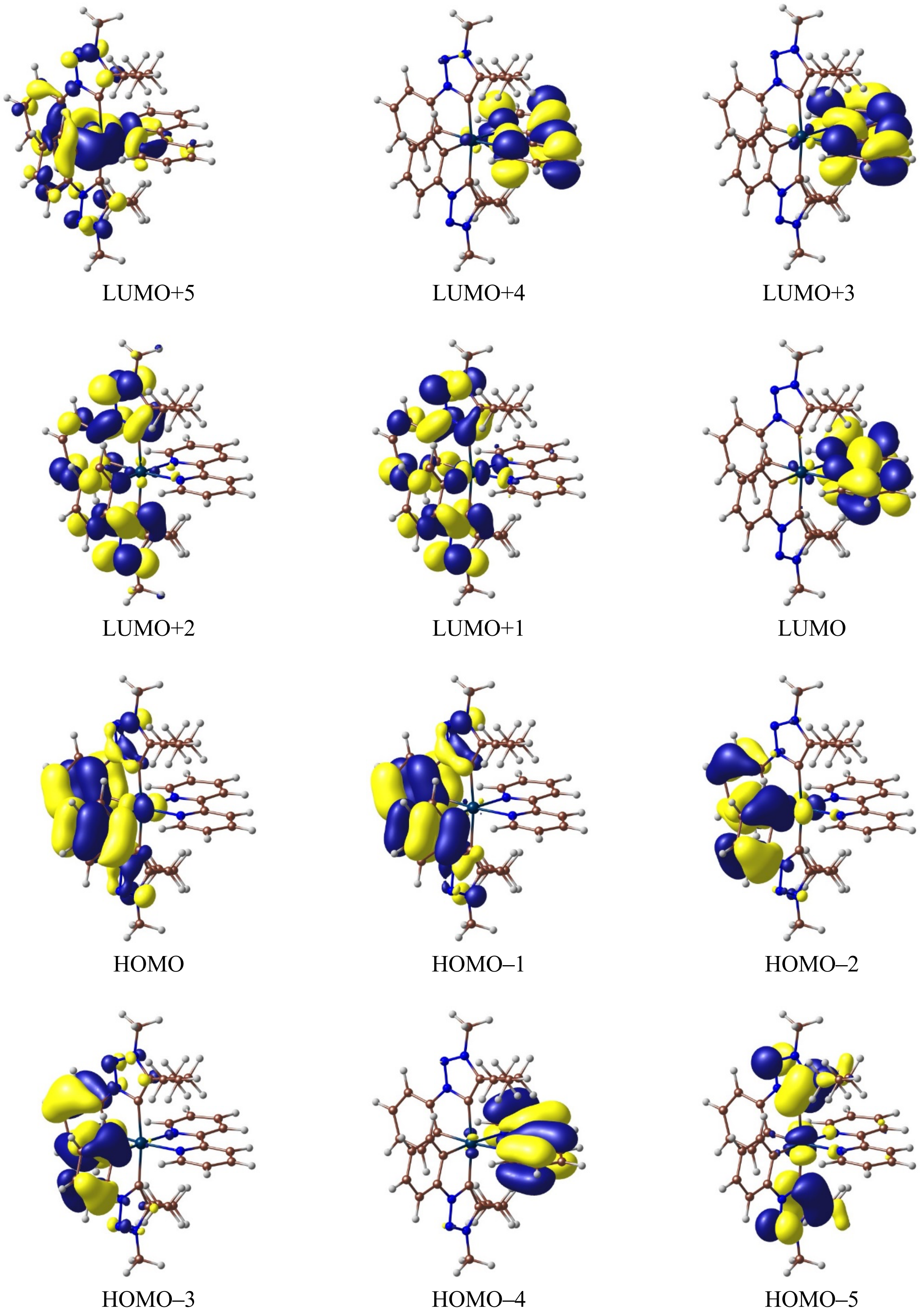

Figure S13. Molecular orbital isosurfaces of **2** (0.03 e bohr^{-3}).

Table S4. Selected vertical singlet excitations of **2** from TDDFT calculations at the ground state geometry in CH_2Cl_2 solution.

State	Monoexcitations	Coefficient (percentage)	ΔE/eV	λ/nm	Oscillator strength	Main character
S_1	164 ->165	0.69882 (98%)	3.7212	333.18	0.0003	LLCT
S_2	163 ->165	0.70424 (99%)	3.8037	325.96	0.0001	LLCT
S_3	162 ->165	0.69189 (96%)	4.0747	304.27	0.0028	LLCT
S_4	161 ->165	0.70281 (99%)	4.12	300.93	0.0003	LLCT
S_5	162 ->166<br>162 ->170<br>164 ->166<br>164 ->170	-0.15283 (5%)<br>-0.10816 (2%)<br>0.61014 (74%)<br>0.21699 (9%)	4.1547	298.42	0.0905	LC(trz)
S_6	159 ->165<br>160 ->165	0.64209 (82%)<br>-0.2703 (15%)	4.2252	293.44	0.0211	LLCT
S_7	158 ->165<br>161 ->166<br>163 ->166<br>164 ->167	0.49 (48%)<br>0.10557 (2%)<br>0.45376 (41%)<br>-0.14726 (4%)	4.2514	291.63	0.0005	LC(trz)
S_8	158 ->165<br>161 ->166<br>163 ->166<br>164 ->167	0.50302 (51%)<br>-0.10289 (2%)<br>-0.45202 (41%)<br>0.10854 (2%)	4.2626	290.87	0.0115	LC(trz)
S_9	159 ->165<br>160 ->165	0.2754 (15%)<br>0.62422 (78%)	4.2975	288.5	0.3482	LC(bpy)
S_{10}	162 ->167<br>163 ->166<br>164 ->167	-0.13363 (4%)<br>0.20659 (9%)<br>0.64433 (83%)	4.3761	283.32	0.0069	LC(trz)
S_{11}	161 ->167<br>162 ->166<br>163 ->167<br>164 ->170	0.16564 (5%)<br>0.1175 (3%)<br>0.62022 (77%)<br>0.21849 (10%)	4.4133	280.93	0.1018	LC(trz)
S_{12}	161 ->167<br>162 ->166<br>163 ->167<br>164 ->168<br>164 ->170	0.14019 (4%)<br>0.49379 (49%)<br>-0.25986 (14%)<br>0.10277 (2%)<br>0.35907 (26%)	4.5049	275.22	0.0395	LC(trz)
S_{13}	162 ->166<br>162 ->170<br>164 ->166<br>164 ->168<br>164 ->170	-0.38055 (29%)<br>-0.21156 (9%)<br>-0.29931 (18%)<br>0.11311 (3%)<br>0.39107 (31%)	4.528	273.82	0.0535	LMCT/<br>LC(trz)
S_{14}	161 ->166<br>162 ->167<br>163 ->170<br>164 ->167	0.5362 (58%)<br>0.22499 (10%)<br>-0.2964 (18%)<br>0.16891 (6%)	4.5401	273.08	0.0516	LC(trz)
S_{15}	161 ->166<br>162 ->167	-0.36568 (27%)<br>0.51952 (54%)	4.6574	266.21	0.0035	LC(trz)

	163 ->166<br>163 ->170	0.10639 (2%)<br>-0.21852 (10%)				
S_{16}	161 ->167<br>162 ->166<br>163 ->169<br>164 ->168	-0.44767 (40%)<br>0.10386 (2%)<br>0.11506 (3%)<br>0.49947 (50%)	4.71	263.24	0.016	LLCT/<br>LC(trz)
S_{17}	161 ->167<br>162 ->166<br>164 ->168<br>164 ->170	0.45471 (41%)<br>-0.1201 (3%)<br>0.4352 (38%)<br>-0.20988 (9%)	4.7188	262.75	0.0313	LC(trz)/<br>LLCT
S_{18}	162 ->167<br>163 ->166<br>163 ->168<br>163 ->170<br>164 ->169	0.33782 (23%)<br>-0.131 (3%)<br>0.38173 (29%)<br>0.39724 (32%)<br>0.14636 (4%)	4.7364	261.77	0.1126	LMCT/<br>LLCT
S_{19}	162 ->167<br>163 ->168<br>163 ->170<br>164 ->169	-0.11855 (3%)<br>0.48423 (47%)<br>-0.38069 (29%)<br>0.26112 (14%)	4.7828	259.23	0.0497	LLCT
S_{20}	159 ->166<br>160 ->166<br>160 ->170	-0.15934 (5%)<br>0.65058 (85%)<br>0.13708 (4%)	4.8237	257.03	0.0217	LLCT

Table S5. Selected vertical triplet excitations of **2** from TDDFT calculations at the ground state geometry in CH_2Cl_2 solution.

State	Monoexcitations	Coefficient (percentage)	ΔE/eV	λ/nm	Main character
T_1	151 ->174<br>156 ->165<br>160 ->165<br>160 ->168	0.10266 (2%)<br>-0.10136 (2%)<br>0.63184 (80%)<br>0.17964 (6%)	3.0789	402.69	LC(bpy)
T_2	161 ->167<br>161 ->172<br>162 ->166<br>162 ->170<br>162 ->173<br>163 ->167<br>164 ->166	0.15425 (5%)<br>-0.10594 (2%)<br>0.15263 (5%)<br>-0.10604 (2%)<br>-0.10444 (2%)<br>-0.38511 (30%)<br>0.43886 (39%)	3.2092	386.34	LC(trz)
T_3	161 ->166<br>161 ->173<br>162 ->167<br>162 ->172<br>163 ->166<br>164 ->167	-0.17217 (6%)<br>0.11031 (2%)<br>-0.14818 (4%)<br>0.10079 (2%)<br>0.41892 (35%)<br>-0.40369 (33%)	3.2182	385.26	LC(trz)
T_{13}	161 ->172<br>162 ->165	-0.16747 (6%)<br>0.22479 (10%)	4.0898	303.16	LMCT

	162 ->170	-0.25490 (13%)			
	162 ->173	-0.14822 (4%)			
	163 ->167	0.24546 (12%)			
	163 ->172	-0.15357 (5%)			
	164 ->170	0.40633 (33%)			

5.2. Complex 5

Table S6. Fragment contributions (%; from atomic orbital contributions) to the frontier orbitals of **5** in CH_2Cl_2 solution.

energy (a.u.)	number	L1	L2	L3	Pt
-0.051	176 (LUMO+5)	0	0	97	2
-0.062	175 (LUMO+4)	28	28	13	32
-0.079	174 (LUMO+3)	49	48	0	2
-0.084	173 (LUMO+2)	45	45	3	7
-0.096	172 (LUMO+1)	0	0	99	0
-0.104	171 (LUMO)	0	0	98	1
-0.262	170 (HOMO)	43	48	0	8
-0.264	169 (HOMO–1)	52	47	0	0
-0.271	168 (HOMO–2)	2	2	95	1
-0.274	167 (HOMO–3)	38	55	1	6
-0.275	166 (HOMO–4)	57	40	2	0
-0.284	165 (HOMO–5)	43	42	5	10

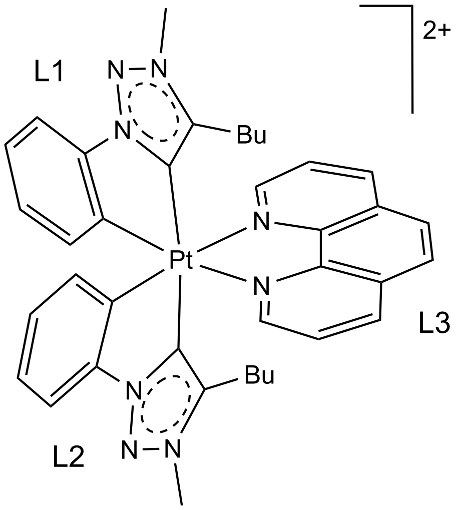

Figure S14. Ligand numbering in complex **5**.

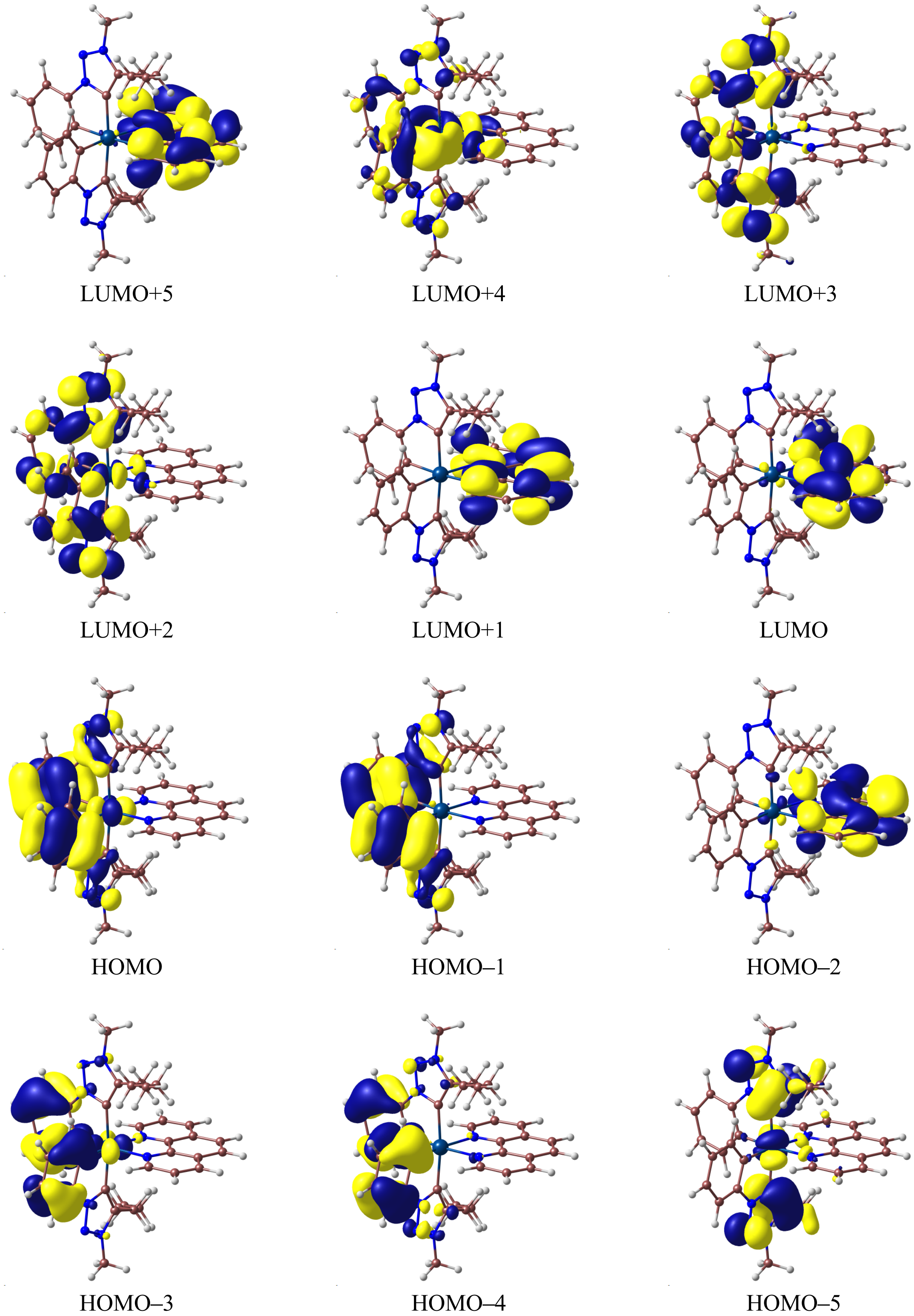

Figure S15. Molecular orbital isosurfaces of **5** (0.03 e bohr^{-3}).

Table S7. Selected vertical singlet excitations of **5** from TDDFT calculations at the ground state geometry in CH_2Cl_2 solution.

State	Monoexcitations	Coefficient (percentage)	ΔE/eV	λ/nm	Oscillator strength	Main character
S_1	170 ->171	0.69747 (97%)	3.7636	329.43	0.0003	LLCT
S_2	168 ->171<br>169 ->171<br>170 ->172	-0.12479 (3%)<br>0.68066 (93%)<br>-0.11842 (3%)	3.8495	322.08	0.0021	LLCT
S_3	163 ->172<br>168 ->171<br>169 ->171	-0.20337 (8%)<br>0.65357 (85%)<br>0.13087 (3%)	3.8883	318.86	0.0411	LC(phen)
S_4	169 ->171<br>170 ->172	0.12779 (3%)<br>0.68999 (95%)	4.0233	308.17	0.0000	LLCT
S_5	169 ->172	0.69619 (97%)	4.0987	302.49	0.0041	LLCT
S_6	167 ->171	0.67394 (91%)	4.1191	301	0.0022	LLCT
S_7	167 ->173<br>167 ->175<br>170 ->173<br>170 ->175	-0.14931 (4%)<br>-0.11401 (3%)<br>0.60761 (74%)<br>0.24151 (12%)	4.1477	298.92	0.0873	LC(trz)
S_8	163 ->171<br>165 ->171<br>167 ->171<br>168 ->172	0.39487 (31%)<br>-0.13352 (4%)<br>0.12273 (3%)<br>0.54382 (59%)	4.148	298.9	0.0139	LC(phen)
S_9	166 ->171<br>167 ->172	0.69306 (96%)<br>0.10551 (2%)	4.1645	297.72	0.0007	LLCT
S_{10}	166 ->173<br>169 ->173<br>169 ->175<br>170 ->174	0.14825 (4%)<br>0.62813 (79%)<br>0.11214 (3%)<br>-0.20006 (8%)	4.2646	290.73	0.0043	LC(trz)
S_{11}	163 ->171<br>165 ->171	0.11594 (3%)<br>0.68816 (95%)	4.2789	289.76	0.0028	LLCT
S_{12}	164 ->171	0.68995 (95%)	4.3097	287.68	0.0018	LLCT
S_{13}	167 ->172<br>169 ->173<br>170 ->174	0.58723 (69%)<br>-0.13585 (4%)<br>-0.32002 (20%)	4.3686	283.81	0.0005	LLCT
S_{14}	167 ->172<br>167 ->174<br>169 ->173<br>170 ->174	0.35321 (25%)<br>-0.10048 (2%)<br>0.17572 (6%)<br>0.5517 (61%)	4.3803	283.05	0.0084	LC(trz)
S_{15}	166 ->172<br>169 ->174<br>170 ->175	0.64255 (83%)<br>0.20999 (9%)<br>0.12459 (3%)	4.4057	281.42	0.0134	LLCT
S_{16}	166 ->172<br>166 ->174<br>167 ->173<br>169 ->174<br>170 ->175	-0.26289 (14%)<br>0.14962 (4%)<br>0.11548 (3%)<br>0.56385 (64%)<br>0.23034 (11%)	4.4256	280.15	0.1053	LC(trz)
S_{17}	166 ->174<br>167 ->173	0.12796 (3%)<br>0.39884 (32%)	4.5041	275.27	0.0148	LC(trz)/LMCT

	169 ->174<br>170 ->173<br>170 ->175	-0.30931 (19%)<br>-0.11963 (3%)<br>0.43733 (38%)				
S_{18}	165 ->173<br>167 ->173<br>167 ->175<br>170 ->173<br>170 ->175	-0.10193 (2%)<br>0.46995 (44%)<br>0.21787 (9%)<br>0.29164 (17%)<br>-0.30442 (19%)	4.5227	274.14	0.15	LC(trz)
S_{19}	165 ->172<br>166 ->173<br>167 ->174<br>168 ->173<br>169 ->175<br>170 ->174	0.13555 (4%)<br>0.46964 (44%)<br>0.1968 (8%)<br>0.2889 (17%)<br>-0.27833 (15%)<br>0.1576 (5%)	4.5385	273.18	0.0442	LC(trz)
S_{20}	165 ->172	0.68652 (94%)	4.5434	272.89	0.0043	LLCT

Table S8. Selected vertical triplet excitations of **5** from TDDFT calculations at the ground state geometry in CH_2Cl_2 solution.

State	Monoexcitations	Coefficient (percentage)	ΔE/eV	λ/nm	Main character
T_1	159 ->171<br>163 ->171<br>163 ->176<br>168 ->172	-0.10237 (2%)<br>0.27234 (15%)<br>0.10559 (2%)<br>0.60133 (72%)	2.8702	431.97	LC(phen)
T_2	159 ->172<br>168 ->171	-0.11224 (3%)<br>0.67513 (91%)	3.1964	387.88	LC(phen)
T_3	166 ->174<br>166 ->178<br>167 ->173<br>167 ->175<br>167 ->180<br>169 ->174<br>170 ->173	0.14878 (4%)<br>-0.10468 (2%)<br>0.15546 (5%)<br>-0.10763 (2%)<br>-0.10434 (2%)<br>-0.38569 (30%)<br>0.44131 (39%)	3.2101	386.23	LC(trz)
T_{11}	166 ->174<br>166 ->181<br>167 ->173<br>170 ->173<br>170 ->175	0.28917 (17%)<br>-0.10846 (2%)<br>0.46618 (43%)<br>-0.26026 (14%)<br>-0.23572 (11%)	3.9623	312.91	LC(trz)/LMCT
T_{18}	166 ->173<br>166 ->175<br>166 ->180<br>167 ->174<br>167 ->177<br>167 ->178<br>169 ->175<br>169 ->180<br>170 ->174<br>170 ->177<br>170 ->178	0.15228 (5%)<br>0.20392 (8%)<br>0.20704 (9%)<br>0.1238 (3%)<br>-0.10771 (2%)<br>0.2051 (8%)<br>0.33235 (22%)<br>0.16698 (6%)<br>0.14978 (4%)<br>0.1394 (4%)<br>-0.22309 (10%)	4.1766	296.85	LMCT

5.3. Complex 7

Table S9. Fragment contributions (%; from atomic orbital contributions) to the frontier orbitals of **7** in CH_2Cl_2 solution.

energy (a.u.)	number	L1	L2	L3	Pt
–0.063	202 (LUMO+5)	29	28	12	31
–0.080	201 (LUMO+4)	48	48	1	2
–0.084	200 (LUMO+3)	45	44	3	8
–0.093	199 (LUMO+2)	0	0	99	0
–0.105	198 (LUMO+1)	0	0	98	1
–0.117	197 (LUMO)	0	0	100	0
–0.258	196 (HOMO)	0	0	100	0
–0.263	195 (HOMO–1)	42	49	0	8
–0.265	194 (HOMO–2)	53	45	0	0
–0.271	193 (HOMO–3)	0	0	98	0
–0.274	192 (HOMO–4)	35	58	1	6
–0.275	191 (HOMO–5)	60	38	1	0

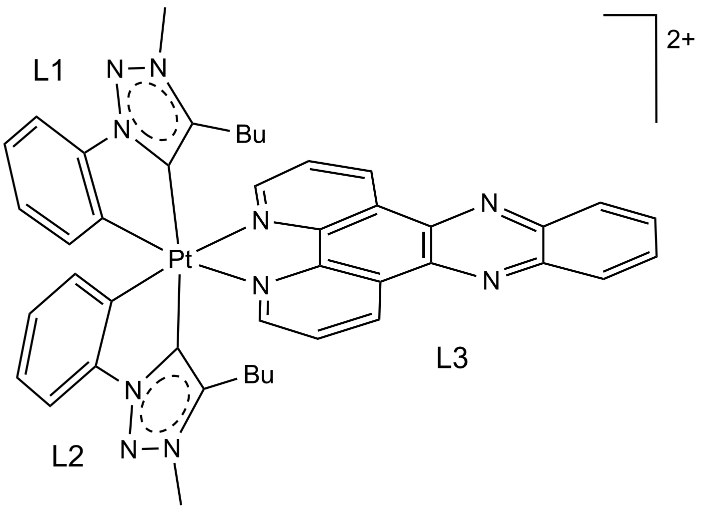

Figure S16. Ligand numbering in complex **7**.

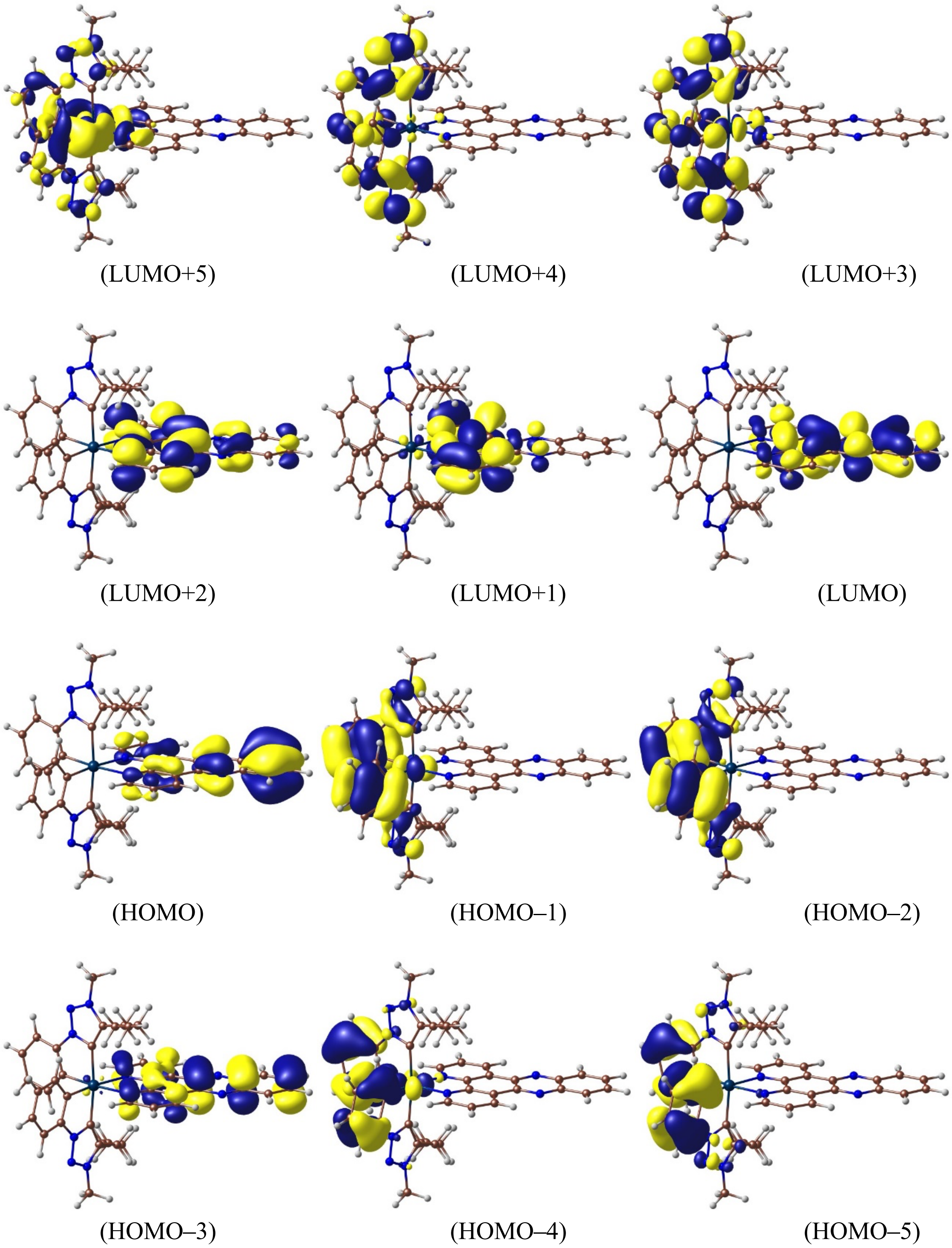

Figure S17. Molecular orbital isosurfaces of **7** (0.03 e bohr^{-3}).

Table S10. Selected vertical singlet excitations of **7** from TDDFT calculations at the ground state geometry in CH_2Cl_2 solution.

State	Monoexcitations	Coefficient (percentage)	ΔE/eV	λ/nm	Oscillator strength	Main character
S_1	190 ->197<br>190 ->198<br>196 ->197	0.67223 (90%)<br>0.13209 (3%)<br>0.15168 (5%)	3.2972	376.03	0.0027	LC(dppz)
S_2	190 ->197<br>196 ->197	-0.15038(5%)<br>0.67737(92%)	3.3029	375.38	0.0228	LC(dppz)
S_3	195 ->197<br>195 ->198	0.67771(92%)<br>-0.18973(7%)	3.5682	347.47	0.0002	LLCT
S_4	193 ->197<br>196 ->199	0.60215(73%)<br>0.33972(23%)	3.5894	345.41	0.2348	LC(dppz)
S_5	194 ->197<br>194 ->198	0.68134(93%)<br>-0.15705(5%)	3.6406	340.56	0.0012	LLCT
S_6	196 ->198	0.69999(98%)	3.6611	338.66	0.0227	LC(dppz)
S_7	195 ->197<br>195 ->198	0.19255(7%)<br>0.66869(89%)	3.7716	328.73	0.0009	LLCT
S_8	194 ->197<br>194 ->198	0.1555(5%)<br>0.67959(92%)	3.8502	322.02	0	LLCT
S_9	192 ->197<br>192 ->198	0.68395(94%)<br>-0.15252(5%)	3.9098	317.11	0	LLCT
S_{10}	191 ->197<br>191 ->198	0.68286(93%)<br>-0.13754(4%)	3.945	314.28	0.0016	LLCT
S_{11}	193 ->198<br>196 ->199	0.67065(90%)<br>0.13142(3%)	3.9506	313.84	0.0321	LC(dppz)
S_{12}	190 ->197<br>190 ->198	-0.14088(4%)<br>0.68839(95%)	4.0239	308.12	0.0001	LC(dppz)
S_{13}	187 ->197<br>189 ->197<br>189 ->198<br>192 ->198	-0.14068(4%)<br>0.6404(82%)<br>-0.22592(10%)<br>0.10553(2%)	4.0842	303.57	0.0013	LLCT
S_{14}	188 ->197<br>188 ->198<br>192 ->198<br>193 ->197<br>193 ->198<br>196 ->199	0.59144(70%)<br>-0.20495(8%)<br>-0.1115(2%)<br>-0.13557(4%)<br>-0.10065(2%)<br>0.23879(11%)	4.1193	300.98	0.2097	LLCT
S_{15}	188 ->197<br>189 ->197<br>192 ->197<br>192 ->198<br>195 ->200	0.10929(2%)<br>-0.11804(3%)<br>0.14027(4%)<br>0.63074(80%)<br>-0.15728(5%)	4.1201	300.93	0.0119	LLCT
S_{16}	190 ->199<br>195 ->199	0.61109(75%)<br>-0.30126(18%)	4.139	299.55	0.0073	LC(dppz)
S_{17}	187 ->197<br>192 ->198	-0.35888(26%)<br>0.13248(4%)	4.1403	299.46	0.0712	LC(trz)

	192 ->200<br>193 ->199<br>195 ->200<br>195 ->202	0.1197(3%)<br>-0.12254(3%)<br>0.47486(45%)<br>0.20228(8%)				
S_{18}	187 ->197<br>192 ->198<br>193 ->199<br>195 ->200<br>195 ->202	0.50481(51%)<br>0.13299(4%)<br>0.19166(7%)<br>0.33891(23%)<br>0.13942(4%)	4.1421	299.32	0.0647	LC(dppz)
S_{19}	187 ->197<br>188 ->197<br>191 ->198<br>193 ->197<br>193 ->198<br>195 ->199<br>196 ->199	0.14621(4%)<br>-0.25946(13%)<br>-0.22533(10%)<br>-0.25727(13%)<br>-0.10597(2%)<br>0.16695(6%)<br>0.46502(43%)	4.1445	299.16	0.8402	LC(dppz)
S_{20}	190 ->199<br>191 ->198<br>193 ->197<br>195 ->199<br>196 ->199	0.28753 (17%)<br>-0.23103(11%)<br>0.11612(3%)<br>0.51918(54%)<br>-0.22075(10%)	4.1549	298.41	0.1808	LLCT

Table S11. Selected vertical triplet excitations of **7** from TDDFT calculations at the ground state geometry in CH_2Cl_2 solution.

State	Monoexcitations	Coefficient (percentage)	ΔE/eV	λ/nm	Main character
T_1	193 ->199<br>196 ->197<br>196 ->198<br>196 ->204	-0.13642 (4%)<br>0.64805 (84%)<br>0.13845 (4%)<br>0.11136 (2%)	2.2673	546.85	LC(dppz)
T_2	190 ->197<br>190 ->198<br>190 ->210	0.6645 (88%)<br>0.17812 (6%)<br>0.10446 (2%)	2.8035	442.25	LC(dppz)
T_3	193 ->197<br>193 ->198<br>196 ->199	0.66755 (89%)<br>0.15831 (5%)<br>-0.1037 (2%)	2.962	418.58	LC(dppz)
T_{21}	191 ->201<br>192 ->197<br>192 ->198<br>192 ->200<br>195 ->200<br>195 ->202	-0.26435 (14%)<br>0.11129 (2%)<br>-0.15072 (5%)<br>0.44190 (39%)<br>0.24400 (12%)<br>0.25713 (13%)	3.9581	313.24	LC(trz)/<br>LMCT
T_{24}	189 ->197<br>189 ->198<br>191 ->206<br>191 ->208<br>192 ->198	0.21075 (9%)<br>-0.10307 (2%)<br>0.15541 (5%)<br>0.10290 (2%)<br>0.19862 (8%)	4.0740	304.33	LMCT

	192 ->202	0.24906 (12%)			
	192 ->207	-0.13594 (4%)			
	194 ->201	0.23984 (11%)			
	194 ->206	0.12272 (3%)			
	195 ->202	0.38004 (29%)			

Table S12. Energies, free energies, enthalpies and entropies of the optimized structures.[a]

	E_0 [b]	ZPE [c]	G [d]	H [e]	S [f]
**2 (S_0)**	-1952.869206	-1952.159244	-1952.234023	-1952.116988	246.322
**2 (T_1)**	-1952.762891	-1952.057684	-1952.134314	-1952.014762	251.619
**5 (S_0)**	-2029.103159	-2028.380562	-2028.455672	-2028.337681	248.332
**5 (T_1)**	-2029.999471	-2028.282917	-2028.359746	-2028.239319	253.458
**7 (S_0)**	-2368.471174	-2367.679097	-2367.760990	-2367.630963	273.666
**7 (T_1)**	-2368.390435	-2367.602460	-2367.685789	-2367.553830	277.731

[a] Thermal corrections from vibrational calculations at 298.15 K. [b] Electronic energy (Hartrees). [c] Sum of electronic and zero-point energies (Hartrees). [d] Free Energy (Hartrees). [e] Enthalpy (Hartrees). [f] Entropy (cal mol^{-1} K^{-1}).

Table S13. Cartesian coordinates (Å) of the optimized structures.

2 (S_0)

C	-49.037726189	20.978201358	-81.382984586
C	-48.582880510	20.047195164	-82.307979538
C	-47.618199170	20.160025491	-83.446296422
C	-48.296466076	20.406141119	-84.811550441
C	-47.282496637	20.492200422	-85.958252668
C	-47.942538785	20.739631109	-87.317257633
C	-49.142574141	17.579776169	-82.671346504
C	-50.620184661	20.889410337	-79.556944186
C	-50.280617494	22.242186409	-79.390547046
C	-50.913127343	22.939330285	-78.360757007
C	-51.836088561	22.289786802	-77.529943963
C	-52.145415629	20.940976490	-77.719163989
C	-51.533689799	20.221814290	-78.745910200
C	-48.929588712	24.892480648	-79.997714967
C	-48.358634348	25.809927997	-79.125066758
C	-47.272749827	25.679245400	-78.103809172
C	-47.793249575	25.446875944	-76.668367729
C	-46.653880792	25.348453262	-75.647144541
C	-47.152826991	25.104503238	-74.220564171
C	-48.833247549	28.283115289	-78.689530069
C	-50.716898894	25.011278294	-81.620711115
C	-50.420159206	23.654214775	-81.830483856
C	-51.180203101	22.971548871	-82.780718446
C	-52.184358471	23.639224718	-83.494979671
C	-52.449128233	24.991245019	-83.265699869
C	-51.709619167	25.696055245	-82.316008744
C	-47.377603600	24.129319476	-83.213783877
C	-46.299520528	24.535791369	-83.993130199
C	-45.010706798	24.306556812	-83.516604499
C	-44.840830741	23.676944420	-82.286176906
C	-45.963078258	23.284008404	-81.550391396
C	-45.874158647	22.599083337	-80.238457277
C	-44.661758747	22.220953225	-79.652337910
C	-44.666518961	21.580678970	-78.415592470
C	-45.881666402	21.324693868	-77.784231623
C	-47.055285072	21.721041224	-78.417209635
H	-50.698848605	23.988883824	-78.194147666
H	-52.317776332	22.847702239	-76.732329913
H	-52.863659390	20.447991843	-77.071960104
H	-51.758209175	19.174281784	-78.915662853
H	-51.003316239	21.919996206	-82.976186996
H	-52.764417277	23.092825978	-84.232915757
H	-53.231234374	25.498145864	-83.821690529
H	-51.896107474	26.746124467	-82.118120795
H	-48.401080608	24.286861099	-83.533261411
H	-44.143587432	24.614196757	-84.091824931
H	-43.843135566	23.500169902	-81.905883676
H	-43.721409188	22.416890044	-80.152034165
H	-43.729662025	21.284628006	-77.955206676
H	-45.930711238	20.826672564	-76.822741433
H	-48.026963141	21.547253912	-77.970023109
H	-46.634016060	24.842292313	-78.396307663
H	-46.645933766	26.578485825	-78.125559416
H	-48.389345467	24.525843154	-76.648922053
H	-48.470737320	26.262362004	-76.385294944
H	-45.973742132	24.538010632	-75.941972470
H	-46.061891526	26.272973119	-75.676761004
H	-47.810040528	25.915167911	-73.884817611
H	-47.719261970	24.168208024	-74.151613962
H	-46.318028661	25.040370978	-73.514436400
H	-49.027840205	28.196896387	-77.619414606
H	-47.804058073	28.606647380	-78.856206085
H	-49.526223889	28.990410266	-79.141962858
H	-46.933778831	20.982902728	-83.225101920
H	-47.010203774	19.249284140	-83.497739859
H	-48.878142841	21.335108552	-84.760816101
H	-49.013119076	19.600691065	-85.016200217
H	-46.564598006	21.295970867	-85.745739318
H	-46.700563909	19.561604181	-85.995835895
H	-48.505405310	21.680540802	-87.321541323
H	-47.195636954	20.795563874	-88.116390422
H	-48.641301096	19.934252599	-87.572055949
H	-49.798472408	16.888765722	-82.144563253
H	-49.450622770	17.669107533	-83.714278131
H	-48.109399008	17.232321774	-82.616146967

N	-49.933896274	20.264461055	-80.630914705
N	-50.079814307	19.002560539	-80.989795835
N	-49.252866427	18.882762747	-82.014779887
N	-49.898121564	25.622414198	-80.635087267
N	-49.984140437	26.883594980	-80.254382866
N	-49.042040889	26.985117953	-79.332024805
N	-47.047390753	22.341225794	-79.606662505
Pt	-48.863487277	22.936505537	-80.702477990
N	-47.210159430	23.521665241	-82.029528586
H	-46.477385944	25.023555100	-84.944815264

2 (T_1)

C	-49.056642953	20.981901947	-81.384715525
C	-48.598940510	20.050029967	-82.307655152
C	-47.626911967	20.160147148	-83.440133095
C	-48.294904368	20.405093243	-84.810536537
C	-47.272891594	20.485286192	-85.950576952
C	-47.922754382	20.731330487	-87.314719169
C	-49.156966069	17.582128468	-82.671020531
C	-50.640142475	20.890774715	-79.558590985
C	-50.294717951	22.241233678	-79.385206060
C	-50.925597140	22.934534246	-78.351435549
C	-51.851915618	22.284408350	-77.524892966
C	-52.166866200	20.937953671	-77.721970216
C	-51.557211780	20.222142115	-78.752276203
C	-48.945420690	24.894420717	-79.991997207
C	-48.371270254	25.811286977	-79.120816935
C	-47.280374213	25.680132037	-78.105105127
C	-47.794540661	25.440377912	-76.668797421
C	-46.651989300	25.342902669	-75.651015472
C	-47.146457920	25.094346687	-74.223663032
C	-48.845808153	28.283081739	-78.678476085
C	-50.734397621	25.017534333	-81.613563518
C	-50.434643740	23.662137246	-81.829788517
C	-51.193063325	22.984136040	-82.785000019
C	-52.198288427	23.653503296	-83.496099410
C	-52.466177795	25.003623481	-83.259083566
C	-51.728308914	25.704470486	-82.305175428
C	-47.372575305	24.115092334	-83.201252430
C	-46.270648776	24.509831679	-84.000483647
C	-44.963400045	24.266093009	-83.522199490
C	-44.796149765	23.655643236	-82.307852535
C	-45.954686724	23.261407138	-81.513454824
C	-45.868598053	22.638269124	-80.278707557
C	-44.609377925	22.263854524	-79.642344398
C	-44.609123444	21.624263701	-78.431520176
C	-45.840137948	21.333914464	-77.800875835
C	-47.042703406	21.723460622	-78.442543996
H	-50.707786991	23.982565554	-78.179224868
H	-52.332145453	22.839798024	-76.724599162
H	-52.887912006	20.444387670	-77.078297531
H	-51.786178150	19.176596670	-78.928313894
H	-51.013882797	21.933885627	-82.985901103
H	-52.776787112	23.110230459	-84.237610088
H	-53.249141732	25.512093071	-83.812463266
H	-51.916939066	26.753078333	-82.101607290
H	-48.386148919	24.292413333	-83.547353931
H	-44.100719736	24.561024921	-84.109968832
H	-43.802415059	23.465966001	-81.924011172
H	-43.675482212	22.490730697	-80.140147257
H	-43.673224054	21.342528411	-77.961101181
H	-45.890041320	20.827529090	-76.844732981
H	-47.999027133	21.519301606	-77.971287843
H	-46.639516487	24.847228849	-78.405031150
H	-46.656967218	26.581750372	-78.125632442
H	-48.387494882	24.517189836	-76.650467251
H	-48.474288153	26.252377329	-76.380923939
H	-45.970000373	24.535273198	-75.949439690
H	-46.062855317	26.269248611	-75.680096408
H	-47.804990272	25.902518881	-73.884508042
H	-47.710301729	24.156429914	-74.155270048
H	-46.309706396	25.030924476	-73.519800140
H	-49.034948749	28.193336136	-77.607663696
H	-47.818067935	28.609148113	-78.849184961
H	-49.542328446	28.990474350	-79.125324996
H	-46.942443791	20.981984605	-83.215128516
H	-47.019821359	19.248476933	-83.486186862
H	-48.874725150	21.335648313	-84.766123749
H	-49.012370608	19.601235165	-85.018418528
H	-46.553581277	21.287133078	-85.735369032
H	-46.693651478	19.552793433	-85.981855561
H	-48.482795047	21.673888621	-87.325282077
H	-47.170192048	20.782922782	-88.108808370
H	-48.622169698	19.927400601	-87.572259682
H	-49.817839660	16.892513726	-82.148654852
H	-49.456745933	17.670684640	-83.716412303
H	-48.124704137	17.233177507	-82.607569533
N	-49.954985046	20.268118569	-80.634983019
N	-50.100237268	19.006051098	-80.994028195
N	-49.270392619	18.885861768	-82.016565860
N	-49.915601876	25.625551902	-80.625759460
N	-50.000514874	26.886398349	-80.243079111
N	-49.055527923	26.986622192	-79.323517222
N	-47.077776163	22.342623933	-79.603535068
Pt	-48.881049029	22.939422362	-80.700188240
N	-47.245286745	23.525326301	-82.031117176
H	-46.453147827	24.991571522	-84.953435562

5 (S_0)

C	-49.026724541	20.978113832	-81.401732807
C	-48.585210160	20.051697087	-82.337708716
C	-47.643738269	20.170049060	-83.494321374

C -48.351864954 20.403762785 -84.846472435
C -47.363092766 20.485836166 -86.015224420
C -48.052765242 20.724129179 -87.361057104
C -49.152722914 17.587731396 -82.708778915
C -50.579022926 20.878664705 -79.550807317
C -50.243425065 22.232727630 -79.387550315
C -50.864419647 22.926721261 -78.348823001
C -51.771736586 22.271935749 -77.504929225
C -52.076393242 20.921522400 -77.689963459
C -51.476661690 20.206041792 -78.726321646
C -48.911118045 24.890365156 -80.010087660
C -48.335095433 25.806898881 -79.139820749
C -47.242528038 25.675515600 -78.126025092
C -47.753090578 25.443597162 -76.687016994
C -46.606918745 25.349909981 -75.672929760
C -47.095814122 25.104692737 -74.243105005
C -48.807347551 28.279565833 -78.698648322
C -50.706946181 25.011341055 -81.623202481
C -50.409576032 23.655337636 -81.837763218
C -51.174405329 22.972783177 -82.784033741
C -52.184323334 23.640154694 -83.490470022
C -52.449816851 24.991272748 -83.256935636
C -51.705362360 25.695623119 -82.310750310
C -47.327854695 24.122351905 -83.260600188
C -46.207256199 24.480635012 -84.028921103
C -44.941691131 24.209047437 -83.547415845
C -44.792174004 23.579268724 -82.290971252
C -45.971648125 23.257263098 -81.576358653
C -45.874580507 22.618654111 -80.292994814
C -44.600921990 22.303226158 -79.760190584
C -44.560100199 21.665204346 -78.499628463
C -45.739606071 21.377787385 -77.841363187
C -46.963199764 21.731664299 -78.434814304
H -50.653783606 23.977470635 -78.185290298
H -52.244892327 22.827115180 -76.700347498
H -52.782092061 20.424443244 -77.032190847
H -51.698492792 19.157512475 -78.893265956
H -50.997309865 21.921852824 -82.982110606
H -52.768495752 23.094025252 -84.225345347
H -53.236548901 25.497827987 -83.806666129
H -51.892578709 26.744906090 -82.109440172
H -48.334847090 24.316171433 -83.612344305
H -44.057853574 24.472757362 -84.120435429
H -43.600013810 21.407752206 -78.062874666
H -45.742894326 20.887445767 -76.874661130
H -47.906402805 21.527841309 -77.940679173
H -46.606699824 24.838191983 -78.423567171
H -46.615316206 26.574365280 -78.152175973
H -48.346518306 24.520922652 -76.662551542
H -48.431087498 26.257445894 -76.400491856
H -45.925303893 24.542173564 -75.971692122
H -46.018773000 26.276733241 -75.706715611
H -47.754244237 25.912828260 -73.903668492
H -47.657898100 24.166095601 -74.169943604
H -46.256286323 25.044492739 -73.542252602
H -48.996649228 28.192419706 -77.627659188
H -47.778924958 28.602977589 -78.870137205
H -49.502427000 28.987470475 -79.146912782
H -46.961921536 20.999081528 -83.289004306
H -47.029200920 19.264137457 -83.554202014
H -48.936393138 21.330647059 -84.789637211
H -49.069658437 19.593701639 -85.028971381
H -46.642540417 21.292300018 -85.822672694
H -46.779919700 19.556343525 -86.060108705
H -48.617417634 21.663985703 -87.358726608
H -47.323393466 20.776734583 -88.176430873
H -48.755372396 19.916035143 -87.595958356
H -49.737058742 16.873754657 -82.130911539
H -49.552696140 17.670612619 -83.720785424
H -48.108143111 17.274720461 -82.746155234
N -49.908114441 20.259004497 -80.637410355
N -50.056273223 18.997820328 -80.998066876
N -49.245900444 18.884091088 -82.036870920
N -49.883402816 25.621010767 -80.640870116
N -49.967242378 26.881769107 -80.258286078
N -49.019738449 26.982284367 -79.341381188
N -47.024844098 22.334638966 -79.619252161
Pt -48.849491227 22.935385851 -80.717995583
N -47.210313103 23.528716316 -82.075620983
H -46.356064651 24.964045838 -84.987763290
C -43.515260147 23.254530744 -81.721916793
C -43.423664698 22.639327161 -80.508815521
H -42.622115739 23.509556819 -82.283717819
H -42.456421293 22.391629557 -80.082963834

5 (T_1)

C -49.018699250 20.976616948 -81.398085717
C -48.578721124 20.049003998 -82.333438784
C -47.636335291 20.165628737 -83.489493690
C -48.342818140 20.403844596 -84.841755349
C -47.352665434 20.485420444 -86.009367486
C -48.040598822 20.726626843 -87.355570066
C -49.152152408 17.586055863 -82.703214617
C -50.573983201 20.883383326 -79.549672232
C -50.237530559 22.237571613 -79.389416194
C -50.858599438 22.934466035 -78.352755317
C -51.766997433 22.282328684 -77.507979932
C -52.072521357 20.931711390 -77.689955521
C -51.472592634 20.213354598 -78.724172657
C -48.901672072 24.890595877 -80.013199222
C -48.327391882 25.806558408 -79.141382351
C -47.236889602 25.674283781 -78.125474747

C	-47.750434531	25.441784571	-76.687551992
C	-46.605956013	25.346995273	-75.671695190
C	-47.097357468	25.101324894	-74.242811840
C	-48.802487163	28.278486787	-78.699033563
C	-50.698155700	25.009052098	-81.625149324
C	-50.401899204	23.652335931	-81.836722855
C	-51.167477182	22.967956604	-82.780969805
C	-52.177013226	23.634517528	-83.488717917
C	-52.441314560	24.986428924	-83.258496708
C	-51.696136313	25.692455146	-82.314166149
C	-47.352475883	24.133076475	-83.281173541
C	-46.249620330	24.504140801	-84.050173390
C	-44.967184192	24.248330645	-83.590920565
C	-44.786696331	23.603980402	-82.325569919
C	-45.979370303	23.266562912	-81.597046837
C	-45.876942067	22.609274338	-80.280975611
C	-44.585435936	22.280924512	-79.741131207
C	-44.568147186	21.628274547	-78.467243344
C	-45.764551910	21.354656518	-77.823851124
C	-46.973678320	21.719287531	-78.416398936
H	-50.646949917	23.985258061	-78.191079497
H	-52.240109887	22.839759235	-76.704940227
H	-52.778870060	20.436658295	-77.031365859
H	-51.694752991	19.164501689	-78.888549928
H	-50.991265867	21.916520887	-82.976974941
H	-52.761780122	23.087032492	-84.222095670
H	-53.227697353	25.492279934	-83.809361229
H	-51.882381728	26.742384243	-82.115352140
H	-48.370483315	24.308749246	-83.603028140
H	-44.095038806	24.527064654	-84.173180113
H	-43.616028407	21.357342358	-78.023195898
H	-45.777718210	20.860236107	-76.858711198
H	-47.929764869	21.532480342	-77.945462406
H	-46.601578712	24.835984150	-78.421244083
H	-46.608960787	26.572683555	-78.150153987
H	-48.343654134	24.519016662	-76.665031796
H	-48.428725780	26.255629229	-76.401708963
H	-45.924612788	24.538808102	-75.969790789
H	-46.017167476	26.273488696	-75.704033211
H	-47.755841400	25.909677274	-73.903967477
H	-47.660085930	24.163016213	-74.171194050
H	-46.259001426	25.040182961	-73.540633319
H	-48.993623340	28.190680565	-77.628428082
H	-47.773945347	28.602499318	-78.868634871
H	-49.497175816	28.986202408	-79.148197095
H	-46.952676439	20.992865495	-83.283082813
H	-47.024373130	19.258043505	-83.550151162
H	-48.924048438	21.332625652	-84.783422553
H	-49.062811307	19.596263968	-85.026464668
H	-46.631106008	21.290504519	-85.815029787
H	-46.771116095	19.554908608	-86.054857679
H	-48.603770205	21.667350265	-87.352420403
H	-47.310296329	20.779310195	-88.170113555
H	-48.744180880	19.919922215	-87.592358363
H	-49.749364388	16.877243840	-82.132179976
H	-49.539716216	17.672077064	-83.719735007
H	-48.109889714	17.264204809	-82.728198662
N	-49.902631635	20.260560648	-80.634119402
N	-50.053833557	18.999773415	-80.994544143
N	-49.242878366	18.883355161	-82.032669253
N	-49.874332453	25.620252456	-80.644100214
N	-49.959762828	26.880515592	-80.260541431
N	-49.013050601	26.981386671	-79.342784037
N	-47.006957219	22.340762976	-79.631179816
Pt	-48.838483387	22.934970764	-80.718712089
N	-47.196258896	23.521257563	-82.070613908
H	-46.412366941	24.991648570	-85.005317687
C	-43.547720885	23.288169113	-81.777192002
C	-43.445733612	22.610108269	-80.467684690
H	-42.637398748	23.535029665	-82.312264815
H	-42.463574817	22.371652533	-80.074871127

7 (S_0)

C	-49.022942776	20.980916884	-81.400447435
C	-48.579405033	20.053729921	-82.334736327
C	-47.635239788	20.170432132	-83.489372509
C	-48.339854377	20.405523675	-84.843128035
C	-47.348214948	20.485448120	-86.009591328
C	-48.034316112	20.724996077	-87.357009428
C	-49.145961352	17.589261883	-82.704512159
C	-50.578963552	20.882764929	-79.552459409
C	-50.244340488	22.237080484	-79.389338934
C	-50.866812216	22.931620225	-78.351934563
C	-51.775030397	22.276851699	-77.508969494
C	-52.078955571	20.926267162	-77.693747605
C	-51.477511337	20.210377341	-78.728823596
C	-48.910467686	24.893731940	-80.007949255
C	-48.335470302	25.810323452	-79.137126178
C	-47.242407227	25.679875545	-78.123715233
C	-47.752892295	25.445657990	-76.684984424
C	-46.607036857	25.353552361	-75.670426223
C	-47.096388332	25.107264538	-74.240935907
C	-48.812656257	28.281570515	-78.692605647
C	-50.708713633	25.012366124	-81.618338813
C	-50.409854167	23.656790334	-81.833643044
C	-51.174189160	22.973079358	-82.779384390
C	-52.185415344	23.639353255	-83.485002034
C	-52.452648320	24.990005845	-83.250906190
C	-51.708549012	25.695278360	-82.305130977
C	-47.344982786	24.129268833	-83.255875164
C	-46.240142554	24.489314419	-84.034806612
C	-44.965664500	24.212447665	-83.568773438

C	-44.807201315	23.580763174	-82.322914507
C	-45.965145172	23.257574572	-81.591732840
C	-45.864803429	22.609158554	-80.293108587
C	-44.608608608	22.277406065	-79.753032932
C	-44.572987464	21.645571658	-78.497813914
C	-45.760099624	21.371545316	-77.839433277
C	-46.971502078	21.736088644	-78.436784960
H	-50.656760969	23.982473318	-78.188514575
H	-52.249381602	22.832285242	-76.705288955
H	-52.785345020	20.429285110	-77.036659219
H	-51.698570952	19.161630532	-78.895411240
H	-50.996014334	21.922434443	-82.977829562
H	-52.769272478	23.092550806	-84.219600070
H	-53.240448408	25.495564696	-83.799998496
H	-51.896998450	26.744288487	-82.103589588
H	-48.358699860	24.321789232	-83.587799477
H	-44.084523208	24.472774349	-84.144090710
H	-43.613501586	21.383491122	-78.066728664
H	-45.768492005	20.883695805	-76.871549567
H	-47.921513779	21.545107939	-77.951244726
H	-46.604923958	24.843931810	-78.421830940
H	-46.616939986	26.579933336	-78.148910420
H	-48.344587656	24.521835483	-76.661467787
H	-48.432641533	26.257930250	-76.398174059
H	-45.923883581	24.547038872	-75.968921879
H	-46.020430594	26.281348936	-75.703678663
H	-47.756207538	25.914345781	-73.901670318
H	-47.657089517	24.167796800	-74.168254470
H	-46.257116655	25.048166023	-73.539708847
H	-49.002476211	28.193106049	-77.621817071
H	-47.784589113	28.606527680	-78.863210275
H	-49.508497404	28.988856232	-79.140657378
H	-46.952140700	20.998131443	-83.282915063
H	-47.022159687	19.263477044	-83.547942376
H	-48.922654162	21.333593253	-84.787726352
H	-49.058837715	19.596878184	-85.027103914
H	-46.626442210	21.290447104	-85.815588599
H	-46.766805470	19.554801343	-86.053010322
H	-48.597343824	21.665832955	-87.355961657
H	-47.302841968	20.776450891	-88.170552772
H	-48.737716133	19.918105444	-87.593663844
H	-49.736140037	16.877217963	-82.130223079
H	-49.538690194	17.672443572	-83.719302669
H	-48.101906440	17.273730000	-82.734296371
N	-49.905865967	20.262487594	-80.637274357
N	-50.053305385	19.001111175	-80.997263056
N	-49.240886331	18.886430210	-82.034265252
N	-49.884952194	25.622977878	-80.636872711
N	-49.970999431	26.883036963	-80.252722308
N	-49.022813233	26.984526756	-79.336699027
N	-47.016978513	22.339711606	-79.626857496
Pt	-48.847040713	22.938937900	-80.716839860
N	-47.205919927	23.529479813	-82.071116097
H	-46.397621682	24.977264580	-84.989935033
C	-43.491407218	23.248647299	-81.775537794
C	-43.393222507	22.596812161	-80.502115608
C	-41.206553210	23.235581278	-81.961743097
C	-41.108161485	22.578989524	-80.683704369
C	-40.019418189	23.544560571	-82.680903445
C	-38.795899375	23.213647939	-82.149719556
C	-38.698658399	22.564039579	-80.885925221
H	-37.717459002	22.315532810	-80.493271469
C	-39.825687086	22.251212903	-80.164363466
H	-40.113850240	24.039409933	-83.642218478
H	-37.886820752	23.447483269	-82.695340459
H	-39.771549290	21.756172032	-79.200113088
N	-42.411072549	23.557374439	-82.481530096
N	-42.218182891	22.272594680	-79.977698308

7 (T_1)

C	-49.029920348	20.981563803	-81.400739012
C	-48.585266994	20.055291100	-82.335378748
C	-47.641939119	20.174245224	-83.490412342
C	-48.347675511	20.405685233	-84.844145114
C	-47.356436299	20.488277948	-86.010787269
C	-48.043383299	20.725145781	-87.358254582
C	-49.146289547	17.589840274	-82.704749231
C	-50.582983765	20.880076021	-79.550551725
C	-50.249101073	22.234567209	-79.386976687
C	-50.871157396	22.927340504	-78.348025531
C	-51.777872290	22.271211305	-77.504531284
C	-52.080902996	20.920494367	-77.690094166
C	-51.480028950	20.206083015	-78.726534905
C	-48.917770385	24.893415598	-80.008510712
C	-48.340861560	25.810354514	-79.139289984
C	-47.246532905	25.679506708	-78.127376081
C	-47.755077850	25.447190064	-76.687729796
C	-46.607673193	25.353813828	-75.675027419
C	-47.094970667	25.109535926	-74.244487216
C	-48.814103768	28.282630140	-78.697294687
C	-50.716004690	25.013116338	-81.619016160
C	-50.417396901	23.657489853	-81.834381775
C	-51.182704998	22.974549624	-82.780035807
C	-52.194247841	23.641055195	-83.484928478
C	-52.460957779	24.991794729	-83.250506132
C	-51.716053857	25.696598234	-82.305006091
C	-47.343045257	24.129892998	-83.255082457
C	-46.225123181	24.486410347	-84.029689468
C	-44.958180452	24.207283742	-83.559795417
C	-44.802768979	23.571501672	-82.306039574
C	-45.978380953	23.252854999	-81.581303285
C	-45.880228046	22.612931271	-80.300302222

C	-44.608465359	22.284762476	-79.767829786
C	-44.570568860	21.648258503	-78.505415016
C	-45.750744988	21.373091519	-77.845760120
C	-46.973423786	21.735426537	-78.438023764
H	-50.661594315	23.978242229	-78.183953596
H	-52.251766022	22.825496321	-76.699756936
H	-52.786089308	20.422322936	-77.032586671
H	-51.700392909	19.157282918	-78.893788253
H	-51.004668763	21.923895713	-82.978822828
H	-52.778714217	23.094590466	-84.219329131
H	-53.248908894	25.497715273	-83.799081961
H	-51.904081964	26.745633179	-82.103152488
H	-48.352103190	24.328379764	-83.597970793
H	-44.073208181	24.464761865	-84.129906114
H	-43.609075018	21.387393275	-78.078743104
H	-45.755948917	20.884717064	-76.878015055
H	-47.918137152	21.539480510	-77.943802377
H	-46.610572661	24.842642946	-78.426046896
H	-46.619991116	26.578790313	-78.154321787
H	-48.348118750	24.524281817	-76.662618218
H	-48.433100224	26.260739017	-76.400304527
H	-45.926563282	24.545848382	-75.974226352
H	-46.019432937	26.280543476	-75.709999156
H	-47.752942038	25.917958263	-73.904762830
H	-47.657060148	24.171041662	-74.170072723
H	-46.254634443	25.049645391	-73.544576748
H	-49.001976667	28.195577942	-77.626039906
H	-47.786073455	28.606530432	-78.870155727
H	-49.510093456	28.990167744	-79.144740509
H	-46.961959584	21.004623390	-83.284679870
H	-47.025638446	19.269393192	-83.548275228
H	-48.933520340	21.331859668	-84.789292717
H	-49.063984493	19.594459315	-85.027468517
H	-46.637242083	21.295610498	-85.817060770
H	-46.772087665	19.559431962	-86.053888756
H	-48.609105000	21.664361896	-87.357529080
H	-47.312188455	20.778393554	-88.171959325
H	-48.744513997	19.916136745	-87.594494123
H	-49.729243588	16.874755490	-82.126858041
H	-49.545187062	17.670507524	-83.717362176
H	-48.100774424	17.279774315	-82.740485629
N	-49.910639622	20.261252494	-80.636811327
N	-50.055686788	18.999395159	-80.996540200
N	-49.243845067	18.886581820	-82.034334574
N	-49.891661526	25.623348778	-80.637641077
N	-49.975772451	26.884074044	-80.254911514
N	-49.026728404	26.985217332	-79.339604210
N	-47.030082000	22.337419740	-79.623049537
Pt	-48.855041661	22.938883818	-80.716781651
N	-47.217885894	23.531697383	-82.073374625
H	-46.377912422	24.974569110	-84.985553446
C	-43.509280815	23.241399846	-81.759044927
C	-43.414141519	22.602211410	-80.511642915
C	-41.207018991	23.237216964	-81.957564184
C	-41.109832997	22.576754544	-80.679307964
C	-40.014535682	23.539115972	-82.655545272
C	-38.724911729	23.203160916	-82.121583202
C	-38.632888864	22.575222153	-80.909557218
H	-37.669895070	22.311378874	-80.485971468
C	-39.826469422	22.256810910	-80.178375551
H	-40.097521999	24.036410768	-83.616989953
H	-37.837212661	23.454467985	-82.691993942
H	-39.763699528	21.758959420	-79.215754509
N	-42.397191542	23.569553771	-82.499038454
N	-42.203491132	22.259202950	-79.956307906

6. References

(1) Sheldrick, G. M. A Short History of SHELX. *Acta Crystallogr., Sect. A: Found. Crystallogr.* **2008**, *64*, 112–122.

(2) Sheldrick, G. M. SHELXT – Integrated Space-Group and Crystal-Structure Determination. *Acta Crystallogr., Sect. A: Found. Crystallogr.* **2015**, *71*, 3–8.

(3) Cardona, C. M.; Li, W.; Kaifer, A. E.; Stockdale, D.; Bazan, G. C. Electrochemical Considerations for Determining Absolute Frontier Orbital Energy Levels of Conjugated Polymers for Solar Cell Applications. *Adv. Mater.* **2011**, *23*, 2367–2371.

(4) Juliá, F.; Aullón, G.; Bautista, D.; González-Herrero, P. Exploring Excited-State Tunability in Luminescent Tris-Cyclometalated Platinum(IV) Complexes: Synthesis of Heteroleptic Derivatives and Computational Calculations. *Chem. Eur. J.* **2014**, *20*, 17346–17359.

(5) Vivancos, Á.; Jiménez-García, A.; Bautista, D.; González-Herrero, P. Strongly Luminescent Pt(IV) Complexes with a Mesoionic N-Heterocyclic Carbene Ligand: Tuning Their Photophysical Properties. *Inorg. Chem.* **2021**, *60*, 7900–7913.

(6) Vivancos, Á.; Bautista, D.; González-Herrero, P. Phosphorescent Tris-Cyclometalated Pt(IV) Complexes with Mesoionic N-Heterocyclic Carbene and 2-Arylpyridine Ligands. *Inorg. Chem.* **2022**, *61*, 12033–12042.

(7) López-López, J. C.; Bautista, D.; González-Herrero, P. Stereoselective Formation of Facial Tris-Cyclometalated Pt(IV) Complexes: Dual Phosphorescence from Heteroleptic Derivatives. *Chem. Eur. J.* **2020**, *26*, 11307–11315.

(8) Jenkins, D. M.; Bernhard, S. Synthesis and Characterization of Luminescent Bis-Cyclometalated Platinum(IV) Complexes. *Inorg. Chem.* **2010**, *49*, 11297–11308.

(9) Juliá, F.; González-Herrero, P. Spotlight on the Ligand: Luminescent Cyclometalated Pt(IV) Complexes Containing a Fluorenyl Moiety. *Dalton Trans.* **2016**, *45*, 10599–10608.

(10) Corral-Zorzano, A.; Gómez de Segura, D.; Lalinde, E.; Moreno, M. T. Phosphorescent 2-Phenylbenzothiazole Pt(IV) Bis-Cyclometalated Complexes with Phenanthroline-Based Ligands. *Dalton Trans.* **2023**, *52*, 6543–6550.

(11) Juliá, F.; Bautista, D.; González-Herrero, P. Developing Strongly Luminescent Platinum(IV) Complexes: Facile Synthesis of Bis-Cyclometalated Neutral Emitters. *Chem. Commun.* **2016**, *52*, 1657–1660.

(12) Frisch, M. J.; Trucks, G. W.; Schlegel, H. B.; Scuseria, G. E.; Robb, M. A.; Cheeseman, J. R.; Scalmani, G.; Barone, V.; Petersson, G. A.; Nakatsuji, H.; Li, X.; Caricato, M.; Marenich, A. V.; Bloino, J.; Janesko, B. G.; Gomperts, R.; Mennucci, B.; Hratchian, H. P.; Ortiz, J. V.; et al. Gaussian 16 (Revision A.03). Gaussian Inc.: Wallingford CT 2016.

(13) Becke, A. Density Functional Thermochemistry III The Role of Exact Exchange. *J. Chem. Phys.* **1993**, *98*, 5648–5652.

(14) Lee, C. T.; Yang, W. T.; Parr, R. G. Development of The Colle-Salvetti Correlation-Energy Formula into a Functional of the Electron-Density. *Phys. Rev. B* **1988**, *37*, 785–789.

(15) Hariharan, P. C.; Pople, J. A. Influence of Polarization Functions on Molecular-Orbital Hydrogenation Energies. *Theoret. Chim. Acta* **1973**, *28*, 213–222.

(16) Francl, M. M.; Pietro, W. J.; Hehre, W. J.; Binkley, J. S.; Gordon, M. S.; Defrees, D. J.; Pople, J. A. Self-Consistent Molecular-Orbital Methods. 23. A Polarization-Type Basis Set for 2nd-Row Elements. *J. Chem. Phys.* **1982**, *77*, 3654–3665.

(17) Hay, P. J.; Wadt, W. R. Ab Initio Effective Core Potentials for Molecular Calculations–Potentials for K to Au Including the Outermost Core Orbitals. *J. Chem. Phys.* **1985**, *82*, 299–310.

(18) Marenich, A. V.; Cramer, C. J.; Truhlar, D. G. Universal Solvation Model Based on Solute Electron Density and on a Continuum Model of the Solvent Defined by the Bulk Dielectric Constant and Atomic Surface Tensions. *J. Phys. Chem. B* **2009**, *113*, 6378–6396.
